# ChatGPT and large language models (LLMs) awareness and use. A prospective cross-sectional survey of U.S. medical students

**DOI:** 10.1371/journal.pdig.0000596

**Published:** 2024-09-05

**Authors:** Conner Ganjavi, Michael Eppler, Devon O’Brien, Lorenzo Storino Ramacciotti, Muhammad Shabbeer Ghauri, Issac Anderson, Jae Choi, Darby Dwyer, Claudia Stephens, Victoria Shi, Madeline Ebert, Michaela Derby, Bayan Yazdi, Giovanni E. Cacciamani

**Affiliations:** 1 USC Institute of Urology and Catherine and Joseph Aresty Department of Urology, Keck School of Medicine, University of Southern California, Los Angeles, California United States of America; 2 AI Center at USC Urology, USC Institute of Urology, University of Southern California, Los Angeles, California, United States of America; 3 Keck School of Medicine, University of Southern California, Los Angeles, California, United States of America; 4 California University of Science and Medicine, Colton, California, United States of America; 5 Wayne State University School of Medicine, Detroid, Michigan, United States of America; 6 UT Southwestern Medical School, Dallas, Texas, United States of America; 7 Texas A&M School of Medicine, Bryan, Texas, United States of America; 8 Frederick P. Whiddon College of Medicine, University of South Alabama, Mobile, Alabama, United States of America; 9 University of Missouri–Kansas City School of Medicine, Kansas City, Missouri, United States of America; 10 Medical College of Wisconsin, Milwaukee, Wisconsin, United States of America; 11 Sanford School of Medicine, University of South Dakota, Vermillion, South Dakota, United States of America; 12 Stritch School of Medicine, Loyola University Chicago, Maywood, Illinois, United States of America; Telemedicine and Advanced Technology Research Center, UNITED STATES OF AMERICA

## Abstract

Generative-AI (GAI) models like ChatGPT are becoming widely discussed and utilized tools in medical education. For example, it can be used to assist with studying for exams, shown capable of passing the USMLE board exams. However, there have been concerns expressed regarding its fair and ethical use. We designed an electronic survey for students across North American medical colleges to gauge their views on and current use of ChatGPT and similar technologies in May, 2023. Overall, 415 students from at least 28 medical schools completed the questionnaire and 96% of respondents had heard of ChatGPT and 52% had used it for medical school coursework. The most common use in pre-clerkship and clerkship phase was asking for explanations of medical concepts and assisting with diagnosis/treatment plans, respectively. The most common use in academic research was for proof reading and grammar edits. Respondents recognized the potential limitations of ChatGPT, including inaccurate responses, patient privacy, and plagiarism. Students recognized the importance of regulations to ensure proper use of this novel technology. Understanding the views of students is essential to crafting workable instructional courses, guidelines, and regulations that ensure the safe, productive use of generative-AI in medical school.

## Author summary

The study examines how medical students in the US are using a popular artificial-intelligence program called ChatGPT. ChatGPT can assist with various tasks such as answering questions, helping with writing assignments, and even aiding in diagnosis and treatment planning. The study found that most medical students are familiar with ChatGPT and have incorporated it into their academic journey, finding it particularly beneficial for studying, writing, and clinical rotations. Students are hopeful about the future integration of ChatGPT and similar models with existing study resources. However, students noted common flaws with ChatGPT use, including the risk of bias and inaccuracy. There are also worries about privacy if ChatGPT collects information from patient data provided. Therefore, students are calling for clear rules and guidelines to ensure responsible and ethical use of this technology. Additionally, the study highlights that if ChatGPT becomes a paid service, it might limit access for some students, raising concerns about affordability and equity. In summary, ChatGPT has proven to be a valuable tool for medical students, but there are important issues to address to ensure a smooth integration in medical education.

## Introduction

The usage of generative-AI (GAI) technologies like large language models (LLMs) has dramatically increased in the last year since the launch of ChatGPT in Nov 2022 [1]. ChatGPT’s ability to process data and generate clear and understandable responses has led to its adoption by millions worldwide [2]. Among specialty fields looking to harness this new technology, medicine has been at the forefront of developing and discussing new ways to use GAI in research and its potential application patient care [3].

Medical education is another potential application for this technology [4]. ChatGPT has managed to pass the USMLE Step 1–3 exams and can generate its own exam questions for study support given proper prompting[5], suggesting its possible role in academic support. In terms of medical student support on clinical rotations, ChatGPT has been shown to produce high-quality clinical letters and suggest differential diagnoses and follow-up tests [6]. ChatGPT can edit manuscripts, cover letters and grant proposals and brainstorm research topics [7] ‐ all useful applications for medical students.

However, the ethical usage of ChatGPT in education must be considered. The technology is not perfect, often with inaccuracies, hallucinations, biases, and risk of plagiarism [8,9]. While there have been publications on ChatGPT usage in education [10,11], the opinions and current use of the technology by medical students in real-world settings has not been studied.

The objective of this study was to compile information about the use of ChatGPT and LLMs among medical students across North America. It seeks to understand the students’ awareness of ChatGPT, its current applications in medical schools, perceptions of its limitations, potential uses, and ethical considerations surrounding this popular technology through an open electronic survey.

## Methods

### Survey development

The study methodology was adapted from two surveys [12,13]. Two study members, (CG and ME), after an internal audit among the study authors, modified questions from the survey on ChatGPT in urology and developed novel questions using a two-round iterative process [14]. After each round, the current version of the survey was reviewed by GEC and edited to ensure clarity. A final version of the survey was tested by study authors to ensure proper functionality with the survey platform. Logical question structuring was applied to the survey to prevent respondents from answering questions that were not applicable based on previous answers.

The survey was divided into 7 parts (details reported in *S1 File)*:

Part 1 –Demographics

Part 2 –Awareness and Use of ChatGPT/LLMs in Medical School

Part 3 –ChatGPT/LLMs Use in Studying and Technology Integration

Part 4 –ChatGPT/LLMs Use in Clinical Rotations

Part 5 –ChatGPT/LLMs Use in Academic Writing Research

Part 6 –Ethical Concerns and Regulation of ChatGPT/LLMs

Part 7 –Respondent Contact

### Survey administration

Survey Monkey Plus (www.surveymokey.com) was used. This survey is a sample of convenience as it was open to all students pursuing a medical degree in the United States. The survey was deployed on May 4, 2023 for 14 days through social media, medical student group chats, and student collaborators. Many of these collaborators had established leadership roles at their respective institutions, most commonly as members of the Organization of Student Representatives (OSR), the official student liaisons to the AAMC. There were a variable number of items per page and 15 total pages of the questionnaire in total. Participants who completed the survey were required to answer all questions. The survey was voluntary, and there were no incentives offered for completing it. Participants were able to modify their answers at any point. The survey was terminated if the respondent had not heard of ChatGPT/LLM, which was asked after collecting demographic and background information. An additional deployment of the survey will be sent to responders who gave permission to be contacted at 12 month follow up.

### Ethics statement

This study was determined exempt and no more than minimal risk to human participants by the University of Southern California IRB (ID: UP-23-00888). Formal written consent, including clear instructions describing the aims of the survey and participation criteria, was obtained prior to the beginning of the survey.

### Data analysis and reporting

Data was analyzed on an anonymized basis and data access with identifiable information is limited to the authors of the study. Reported results refer to the number of complete responses, consisting of those who replied to all the survey questions or the relevant logic question within the survey. Descriptive data are reported as means and standard deviations for continuous variables, while numbers and percentages are used for categorical variables. Chi-squared testing was used for inferential analysis of categorical data. Charts and tables are used when appropriate to bolster the interpretability of the data. Data analysis was done using Tableau, JMP and SPSS. The data is reported following the Checklist for Reporting Results of Internet E-Surveys (CHERRIES) (*S2 File*) [15]. The first page of the survey provided basic information and an estimated completion time. Details about the questions and their responses are reported in the supplementary materials (*S3 File*).

## Results

### Demographic data

During the study period, 638 medical students initiated the survey questionnaire, of which 415 completed it (65.0% completion rate). Mean completion time was 9 minutes. Most respondents were female (61.7%) and between 25–34 years old (55.2%). The highest proportion of respondents were in their first year (M1: 35.4%), followed by third year (M3: 28.4%), second year (M2: 26.5%), and fourth year (M4: 9.2%). Regarding specialty interest, most (55.4%) were interested in internal medicine or medicine sub-specialties, while 35.9% were interested in surgery or surgical sub-specialties, 23.1% were interested in primary care, and 39.8% are interested in other specialties (pediatrics, EM, etc.). At least 28 medical schools were represented in the responses based on voluntary contact information provided. Demographic data is consolidated in Table 1.

**Table 1:** Demographic Data of Respondents.

Demographics	n %
**Gender**	
Male	154 (37)
Female	256 (62)
Non-binary	2 (0.5)
Prefer not to report	3 (0.7)
**Age (yrs)**	
< 18	0 (0)
18–24	183 (44)
25–34	229 (55)
35+	3 (1)
**Year of Training**	
First year (M1)	147 (35)
Second year (M2)	110 (26)
Third year (M3)	118 28
Forth year (M4)	38 (9)
**Specialty Interest**	
Primary care	96 (23)
Internal medicine	122 (29)
Internal medicine sub-specialty	108 (26)
General surgery	43 (10)
Surgical sub-specialty	106 (25.5)
Pediatrics	56 (13.5)
Other	100 (24)
**English As Primary Language**	
Yes	400 (96)
No	15 (4)

### Awareness and understanding of ChatGPT/LLMs

**Fig 1:**
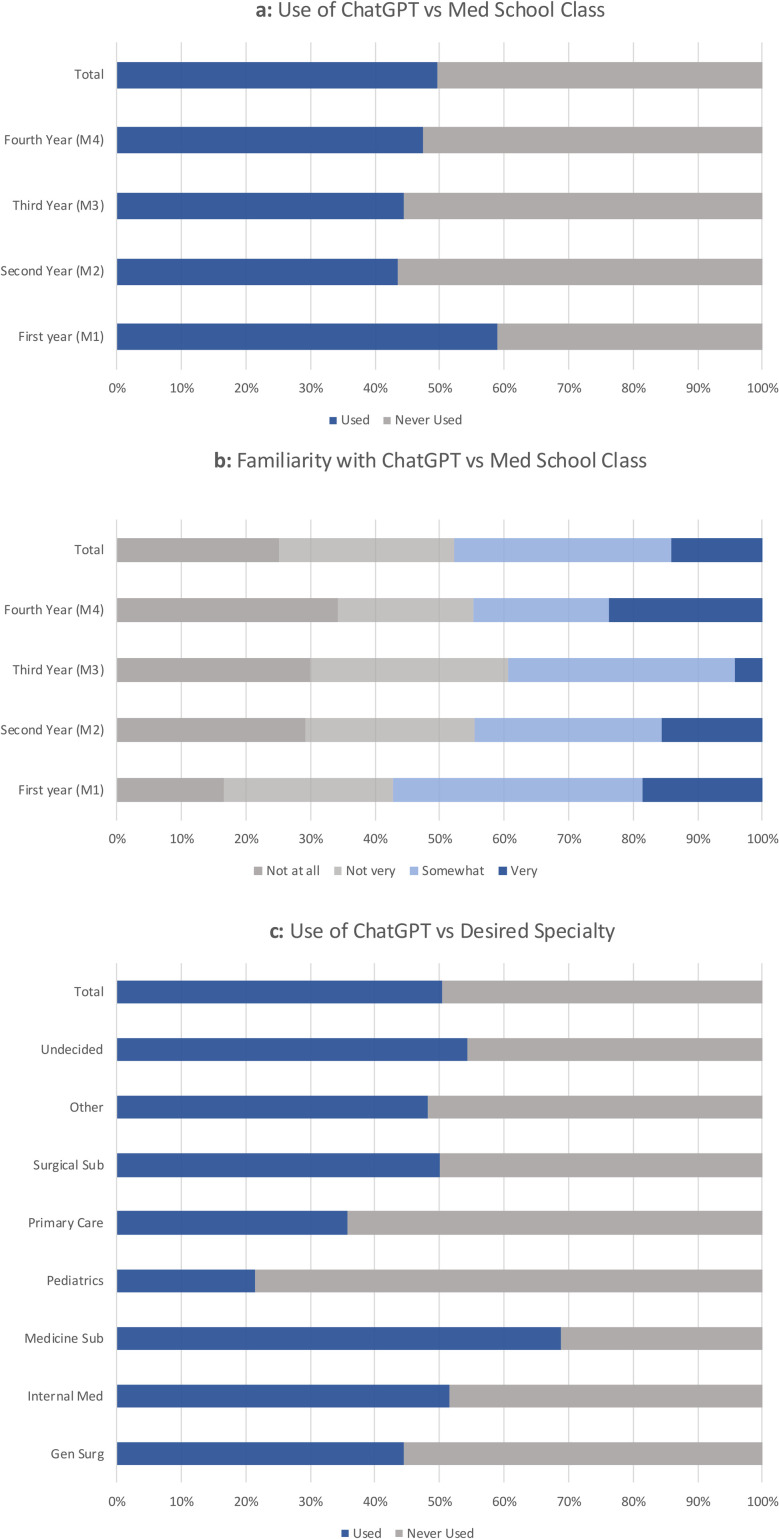
Respondent Use of and Familiarity with ChatGPT. The use and familiarity with ChatGPT among respondents was analyzed based on grade in medical school and desired specialty using Chi Squared statistical testing. P-values: 1a (p = 0.048), 1b (p = 0.003), 1c (p = 0.049).

Of those students participating, 69.7% were somewhat or very familiar with AI use in healthcare, while only 46.8% of participants were somewhat or very familiar with using AI in medical school. Using chi-squared analysis, there was a significant difference in use of ChatGPT based on school year, with M1s having the most user experience at 59.0% (p = 0.048) (Fig 1A). There was also a significant difference in familiarity with ChatGPT based on school year, with M1s being the most familiar and M3s being the least familiar (p = 0.003) (Fig 1B). Further, specialty interest also had a statistically significant impact on use of ChatGPT, with those interested primarily in medicine subspecialties most likely to use it (68.9%) while those desiring pediatrics (21.4%) and primary care (35.7%) were less likely to have used it (p = 0.049) (Fig 1C). Age was not statistically associated with use of (p = 0.44) or familiarity with (p = 0.56) ChatGPT.

Of the 415 participants, 17 (4.1%) were excluded from further analysis since they had no knowledge of or exposure to ChatGPT or other LLMs. Among those aware of ChatGPT, most were introduced to the technology via social media (39.5%) or friends/relatives (36.7%). Shortly after the release of ChatGPT in Nov 2022, 21.9% of respondents had been exposed to ChatGPT in some way. By February 2023, more than half of the students had been exposed to it. Of those who read scientific editorials on ChatGPT usage, 36.5% had an improved view of the technology while 49.0% did not have their view changed.

### Overall use in medical school

**Fig 2:**
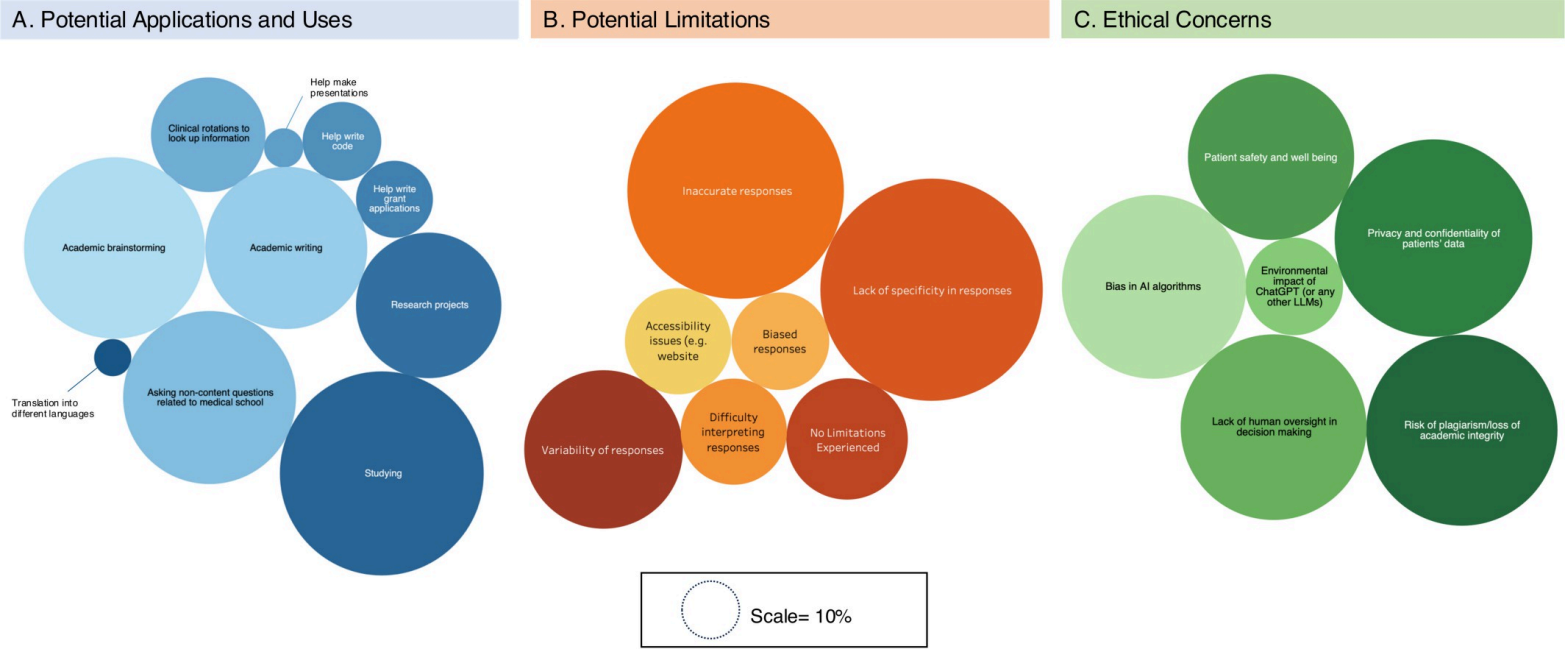
Potential Uses, Limitations, and Ethical Concerns Regarding ChatGPT. The size of the bubble refers to the percentage of respondents.

With regards to ChatGPT use in medical school, 52.0% have used ChatGPT specifically for med-school related tasks, while 61.3% overall have utilized the AI for a non-medical school purpose. Most (60.1%) affirmed ChatGPT’s usefulness in medical school, while a small minority (12.6%) did not think that LLMs could play an important role in their medical school experience. Among survey respondents, 38.4% report already having used it for studying, 29.4% for academic brainstorming, 26.4% for asking non-content questions related to medical school, and 23.4% used it for academic writing assistance. A more extensive list of uses is displayed in Fig 2A. When used for these tasks, 49.8% of respondents reported that using LLMs helped them save time.

### Use in studying and technology integration

Of the 38.4% of medical students that used it for studying, 100% asked it specific content questions directly to ChatGPT, 63.4% asked it to provide high yield information on a broad topic, 24.8% used it to summarize lecture outlines/slides, 24.2% asked it to create multiple choice practice questions, 20.9% used it to generate novel clinical case scenarios, and 9.8% used it to generate Anki© cards. Sixteen percent of respondents used ChatGPT for studying on a weekly basis, while 5.5% used ChatGPT in their daily studying routine.

Anki© was commonly used among respondents, with 86.2% reported using it and 66.5% using it every day. Of those who use Anki©, 4.7% have asked ChatGPT to generate Anki© cards and 13.4% used ChatGPT to elaborate and clarify difficult concepts encountered on other Anki© cards. Moreover, 53.0% of students would use ChatGPT to generate Anki© cards if there were tutorials available instructing people how to do so. When asked if ChatGPT could ever supplant Anki© as a medical education resource, 11.3% agreed while 50.8% disagreed.

Of the students using ChatGPT to generate multiple choice practice questions, 73.8% said they were at least somewhat useful. Further, if there were resources available instructing students on the optimal way to prompt ChatGPT to generate reliable questions, 55.0% of survey respondents would utilize them.

12.8% of respondents used ChatGPT to summarize school-provided lecture outlines/slide decks. Of these students, more than 90% found it at least somewhat useful. Of respondents who have attended lecture since the release of ChatGPT, 15.7% used ChatGPT to look up or clarify information during lecture. Further, 8.2% of students who attended lecture confirmed they knew of at least one instance of a professor/instructor using ChatGPT to augment their teaching materials. Of those teachers who used ChatGPT in the classroom, 25% used it to develop patient case scenarios and 18.8% used it to develop multiple choice questions.

### Use during clinical rotations

Among students who have completed a clinical rotation (42.2%) since the launch of ChatGPT, 22.5% have utilized ChatGPT during their clerkship. Of those who used ChatGPT on rotations, 73.7% prompted the chat bot for information regarding diagnosis and treatment options, 52.6% asked about pharmacology-related questions, and 26.3% used it to bolster follow-up care planning and patient education. Students utilized ChatGPT with varying frequency on rotations with 39.3% reporting weekly use and 10.5% reporting daily use. Students with recent clerkship experience believed that ChatGPT could be useful on rotations, with 39.3% reporting it could be somewhat useful and 18.5% reporting it was either very useful or extremely useful. In contrast, 42.3% did not view ChatGPT as useful during clinical rotations. Regarding the aspects of clinical that could benefit from the use of ChatGPT, 66.1% of students believe it could assist with writing notes, 59.5% think it could help with patient education, 44.6% think it could help guide treatment, and 38.7% think it can help with diagnosis. Regarding clinical notes, 4.8% report having used ChatGPT to help write or edit patient notes, with 75% using ChatGPT to craft their differential diagnosis based on patient signs/symptoms, 75% using it to outline sections of a write-up, and 62.50% using it to develop an assessment and plan.

Among respondents, 58.9% utilized paid resources like Amboss to look up clinical information on clinical rotations. Other commonly used resources include UpToDate (92.3%) and Anki (31.0%). If ChatGPT was demonstrated to be reliable and accurate, 57.7% of respondents would stop paying for resources like Amboss for accessing clinical information during clerkships. However, if ChatGPT was no longer free to access, 60.8% of all students indicated that they would not pay to continue using the tool while 26.9% were unsure.

### Research and academic writing

Among all survey respondents, 78.1% are involved in research during medical school and 27.4% have used ChatGPT during the research process. ChatGPT assisted the student researchers by: proof reading and editing grammar (59.6%), conducting literature reviews (35.8%), writing research articles or grant proposals (33.0%), generating study hypotheses (24.9%), managing citations (23.9%), analyzing data (8.3%), and translating text into different languages (1.8%). Most respondents (89.9%) indicated that ChatGPT improved their research productivity.

With regards to academic writing broadly, students utilized ChatGPT for outlining papers (45.9%), checking grammar (43.1%), summarizing text (42.20%), generating novel project ideas (41.3%), generating manuscript or essay text (32.1%), looking up citations (28.4%), and translating text into different languages (4.6%). Overall, 87.2% of respondents found ChatGPT at least somewhat effective at assisting academic writing. Further, 57.0% agree that ChatGPT will revolutionize the way scientific and academic writing is produced, while 14.3% disagree.

### Limitations of ChatGPT/LLMs

Of those who have used ChatGPT or other LLMs, 75.0% reported limitations when using the tools including: lack of specificity of responses (56.5%), inaccurate responses (53.4%), variability/inconsistency of responses (28.5%), unclear responses (12.7%), accessibility issues (12.7%), and biased responses (10.9%) (Fig 2B). These trends were similar when used specifically for studying (71.8% experienced limitations) and clinical rotations (57.4%), with a similar breakdown of specific limitations. When all respondents were asked about their level of trust in ChatGPT’s ability to provide reliable information, the majority (50.0%) reported average trust, followed by 29.9% reporting below average or far below average trust. Most respondents (56.9%) believe that ChatGPT should not be used in patient care currently given the lack of response validation by experts.

### Ethical considerations and regulation

When probed about potential ethical concerns that arise from the use of ChatGPT and LLMs in patient care and research, respondents were most concerned about patient data confidentiality when using the technology (87.4%) followed by plagiarism and risks to academic integrity (81.9%), lack of human oversite in decision making (77.4%), perpetuating biases through AI (75.9%), patient safety (61.8%), and environmental damage (21.4%) (Fig 2C). When asked about HIPAA compliance, 62.1% said that ChatGPT was not HIPAA compliant and input of patient identifiers into ChatGPT would constitute a HIPAA violation, while 32.2% were unsure about the HIPAA compliance of the technology. Regarding rules and regulations for the proper use and disclosure of ChatGPT, 91.7% said that clear regulations must be put in place and violators should be subject to penalties (60.8%). Regulations endorsed included ethical and legal oversight (82.2%), clear guidelines for use (81.7%), ensuring the AI is trained on accurate clinical guidelines (78.9%), and regular audits of AI systems (73.1%).

## Discussion

This prospective cross-sectional electronic survey provides a nation-wide snapshot of the medical student perspective on ChatGPT/LLMs. Most medical students are familiar with ChatGPT, and many who have utilized ChatGPT endorse its usefulness in medical school. Students recognize GAI’s potential in supporting studying, clinical rotations, and student research, but would like more resources on how to better integrate the technology with existing tools. While there is substantial use of this technology in medical school, students have observed limitations and recognize ethical issues and barriers of trust that need to be overcome before more widespread adoption. Students support regulation and guidelines on the use of ChatGPT/LLMs to improve trust and reduce the potential harms of this technology.

Respondent demographics demonstrated an accurate representation of American medical schools with regards to age and specialty interest [16]. Medical students earlier in their education were more likely to engage with the survey. Interestingly, M1s were more likely to be familiar with and use ChatGPT than upperclassmen. Junior medical students may be more open to new technologies since they have not cemented a medical school workflow. They also have more time to integrate this technology into their education.

Overall, 96% of survey respondents heard of ChatGPT, and a quarter of respondents were exposed to ChatGPT soon after it was released, demonstrating its viral nature. This is not unique to medical students, as ChatGPT set records as the fastest growing application [1]. Interestingly, more students reported using ChatGPT for non-medical than medical purposes. Perhaps students are aware of reported inaccuracies and “artificial hallucinations” in LLMs and are hesitant to use it for medical purposes [3,7]. Another possible explanation is a technology early adoption phase with limited awareness of how generative-AI can assist in medical education [17].

Over half of survey respondents have used ChatGPT for medical school-related tasks (ie, access to information, assistance in academic writing/editing), tasks reported in previous studies [18,19]. Interestingly, those interested in internal medicine subspecialties were more likely to use ChatGPT, demonstrating that this technology may not appeal equally to all medical students. Additionally, most thought ChatGPT could be useful in medical education, a sentiment echoed in other fields [20]. Students used ChatGPT to ask medical questions; utilization of readily accessible and easy to comprehend resources like Google and Wikipedia have been shown to be the most frequently used sites by medical students, even though content was rated less reliable compared to other resources [21]. Given ChatGPT’s user-friendly, chatbot format [11], students may similarly adopt this technology, as studies have shown ChatGPT to be superior to Google in relaying general medical knowledge [22]. In the future, respondents would be eager to use ChatGPT for other medical student specific tasks, such as developing practice multiple choice questions or Anki© cards, if there was instructional information on how to do so most effectively.

Conversations on how LLMs can be integrated into clinical practice medical education are ongoing [4,23]. In this survey, respondents agreed that ChatGPT could replace costly medical school study resources through an emphasis on personalized learning, but is less likely to replace specialized study tools like Anki©. Generative-AI technology is currently becoming integrated into existing technologies like Microsoft Office applications. LLMs have the potential to be optimized for industry specific tasks [24], we can perhaps imagine a future where it becomes integrated into pre-existing medical education and clinical platforms.

Survey results suggest ChatGPT is more positively viewed for non-clinical tasks (note writing, studying/summarizing class notes, editing grammar for research) than direct patient care. It is possible that students view using ChatGPT for studying as lower stakes compared to using it for clinical rotations, as there are likely still questions regarding its trust and credibility. Half of respondents agree that since outputs have not been validated, ChatGPT should not be use in a clinical care setting yet. Studies have already begun assessing the accuracy and reliability of using ChatGPT for a variety of academic and medical related tasks, such as performance on the United States Medical Licensing Exams [5], ability to write accurate patient letters [6], automate clinical notes [25], and disseminate health related inquiries [26]. Most respondents believe note writing and patient education could benefit from the use of ChatGPT if shown to be accurate and reliable, so more studies are needed.

When asked whether ChatGPT has improved research productivity, 90% responded affirmatively, and the majority believe it will revolutionize the way academic writing is done, and about one-quarter reported using it to generate hypothesis for research studies. ChatGPT has been shown to generate novel research topics [27], however it has also been shown to fabricate primary research citations [28]. Given that 25% of respondents have used the technology for citation management in their research, open conversations in both the classroom and research lab on the uses and limitations of ChatGPT is essential and has been proposed [29].

Despite the excitement, 75% of respondents experienced issues or limitations with using ChatGPT, and there remain questions regarding its ethical use. Medical students recognize bias in AI algorithms, privacy and confidentiality of patient’s data, risk of plagiarism/loss of academic integrity as potential issues. The majority believe stakeholders (university policies, etc.) should make regulations and rules for the proper use and disclosure of ChatGPT. There are current conversations and ongoing efforts to introduce guidelines for the ethical use of this technology in all facets of healthcare [30].

Survey respondents have already reported that professors and lectures have used ChatGPT to create content for their classes, and this trend is likely to increase as the technology becomes more widely accepted. This must be watched with caution, as ChatGPT is not trained on the most recent information and may provide biased, incomplete, or incorrect content that requires verification and oversight by experts [9].

Many students would not use ChatGPT if it was no longer free, a possibility mentioned by the founder of OpenAI [31]. As educational tools become increasingly digitized, issues of affordability and availability will require attention to ensure that all students can benefit. There are already overlooked causes of financial stress for medical students, such as board prep materials [32]. If GAI technology like ChatGPT become part of the “gold standard” for medical student learning, the adoption of these tools has the potential to increase financial stress and disparities for students if not offset by school funding.

### Limitations

The respondents to the survey were primarily recruited via social media and through student-led groups, which could potentially bias respondent selection towards those more proactive with new technologies. Further, this study was conducted with US medical students and results may not apply to medical schools in countries with different educational systems or limited access to generative-AI technology. The potential factors impacting medical students who were unaware of ChatGPT/LLMs was not investigated in this study, however these factors are likely to change rapidly given the rapid adoption and advancement of LLM models [1,33,34]. The impact of ChatGPT use on learning outcomes was not assessed given the recent adoption of this technology. A future study comparing academic metrics among those who utilize and do not utilize ChatGPT would be valuable for guiding medical education integration. This survey represents a snapshot of the current use of ChatGPT by medical students at six months after the release of ChatGPT. Therefore, a future deployment of the survey at twelve months following initial administration will monitor changes in usage of ChatGPT by medical students. The survey reflects the early adoption phase of this novel technology, and we suspect there will be changes in views and use as it develops further.

## Conclusion

In this prospective cross-sectional national survey, a significant portion of medical students are acquainted with the benefits of ChatGPT/LLMs in their academic and clinical journey. They not only recognize the potential of this technology in enhancing their studies, clinical rotations, and research but also express a keen interest in guidance for its seamless integration with existing tools. However, alongside its advantages, students are quick to point out certain limitations. Ethical dilemmas, trust barriers, and the need for a regulatory framework are among their primary concerns, suggesting a balanced perspective on the technology’s role in medical education.

## Supporting information

S1 File Survey section breakdown by question topic. (DOCX)

S2 File Checklist for Reporting Results of Internet E-Surveys (CHERRIES) completion for medical student survey. (DOCX)

S3 File Raw survey responses by medical students with percentage breakdowns included for each question. (PDF)
