# Supplementary material for: ChatGPT and large language models (LLMs) awareness and use. A prospective cross-sectional survey of U.S. medical students
Source: PLOS Digit Health. 2024 Sep 5;3(9):e0000596. doi: 10.1371/journal.pdig.0000596 (PMC11376538; doi:10.1371/journal.pdig.0000596)
Supplement: S1 File — (DOCX) [file pdig.0000596.s001.docx]

**S1 File:** Survey section breakdown by question topic.

*Part 1 – Demographics:* Collected basic demographic information on the respondents including their year, specialty interest, preferred language, and background knowledge/use of artificial intelligence historically.

*Part 2 – Awareness and Use of ChatGPT in Medical School:* Explored the awareness and perceptions of of ChatGPT/LLMs among respondents, including uses and applications broadly during medical school.

*Part 3 – ChatGPT Use in Studying and Technology Integration:* Assessed current and potential uses of ChatGPT for studying in medical school, as well as incorporation with existing technology and learning resources.

*Part 4 – ChatGPT Use in Clinical Rotations:* Investigated use cases for ChatGPT on clinical rotations, including both information gathering and text generation.

*Part 5 – ChatGPT Use in Academic Writing Resaerch:* Looked at how respondents were using and could use ChatGPT for streamlining academic writing and research.

*Part 6 – Ethical Concerns and Regulation of ChatGPT:* Addressed ethical concerns related to the use of ChatGPT in medical school and medicine broadly. Evaluated the desire for regulation and safeguards around the use of this new technology.

*Part 7 - Respondent Contact:* Requested participant contact information to follow up on their usage of ChatGPT and invite them to follow-up surveys.
