# Supplementary material for: ChatGPT and large language models (LLMs) awareness and use. A prospective cross-sectional survey of U.S. medical students
Source: PLOS Digit Health. 2024 Sep 5;3(9):e0000596. doi: 10.1371/journal.pdig.0000596 (PMC11376538; doi:10.1371/journal.pdig.0000596)
Supplement: S3 File — (PDF) [file pdig.0000596.s003.pdf]

Q1 Are you a medical student (candidate for MD, DO, MBBS, MB, MBCHB, ect.)?

Answered: 415 Skipped: 0

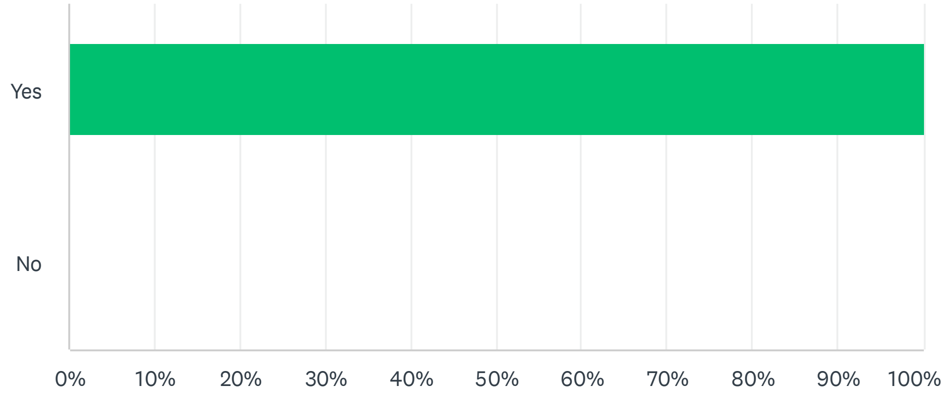

ANSWER CHOICES	RESPONSES	
Yes	100.00%	415
No	0.00%	0
TOTAL		415

Q2 What is your age

Answered: 415 Skipped: 0

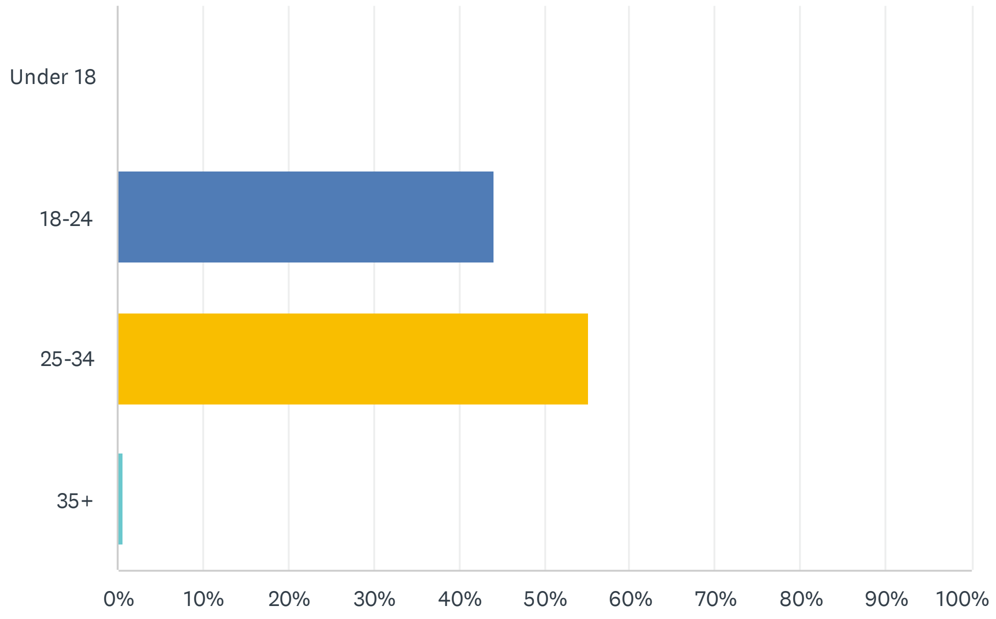

ANSWER CHOICES	RESPONSES	
Under 18	0.00%	0
18-24	44.10%	183
25-34	55.18%	229
35+	0.72%	3
TOTAL		415

Q3 What is your gender?

Answered: 415 Skipped: 0

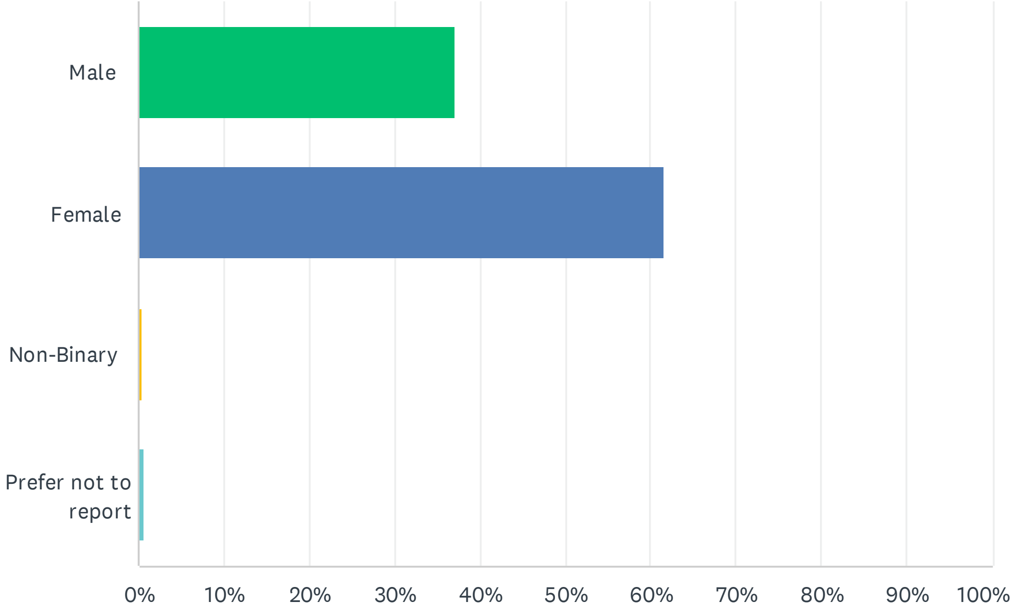

ANSWER CHOICES	RESPONSES	
Male	37.11%	154
Female	61.69%	256
Non-Binary	0.48%	2
Prefer not to report	0.72%	3
TOTAL		415

Q4 What is your location of medical school?

Answered: 415 Skipped: 0

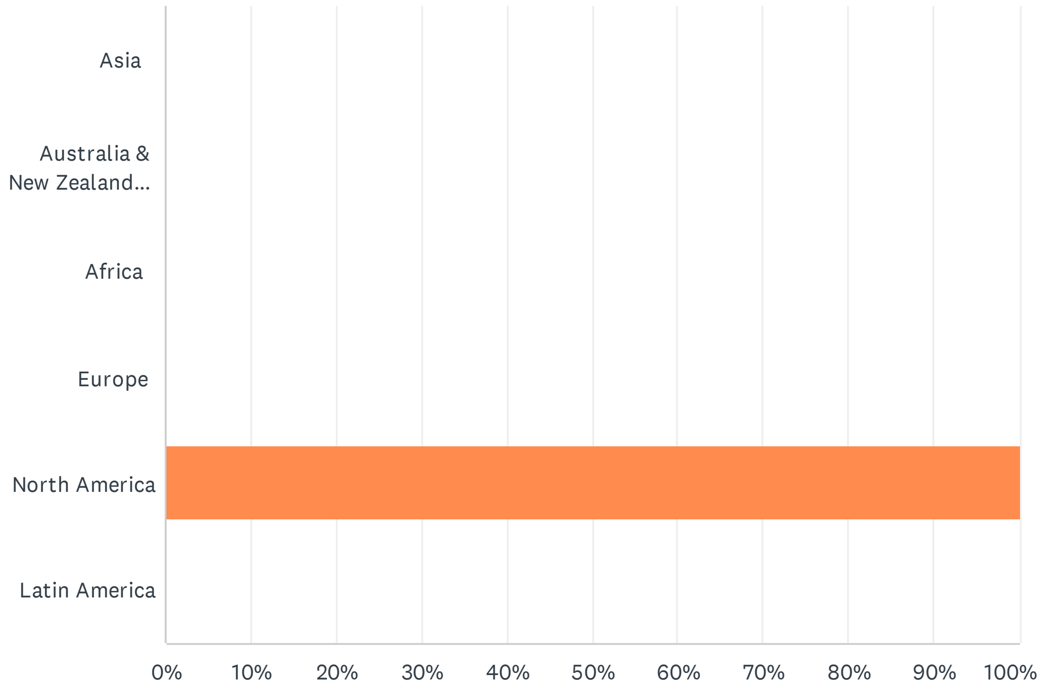

ANSWER CHOICES	RESPONSES	
Asia	0.00%	0
Australia & New Zealand (Oceania)	0.00%	0
Africa	0.00%	0
Europe	0.00%	0
North America	100.00%	415
Latin America	0.00%	0
TOTAL		415

Q5 Please specify the country that you are currently studying in

Answered: 415 Skipped: 0

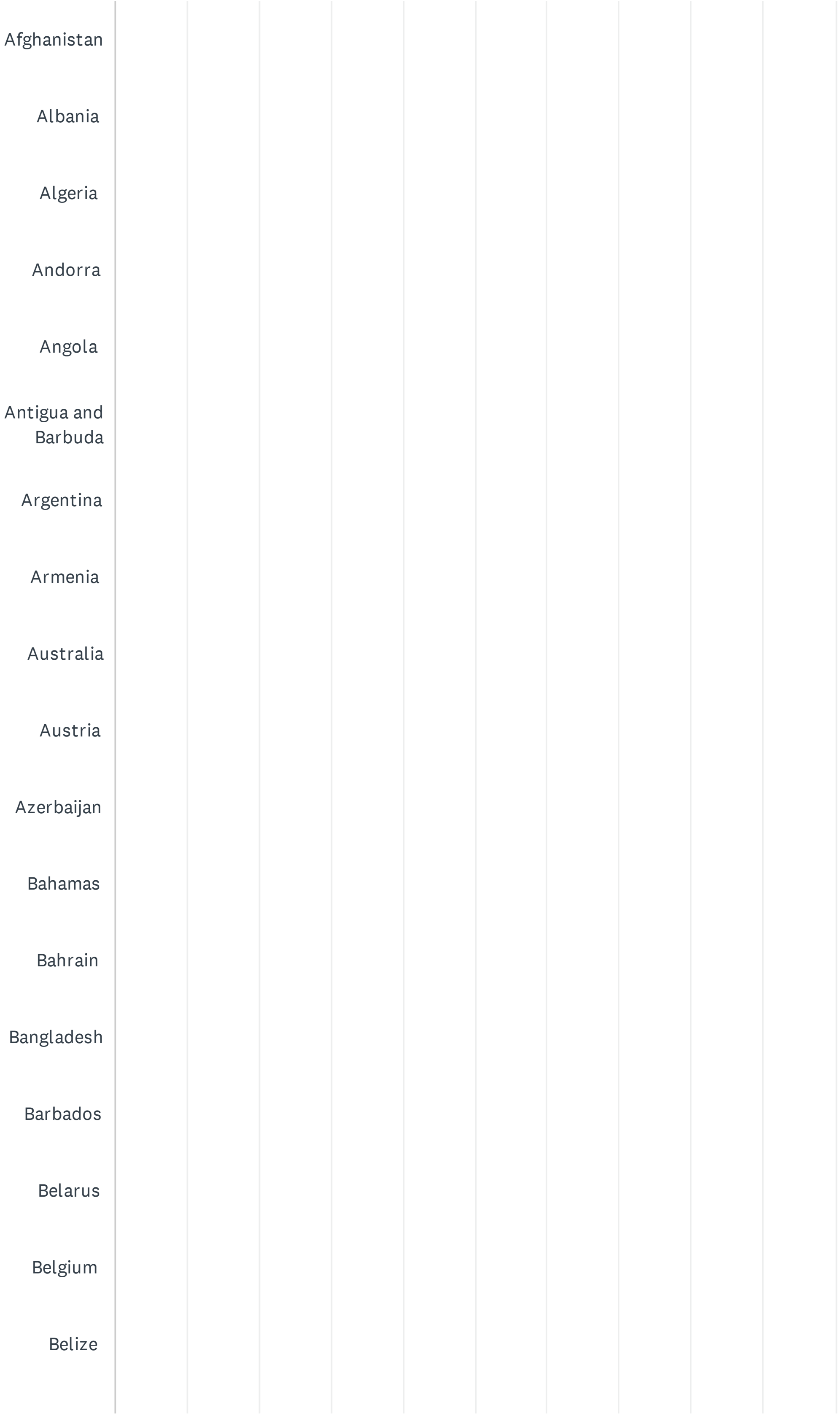

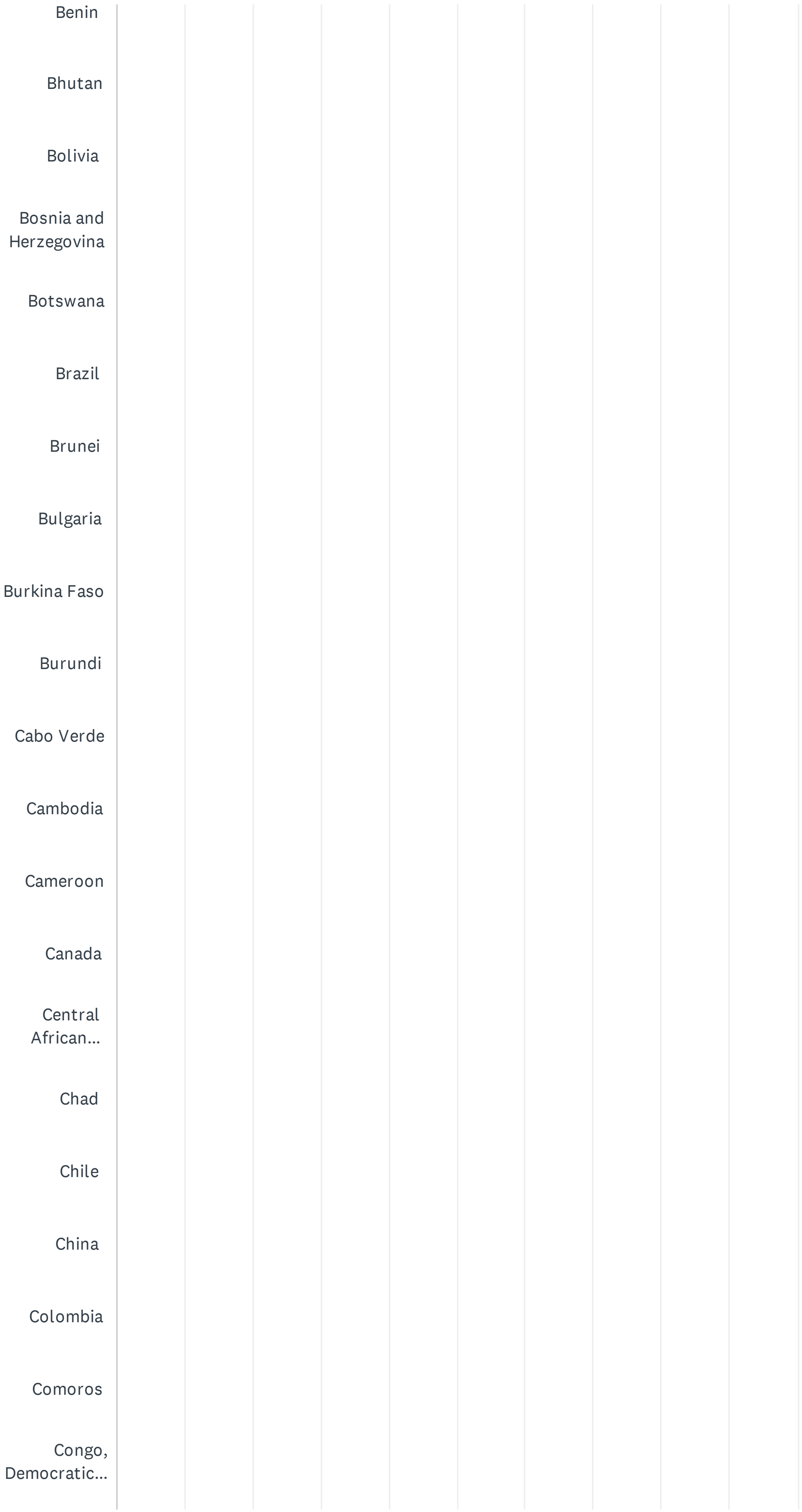
Benin
Bhutan
Bolivia
Bosnia and Herzegovina
Botswana
Brazil
Brunei
Bulgaria
Burkina Faso
Burundi
Cabo Verde
Cambodia
Cameroon
Canada
Central African...
Chad
Chile
China
Colombia
Comoros
Congo, Democratic...

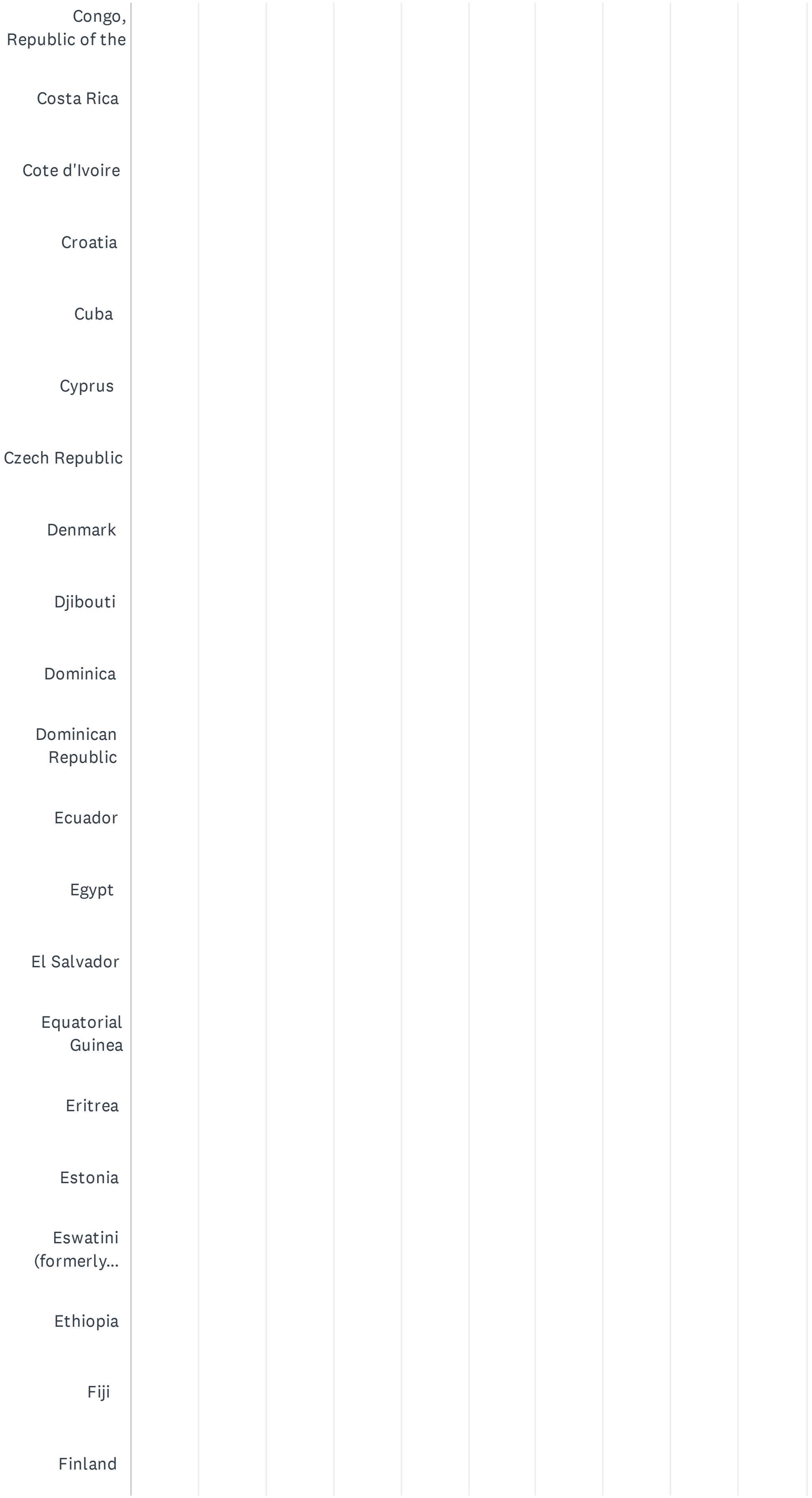
Congo, Republic of the
Costa Rica
Cote d'Ivoire
Croatia
Cuba
Cyprus
Czech Republic
Denmark
Djibouti
Dominica
Dominican Republic
Ecuador
Egypt
El Salvador
Equatorial Guinea
Eritrea
Estonia
Eswatini (formerly...
Ethiopia
Fiji
Finland

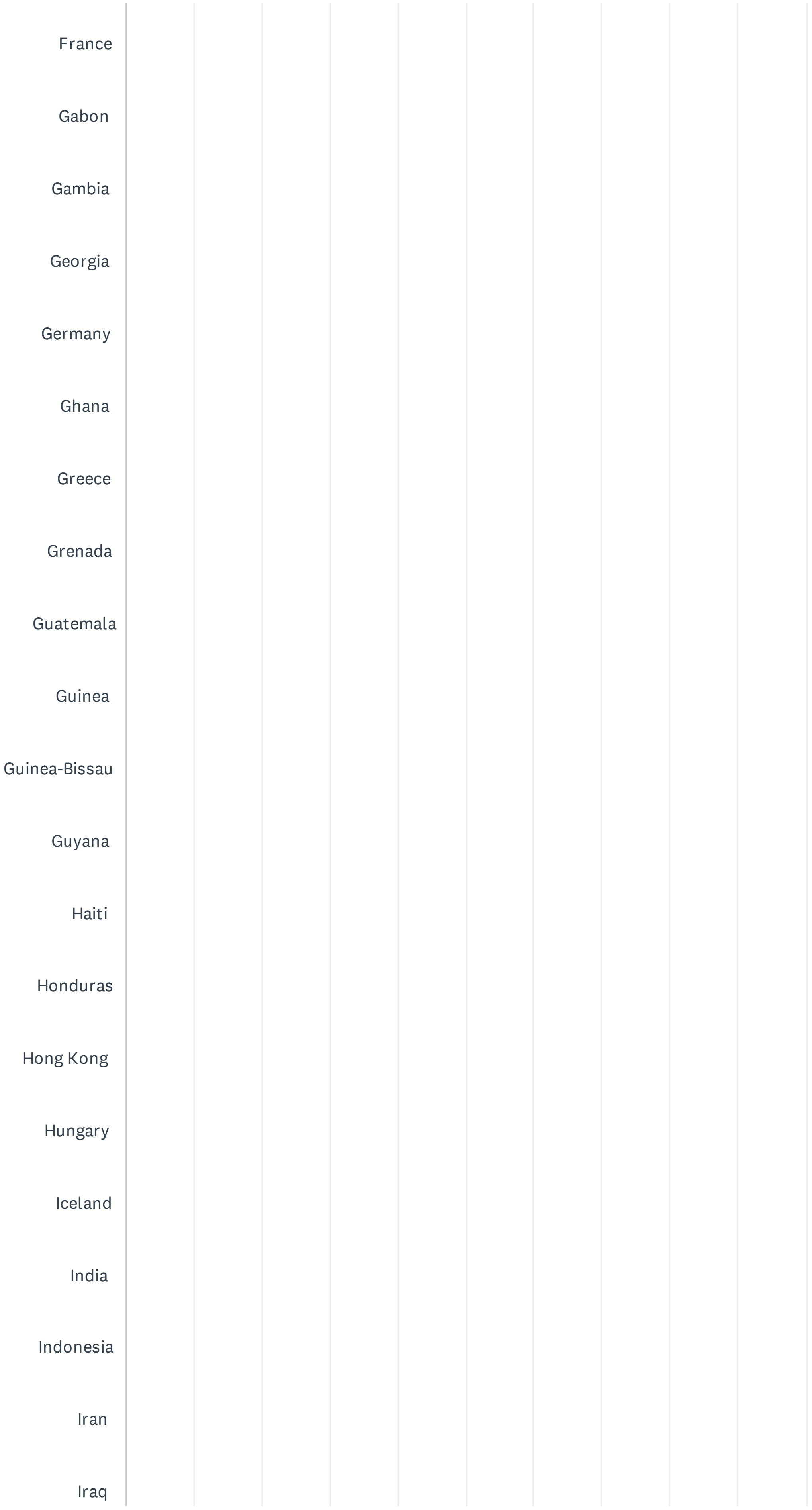
France
Gabon
Gambia
Georgia
Germany
Ghana
Greece
Grenada
Guatemala
Guinea
Guinea-Bissau
Guyana
Haiti
Honduras
Hong Kong
Hungary
Iceland
India
Indonesia
Iran
Iraq

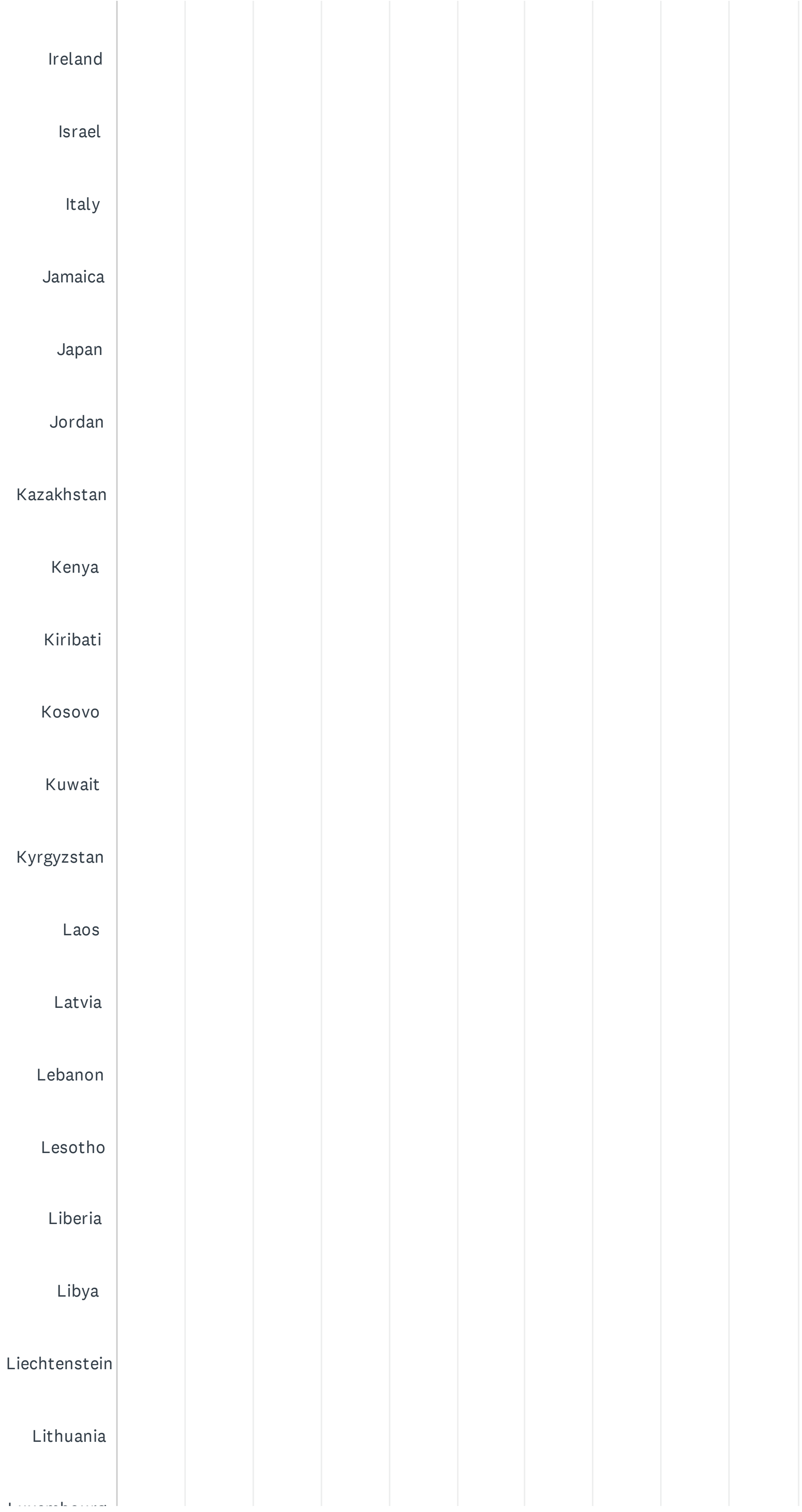
Ireland
Israel
Italy
Jamaica
Japan
Jordan
Kazakhstan
Kenya
Kiribati
Kosovo
Kuwait
Kyrgyzstan
Laos
Latvia
Lebanon
Lesotho
Liberia
Libya
Liechtenstein
Lithuania

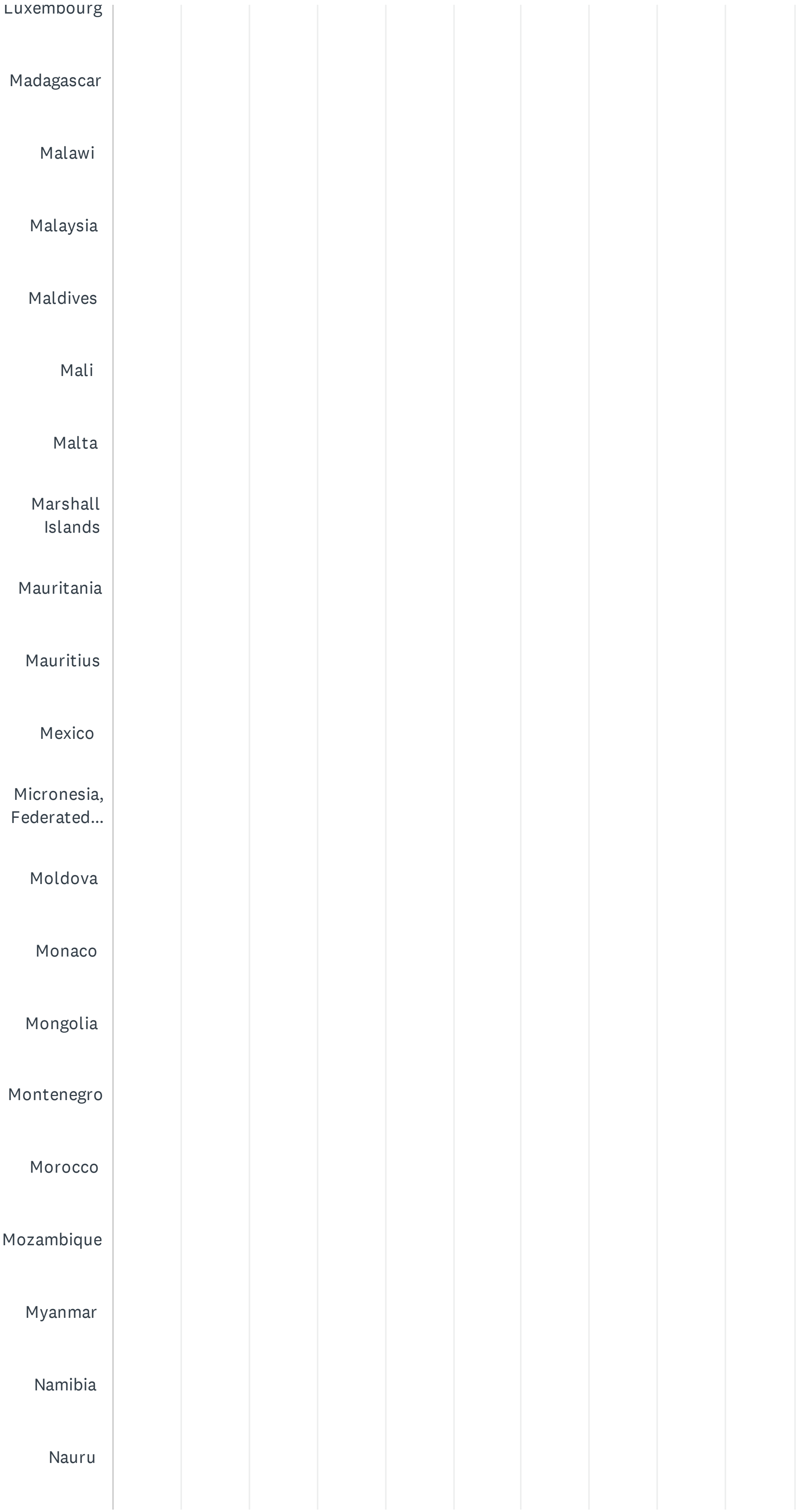
Luxembourg
Madagascar
Malawi
Malaysia
Maldives
Mali
Malta
Marshall Islands
Mauritania
Mauritius
Mexico
Micronesia, Federated...
Moldova
Monaco
Mongolia
Montenegro
Morocco
Mozambique
Myanmar
Namibia
Nauru

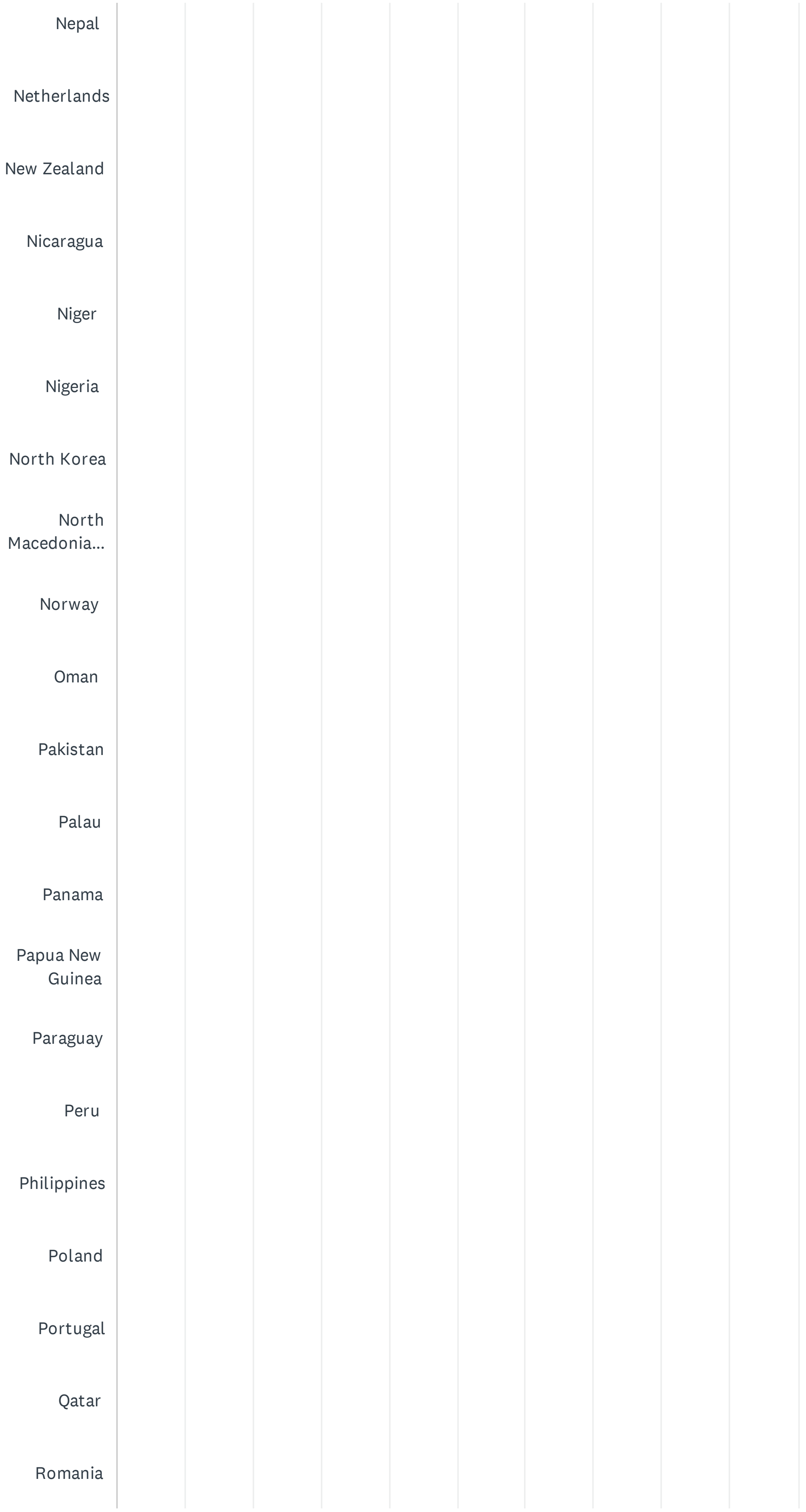
Nepal
Netherlands
New Zealand
Nicaragua
Niger
Nigeria
North Korea
North Macedonia...
Norway
Oman
Pakistan
Palau
Panama
Papua New Guinea
Paraguay
Peru
Philippines
Poland
Portugal
Qatar
Romania

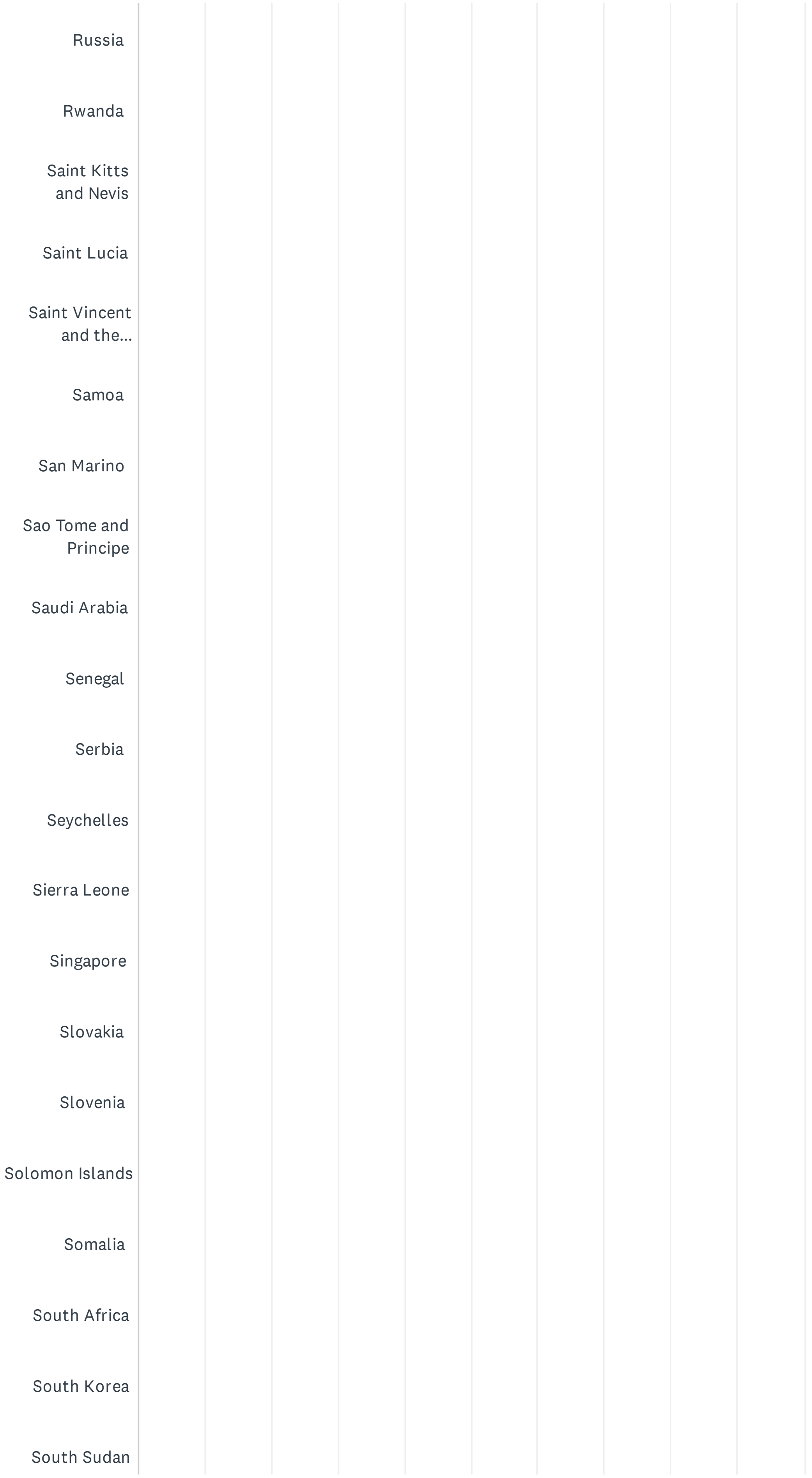
Russia
Rwanda
Saint Kitts and Nevis
Saint Lucia
Saint Vincent and the...
Samoa
San Marino
Sao Tome and Principe
Saudi Arabia
Senegal
Serbia
Seychelles
Sierra Leone
Singapore
Slovakia
Slovenia
Solomon Islands
Somalia
South Africa
South Korea
South Sudan

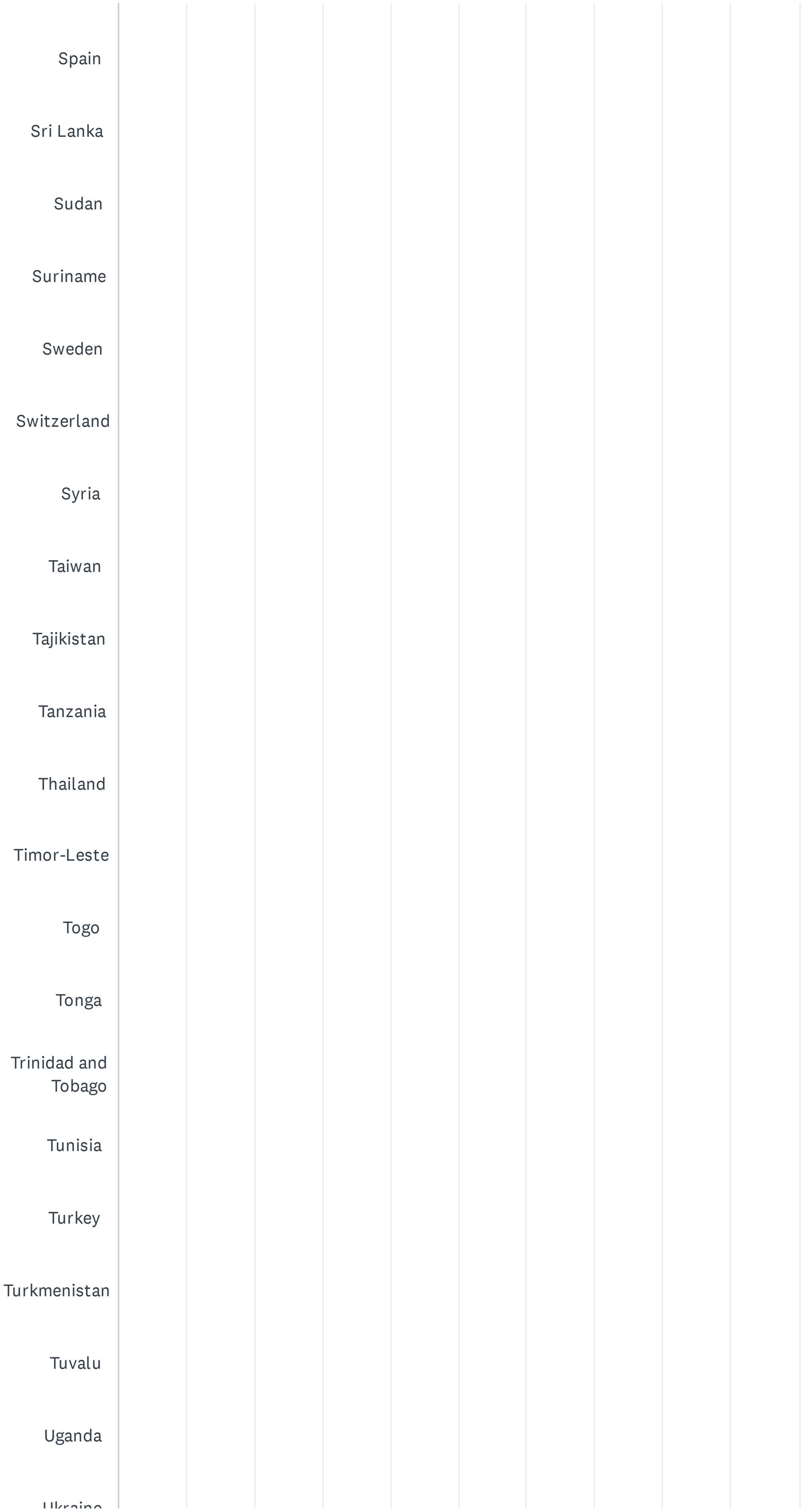
Spain
Sri Lanka
Sudan
Suriname
Sweden
Switzerland
Syria
Taiwan
Tajikistan
Tanzania
Thailand
Timor-Leste
Togo
Tonga
Trinidad and Tobago
Tunisia
Turkey
Turkmenistan
Tuvalu
Uganda

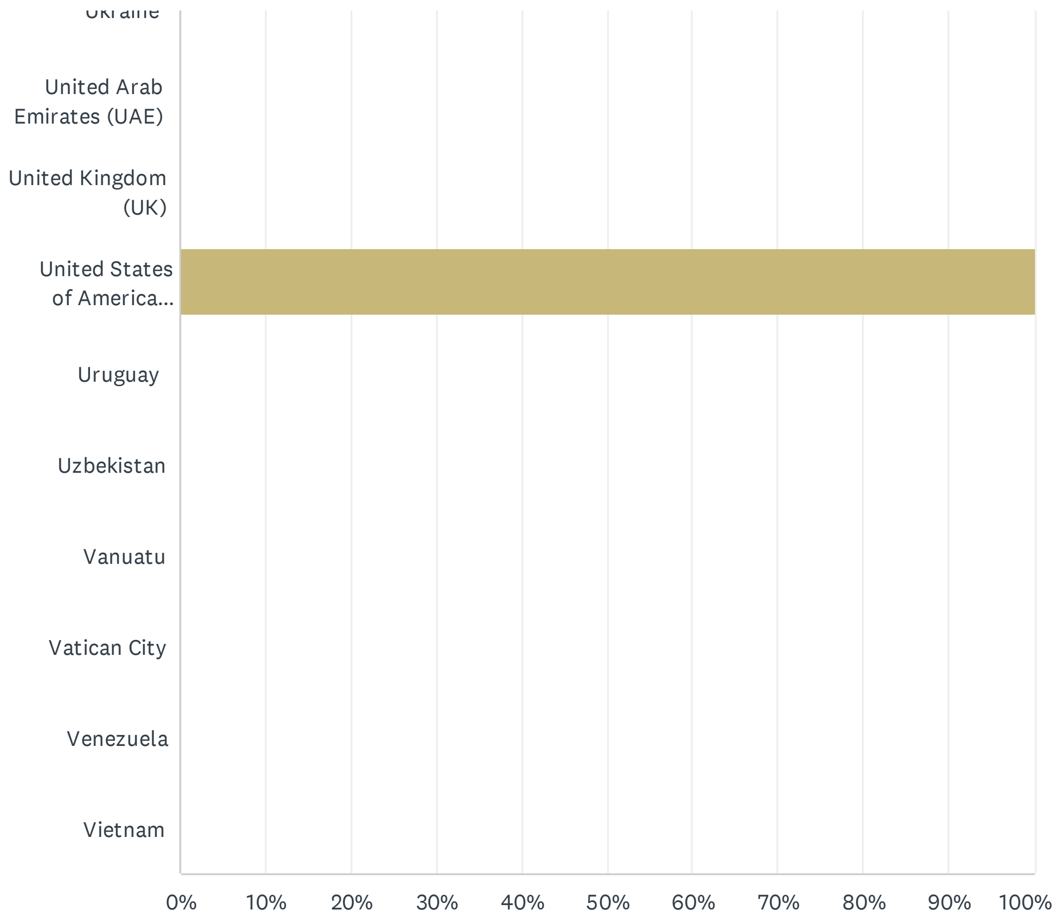
Ukraine
United Arab Emirates (UAE)
United Kingdom (UK)
United States of America...
Uruguay
Uzbekistan
Vanuatu
Vatican City
Venezuela
Vietnam
0%
10%
20%
30%
40%
50%
60%
70%
80%
90%
100%

ANSWER CHOICES	RESPONSES	
Afghanistan	0.00%	0
Albania	0.00%	0
Algeria	0.00%	0
Andorra	0.00%	0
Angola	0.00%	0
Antigua and Barbuda	0.00%	0
Argentina	0.00%	0
Armenia	0.00%	0
Australia	0.00%	0
Austria	0.00%	0
Azerbaijan	0.00%	0
Bahamas	0.00%	0
Bahrain	0.00%	0
Bangladesh	0.00%	0
Barbados	0.00%	0
Belarus	0.00%	0
Belgium	0.00%	0
Belize	0.00%	0
Benin	0.00%	0
Bhutan	0.00%	0
Bolivia	0.00%	0
Bosnia and Herzegovina	0.00%	0
Botswana	0.00%	0
Brazil	0.00%	0
Brunei	0.00%	0
Bulgaria	0.00%	0
Burkina Faso	0.00%	0
Burundi	0.00%	0
Cabo Verde	0.00%	0
Cambodia	0.00%	0
Cameroon	0.00%	0
Canada	0.00%	0

Central African Republic	0.00%	0
Chad	0.00%	0
Chile	0.00%	0
China	0.00%	0
Colombia	0.00%	0
Comoros	0.00%	0
Congo, Democratic Republic of the	0.00%	0
Congo, Republic of the	0.00%	0
Costa Rica	0.00%	0
Cote d'Ivoire	0.00%	0
Croatia	0.00%	0
Cuba	0.00%	0
Cyprus	0.00%	0
Czech Republic	0.00%	0
Denmark	0.00%	0
Djibouti	0.00%	0
Dominica	0.00%	0
Dominican Republic	0.00%	0
Ecuador	0.00%	0
Egypt	0.00%	0
El Salvador	0.00%	0
Equatorial Guinea	0.00%	0
Eritrea	0.00%	0
Estonia	0.00%	0
Eswatini (formerly Swaziland)	0.00%	0
Ethiopia	0.00%	0
Fiji	0.00%	0
Finland	0.00%	0
France	0.00%	0
Gabon	0.00%	0
Gambia	0.00%	0
Georgia	0.00%	0
Germany	0.00%	0
Ghana	0.00%	0

Greece	0.00%	0
Grenada	0.00%	0
Guatemala	0.00%	0
Guinea	0.00%	0
Guinea-Bissau	0.00%	0
Guyana	0.00%	0
Haiti	0.00%	0
Honduras	0.00%	0
Hong Kong	0.00%	0
Hungary	0.00%	0
Iceland	0.00%	0
India	0.00%	0
Indonesia	0.00%	0
Iran	0.00%	0
Iraq	0.00%	0
Ireland	0.00%	0
Israel	0.00%	0
Italy	0.00%	0
Jamaica	0.00%	0
Japan	0.00%	0
Jordan	0.00%	0
Kazakhstan	0.00%	0
Kenya	0.00%	0
Kiribati	0.00%	0
Kosovo	0.00%	0
Kuwait	0.00%	0
Kyrgyzstan	0.00%	0
Laos	0.00%	0
Latvia	0.00%	0
Lebanon	0.00%	0
Lesotho	0.00%	0
Liberia	0.00%	0

Libya	0.00%	0
Liechtenstein	0.00%	0
Lithuania	0.00%	0
Luxembourg	0.00%	0
Madagascar	0.00%	0
Malawi	0.00%	0
Malaysia	0.00%	0
Maldives	0.00%	0
Mali	0.00%	0
Malta	0.00%	0
Marshall Islands	0.00%	0
Mauritania	0.00%	0
Mauritius	0.00%	0
Mexico	0.00%	0
Micronesia, Federated States of	0.00%	0
Moldova	0.00%	0
Monaco	0.00%	0
Mongolia	0.00%	0
Montenegro	0.00%	0
Morocco	0.00%	0
Mozambique	0.00%	0
Myanmar	0.00%	0
Namibia	0.00%	0
Nauru	0.00%	0
Nepal	0.00%	0
Netherlands	0.00%	0
New Zealand	0.00%	0
Nicaragua	0.00%	0
Niger	0.00%	0
Nigeria	0.00%	0
North Korea	0.00%	0
North Macedonia (formerly Macedonia)	0.00%	0
Norway	0.00%	0
Oman	0.00%	0

Pakistan	0.00%	0
Palau	0.00%	0
Panama	0.00%	0
Papua New Guinea	0.00%	0
Paraguay	0.00%	0
Peru	0.00%	0
Philippines	0.00%	0
Poland	0.00%	0
Portugal	0.00%	0
Qatar	0.00%	0
Romania	0.00%	0
Russia	0.00%	0
Rwanda	0.00%	0
Saint Kitts and Nevis	0.00%	0
Saint Lucia	0.00%	0
Saint Vincent and the Grenadines	0.00%	0
Samoa	0.00%	0
San Marino	0.00%	0
Sao Tome and Principe	0.00%	0
Saudi Arabia	0.00%	0
Senegal	0.00%	0
Serbia	0.00%	0
Seychelles	0.00%	0
Sierra Leone	0.00%	0
Singapore	0.00%	0
Slovakia	0.00%	0
Slovenia	0.00%	0
Solomon Islands	0.00%	0
Somalia	0.00%	0
South Africa	0.00%	0
South Korea	0.00%	0
South Sudan	0.00%	0
Spain	0.00%	0

Sri Lanka	0.00%	0
Sudan	0.00%	0
Suriname	0.00%	0
Sweden	0.00%	0
Switzerland	0.00%	0
Syria	0.00%	0
Taiwan	0.00%	0
Tajikistan	0.00%	0
Tanzania	0.00%	0
Thailand	0.00%	0
Timor-Leste	0.00%	0
Togo	0.00%	0
Tonga	0.00%	0
Trinidad and Tobago	0.00%	0
Tunisia	0.00%	0
Turkey	0.00%	0
Turkmenistan	0.00%	0
Tuvalu	0.00%	0
Uganda	0.00%	0
Ukraine	0.00%	0
United Arab Emirates (UAE)	0.00%	0
United Kingdom (UK)	0.00%	0
United States of America (USA)	100.00%	415
Uruguay	0.00%	0
Uzbekistan	0.00%	0
Vanuatu	0.00%	0
Vatican City	0.00%	0
Venezuela	0.00%	0
Vietnam	0.00%	0
TOTAL		415

Q6 What year of training are you in? (if you are taking time off for an additional degree or research, indicate prior year).

Answered: 415 Skipped: 0

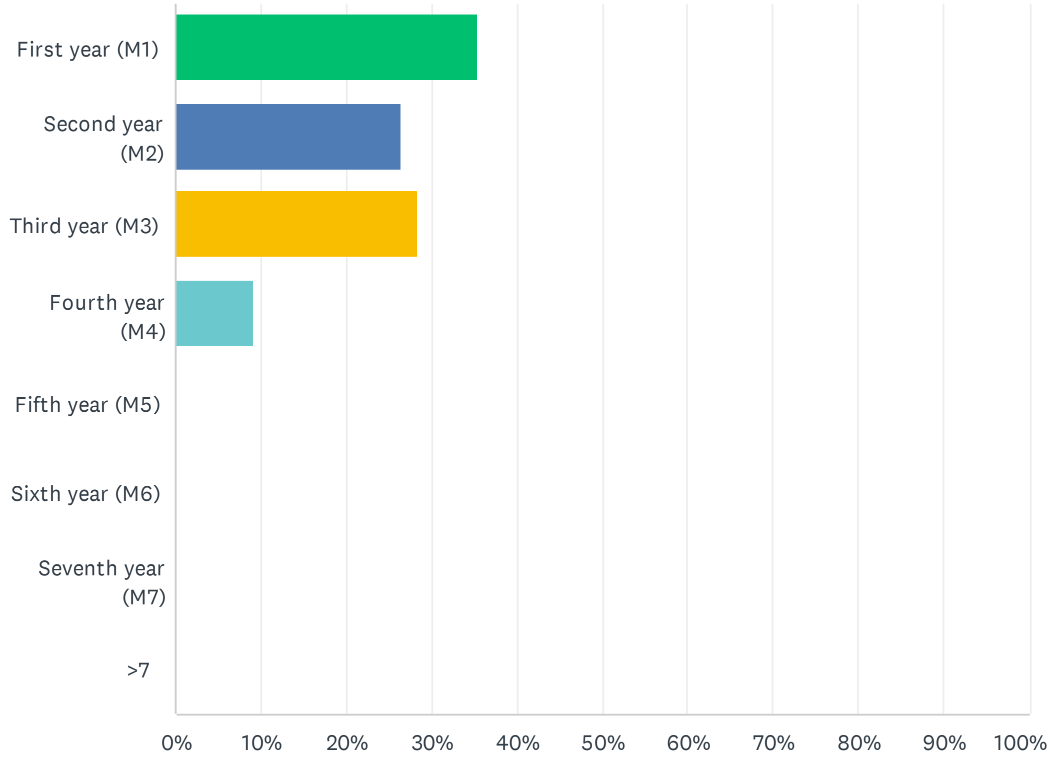

ANSWER CHOICES	RESPONSES	
First year (M1)	35.42%	147
Second year (M2)	26.51%	110
Third year (M3)	28.43%	118
Fourth year (M4)	9.16%	38
Fifth year (M5)	0.24%	1
Sixth year (M6)	0.24%	1
Seventh year (M7)	0.00%	0
>7	0.00%	0
TOTAL		415

Q7 What is your primary area of interest/goal for residency?

Answered: 415 Skipped: 0

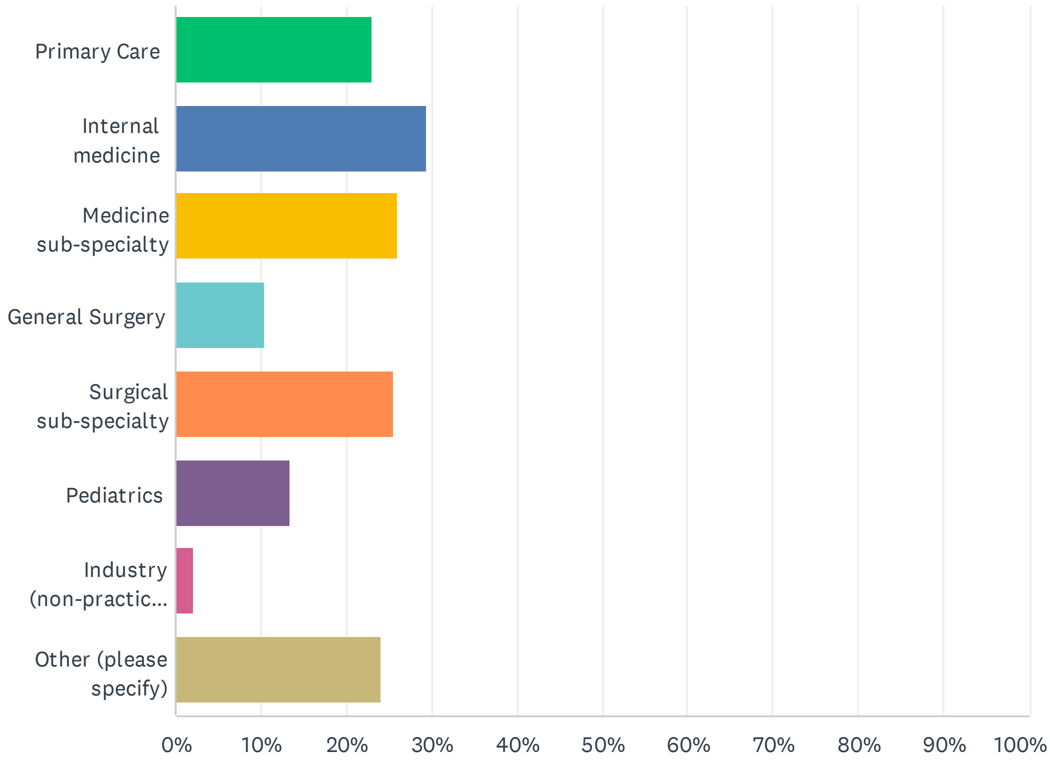

ANSWER CHOICES	RESPONSES	
Primary Care	23.13%	96
Internal medicine	29.40%	122
Medicine sub-specialty	26.02%	108
General Surgery	10.36%	43
Surgical sub-specialty	25.54%	106
Pediatrics	13.49%	56
Industry (non-practicing physician)	2.17%	9
Other (please specify)	24.10%	100
Total Respondents: 415		

Q8 Is English your primary language?

Answered: 415 Skipped: 0

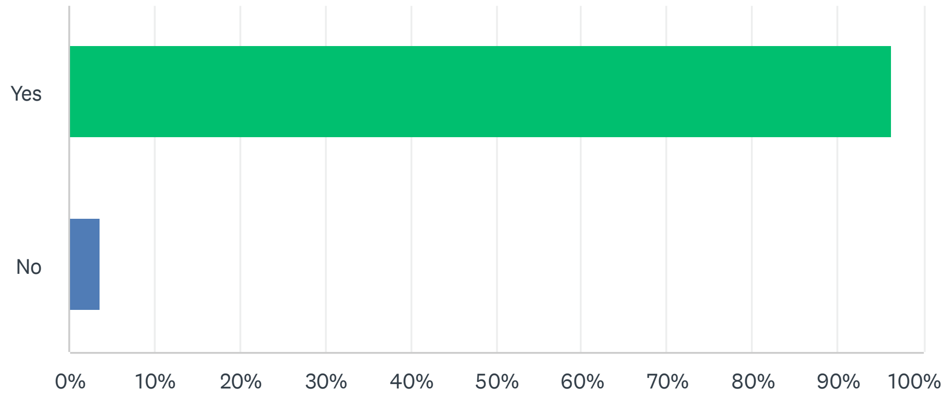

ANSWER CHOICES	RESPONSES	
Yes	96.39%	400
No	3.61%	15
TOTAL		415

Q9 If English is not your primary language, are you at least proficient in English

Answered: 415 Skipped: 0

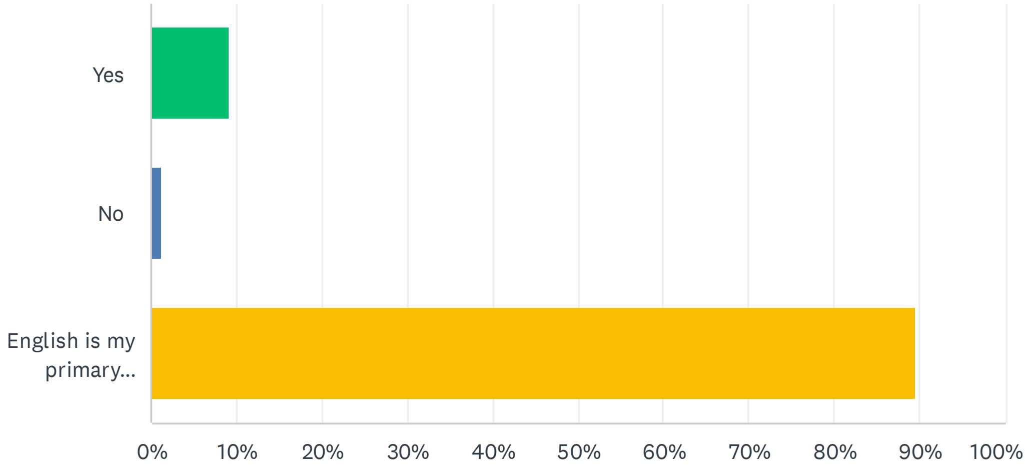

ANSWER CHOICES	RESPONSES	
Yes	9.16%	38
No	1.20%	5
English is my primary language	89.64%	372
TOTAL		415

Q10 Primary language

Answered: 415 Skipped: 0

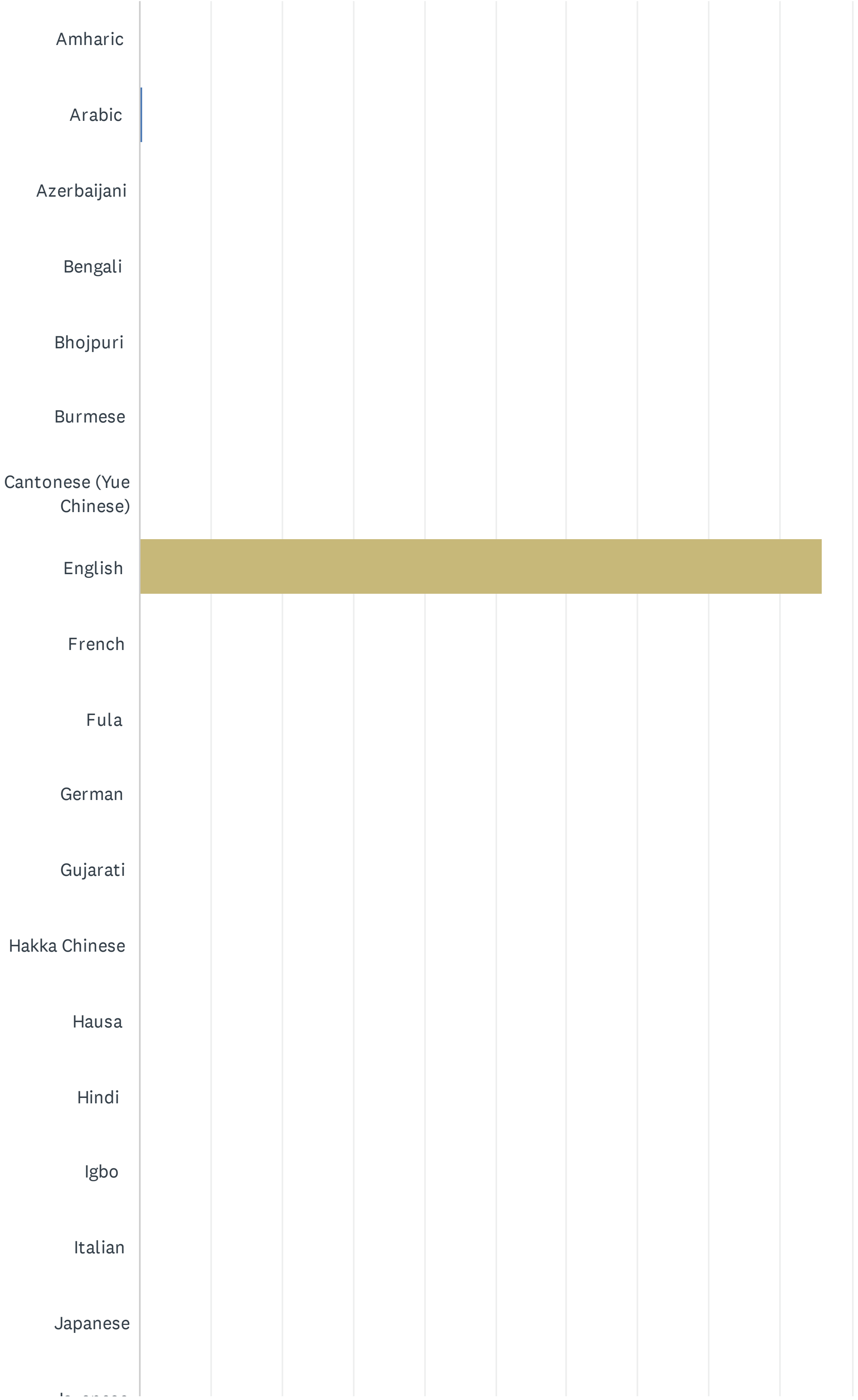

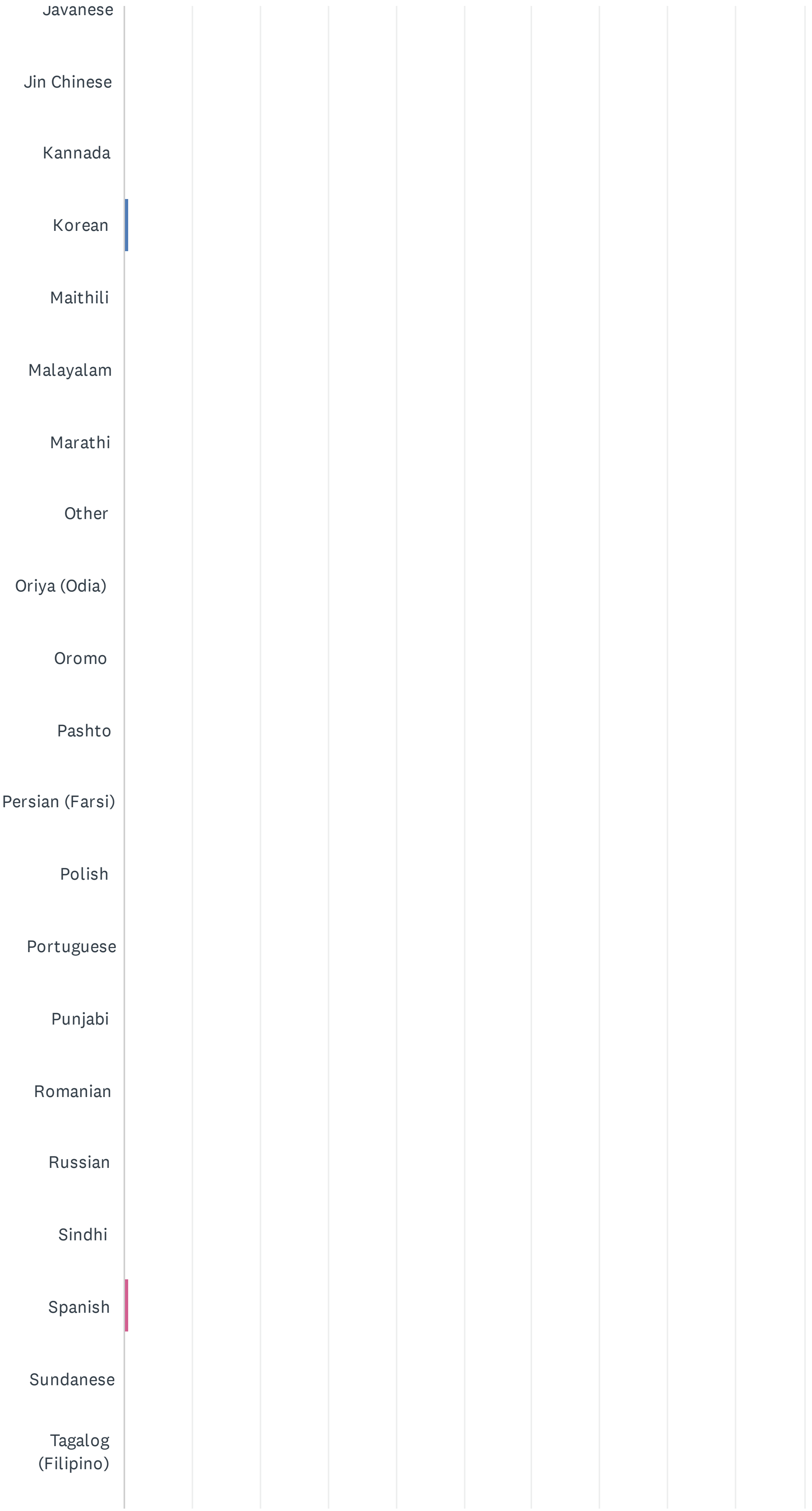
Javanese
Jin Chinese
Kannada
Korean
Maithili
Malayalam
Marathi
Other
Oriya (Odia)
Oromo
Pashto
Persian (Farsi)
Polish
Portuguese
Punjabi
Romanian
Russian
Sindhi
Spanish
Sundanese
Tagalog (Filipino)

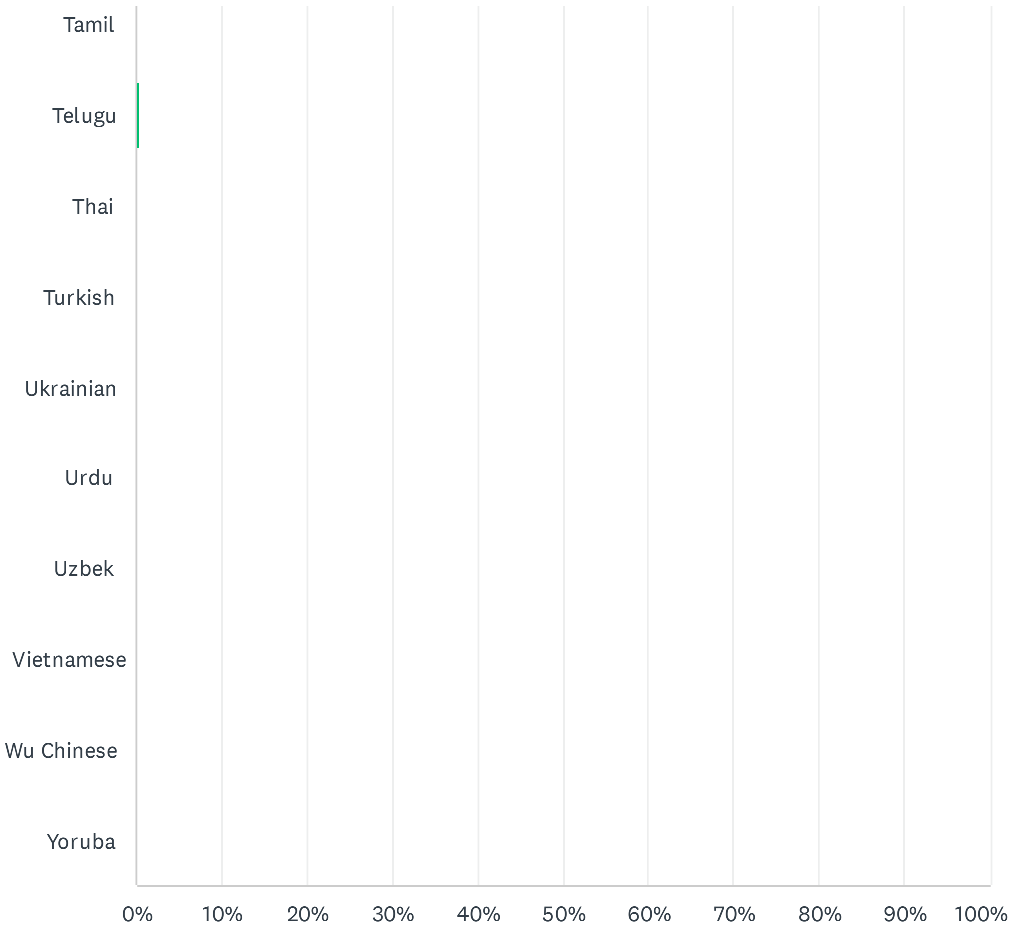
Tamil
Telugu
Thai
Turkish
Ukrainian
Urdu
Uzbek
Vietnamese
Wu Chinese
Yoruba
0%
10%
20%
30%
40%
50%
60%
70%
80%
90%
100%

ANSWER CHOICES	RESPONSES	
Amharic	0.00%	0
Arabic	0.48%	2
Azerbaijani	0.00%	0
Bengali	0.00%	0
Bhojpuri	0.00%	0
Burmese	0.00%	0
Cantonese (Yue Chinese)	0.24%	1
English	95.90%	398
French	0.24%	1
Fula	0.00%	0
German	0.00%	0
Gujarati	0.24%	1
Hakka Chinese	0.00%	0
Hausa	0.00%	0
Hindi	0.00%	0
Igbo	0.00%	0
Italian	0.00%	0
Japanese	0.00%	0
Javanese	0.00%	0
Jin Chinese	0.24%	1
Kannada	0.00%	0
Korean	0.72%	3
Maithili	0.00%	0
Malayalam	0.00%	0
Marathi	0.24%	1
Other	0.24%	1
Oriya (Odia)	0.00%	0
Oromo	0.00%	0
Pashto	0.00%	0
Persian (Farsi)	0.24%	1
Polish	0.00%	0
Portuguese	0.00%	0

Punjabi	0.00%	0
Romanian	0.00%	0
Russian	0.00%	0
Sindhi	0.00%	0
Spanish	0.72%	3
Sundanese	0.00%	0
Tagalog (Filipino)	0.00%	0
Tamil	0.00%	0
Telugu	0.48%	2
Thai	0.00%	0
Turkish	0.00%	0
Ukrainian	0.00%	0
Urdu	0.00%	0
Uzbek	0.00%	0
Vietnamese	0.00%	0
Wu Chinese	0.00%	0
Yoruba	0.00%	0
TOTAL		415

Q11 Are you familiar with artificial intelligence (AI) in healthcare?

Answered: 415 Skipped: 0

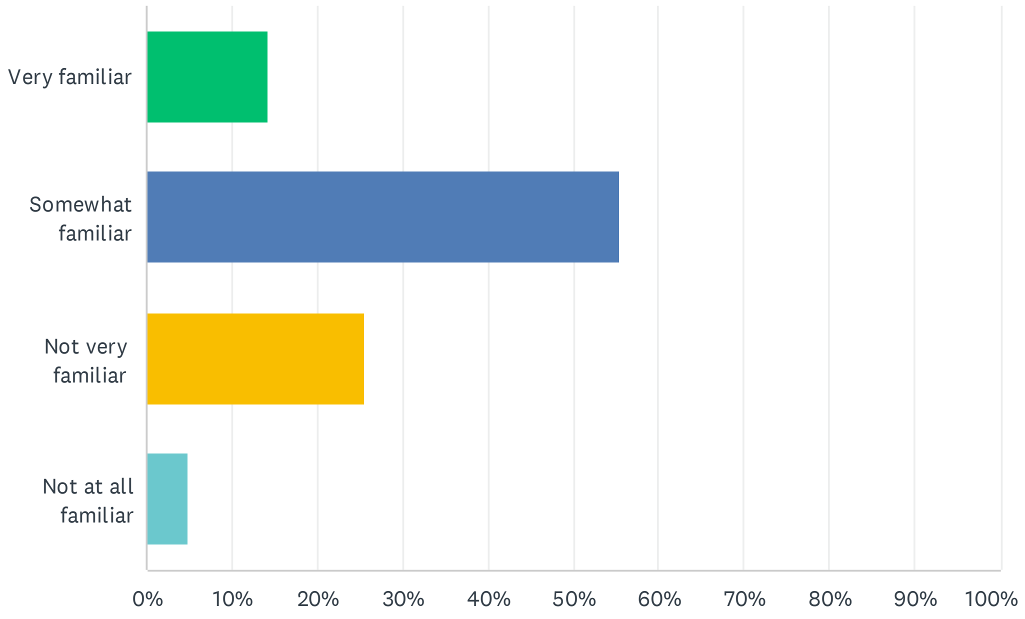

ANSWER CHOICES	RESPONSES	
Very familiar	14.22%	59
Somewhat familiar	55.42%	230
Not very familiar	25.54%	106
Not at all familiar	4.82%	20
TOTAL		415

Q12 Are you familiar with using AI in your medical school experience?

Answered: 415 Skipped: 0

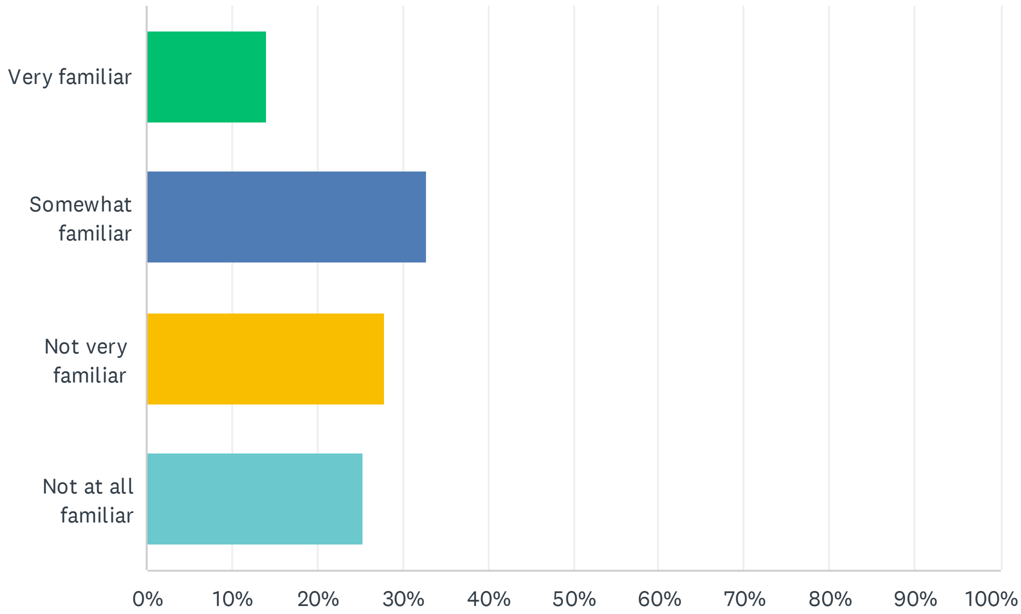

ANSWER CHOICES	RESPONSES	
Very familiar	13.98%	58
Somewhat familiar	32.77%	136
Not very familiar	27.95%	116
Not at all familiar	25.30%	105
TOTAL		415

Q13 Have you heard of ChatGPT (or any other LLMs) before?

Answered: 415 Skipped: 0

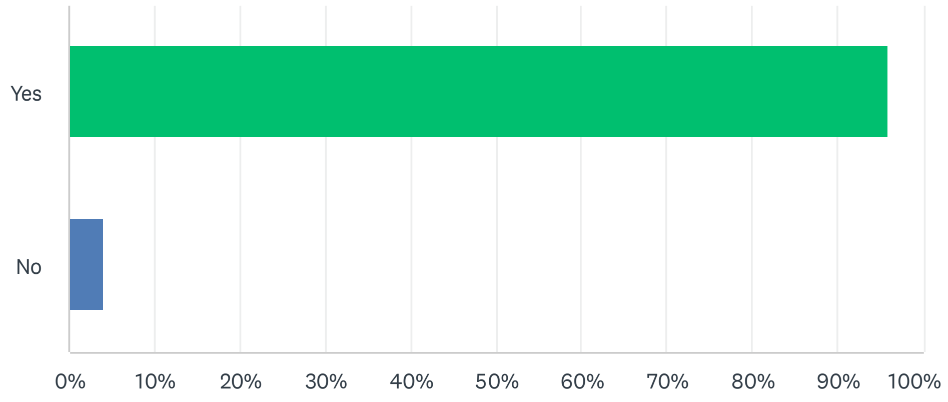

ANSWER CHOICES	RESPONSES	
Yes	95.90%	398
No	4.10%	17
TOTAL		415

Q14 How did you learn about ChatGPT (or any other LLMs)?

Answered: 398 Skipped: 17

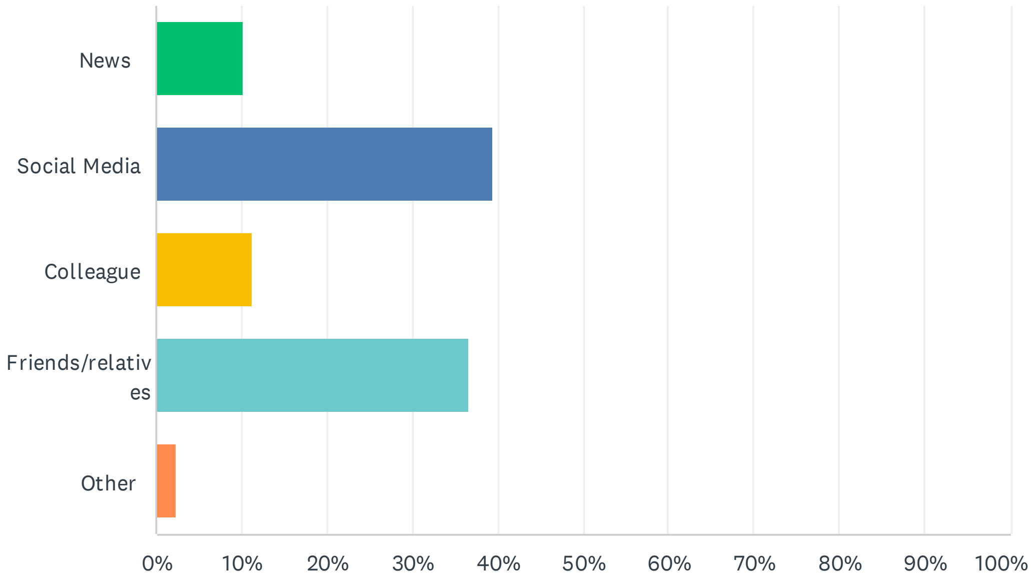

ANSWER CHOICES	RESPONSES	
News	10.30%	41
Social Media	39.45%	157
Colleague	11.31%	45
Friends/relatives	36.68%	146
Other	2.26%	9
TOTAL		398

Q15 Have you ever used ChatGPT (or any other LLMs) related to your medical school experience?

Answered: 398 Skipped: 17

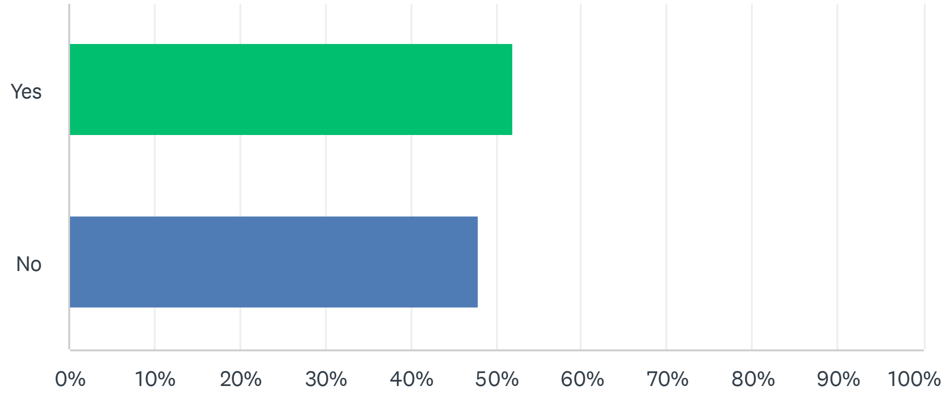

ANSWER CHOICES	RESPONSES	
Yes	52.01%	207
No	47.99%	191
TOTAL		398

Q16 Have you ever used ChatGPT for anything non-medical school related?

Answered: 398 Skipped: 17

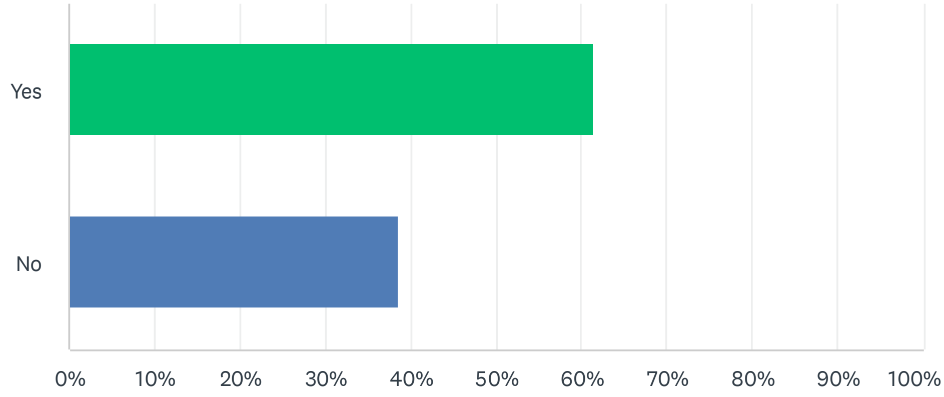

ANSWER CHOICES	RESPONSES	
Yes	61.31%	244
No	38.69%	154
TOTAL		398

Q17 Do you think ChatGPT (or any other LLMs) can have an important role in your medical school experience?

Answered: 398 Skipped: 17

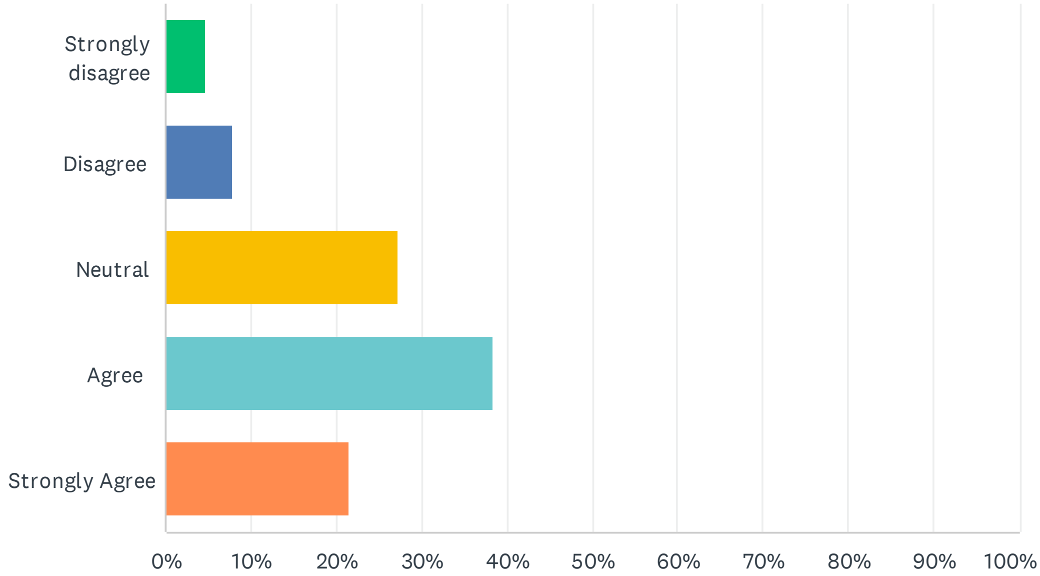

ANSWER CHOICES	RESPONSES	
Strongly disagree	4.77%	19
Disagree	7.79%	31
Neutral	27.39%	109
Agree	38.44%	153
Strongly Agree	21.61%	86
TOTAL		398

Q18 Since when were you first exposed to ChatGPT?

Answered: 398 Skipped: 17

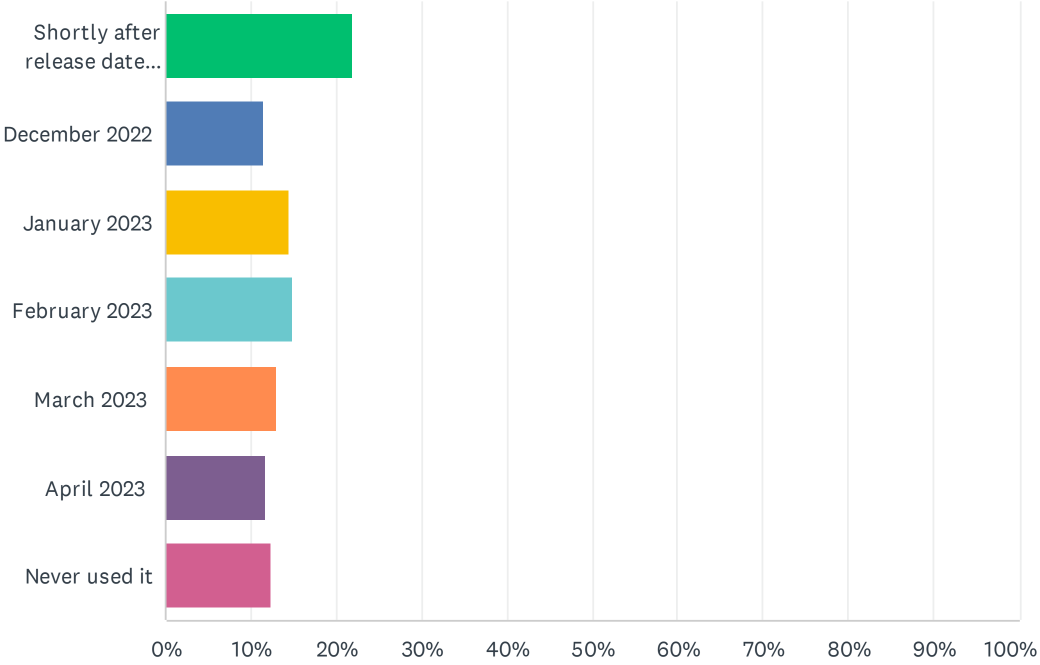

ANSWER CHOICES	RESPONSES	
Shortly after release date (November 2022)	21.86%	87
December 2022	11.56%	46
January 2023	14.57%	58
February 2023	14.82%	59
March 2023	13.07%	52
April 2023	11.81%	47
Never used it	12.31%	49
TOTAL		398

Q19 How do published editorials and studies about ChatGPT (or any other LLMs) shape your view on the capabilities and limitations of ChatGPT (or any other LLMs)?

Answered: 398 Skipped: 17

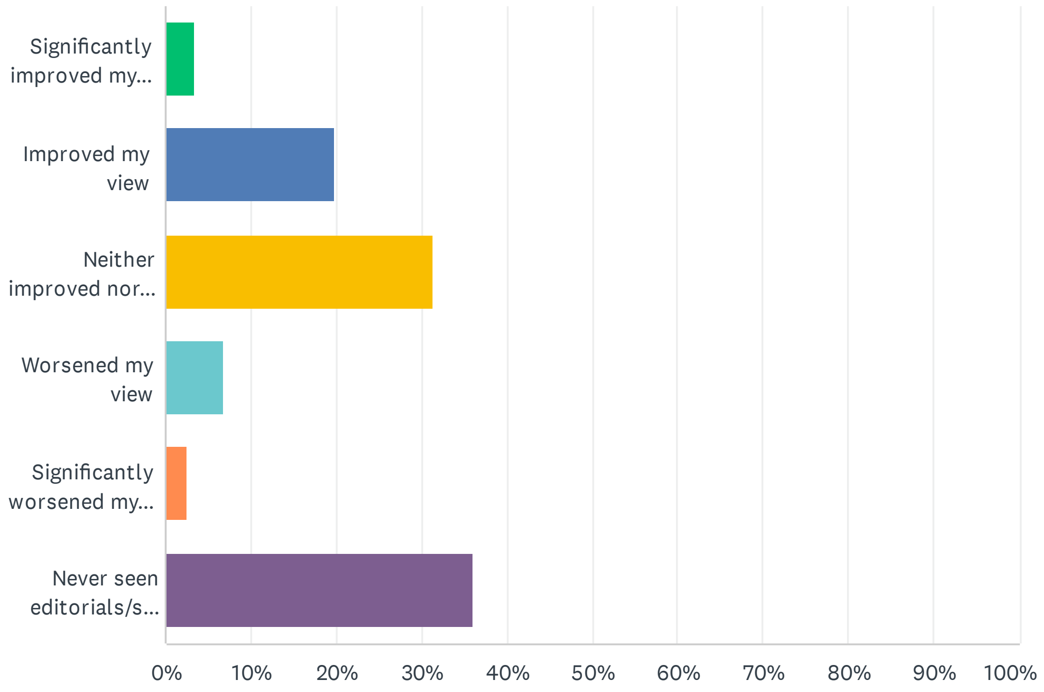

ANSWER CHOICES	RESPONSES	
Significantly improved my view	3.52%	14
Improved my view	19.85%	79
Neither improved nor worsened my view	31.41%	125
Worsened my view	6.78%	27
Significantly worsened my view	2.51%	10
Never seen editorials/studies	35.93%	143
TOTAL		398

Q20 What is your opinion on the usefulness of ChatGPT in your medical school experience?

Answered: 398 Skipped: 17

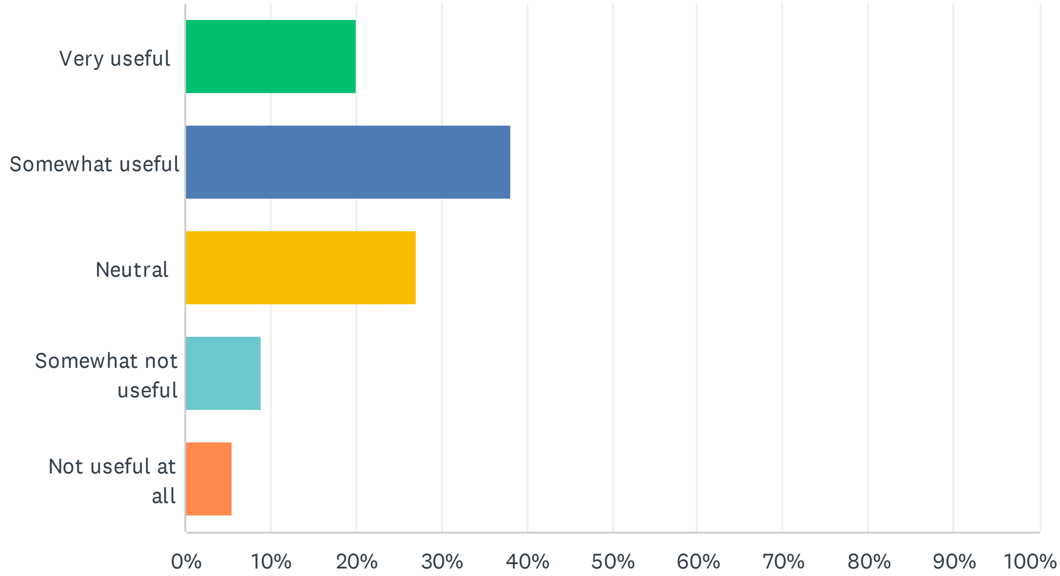

ANSWER CHOICES	RESPONSES	
Very useful	20.10%	80
Somewhat useful	38.19%	152
Neutral	27.14%	108
Somewhat not useful	9.05%	36
Not useful at all	5.53%	22
TOTAL		398

Q21 In what capacity have you used ChatGPT? (Select all that apply)

Answered: 398 Skipped: 17

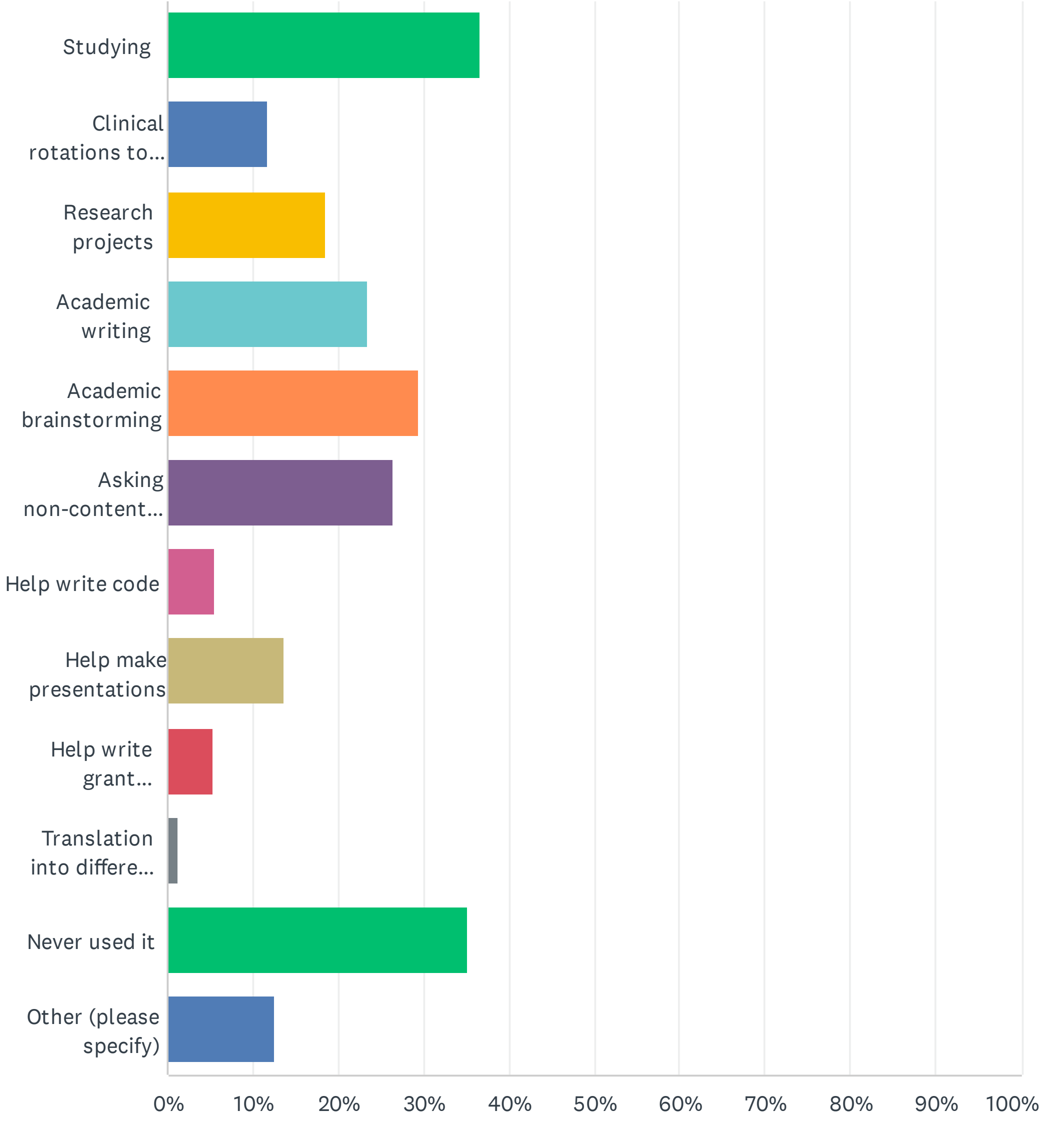

ANSWER CHOICES	RESPONSES	
Studying	36.68%	146
Clinical rotations to look up information	11.81%	47
Research projects	18.59%	74
Academic writing	23.37%	93
Academic brainstorming	29.40%	117
Asking non-content questions related to medical school	26.38%	105
Help write code	5.53%	22
Help make presentations	13.57%	54
Help write grant applications	5.28%	21
Translation into different languages	1.26%	5
Never used it	35.18%	140
Other (please specify)	12.56%	50
Total Respondents: 398		

Q22 Have you experienced any issues or limitations with using ChatGPT (or any other LLMs)?

Answered: 398 Skipped: 17

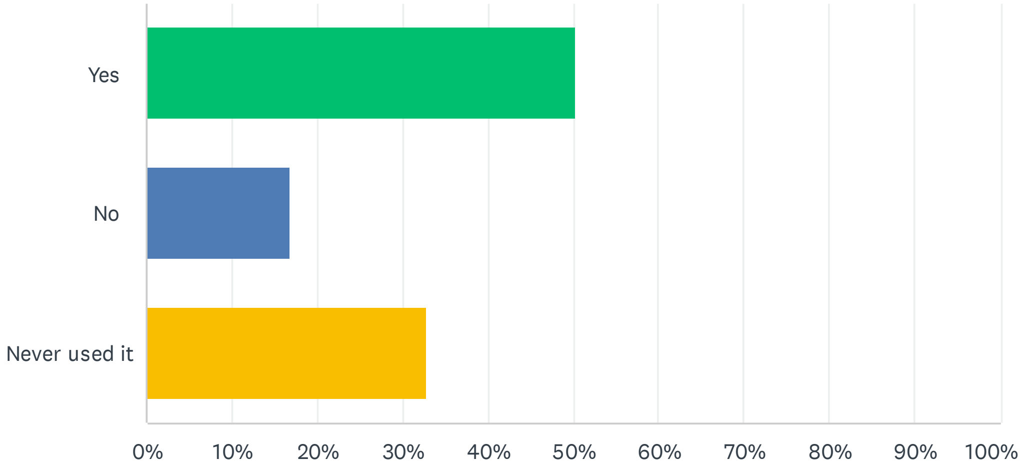

ANSWER CHOICES	RESPONSES	
Yes	50.25%	200
No	16.83%	67
Never used it	32.91%	131
TOTAL		398

Q23 What were the issues or limitations you experienced? (Select all that apply)

Answered: 398 Skipped: 17

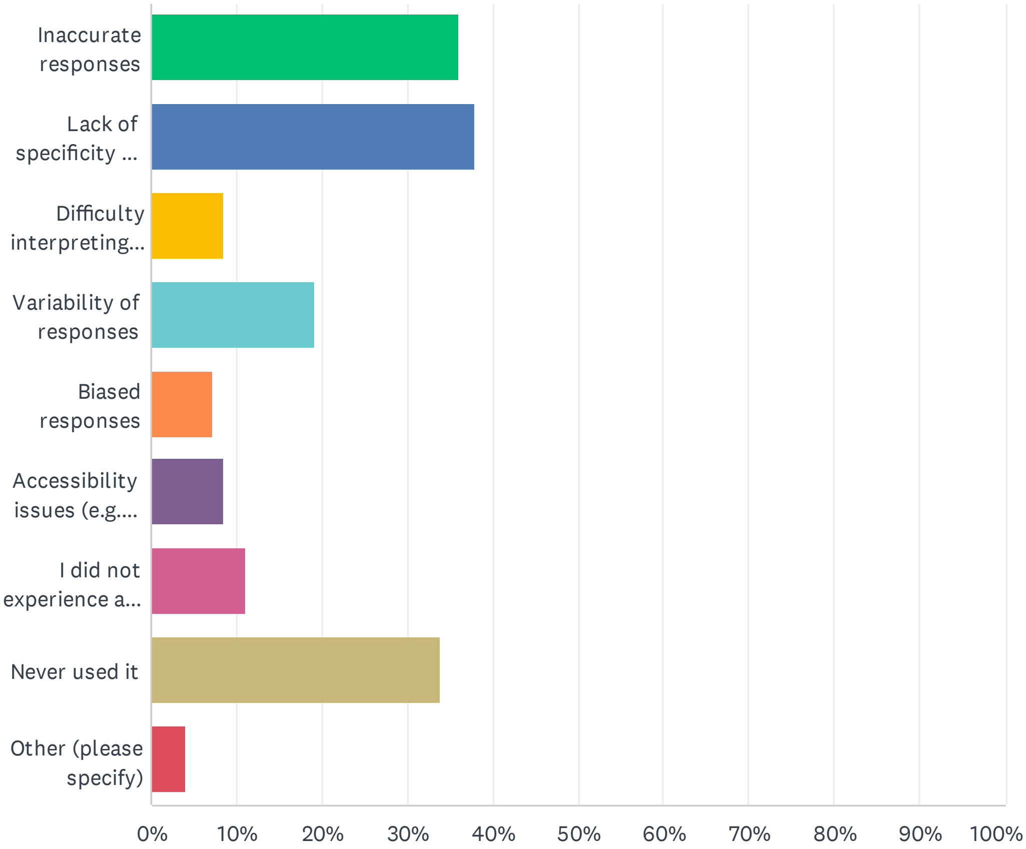

ANSWER CHOICES	RESPONSES	
Inaccurate responses	35.93%	143
Lack of specificity in responses	37.94%	151
Difficulty interpreting responses	8.54%	34
Variability of responses	19.10%	76
Biased responses	7.29%	29
Accessibility issues (e.g. website crashing)	8.54%	34
I did not experience any limitations	11.06%	44
Never used it	33.92%	135
Other (please specify)	4.02%	16
Total Respondents: 398		

Q24 Does ChatGPT (or any other LLMs) help you save time in your task?

Answered: 398 Skipped: 17

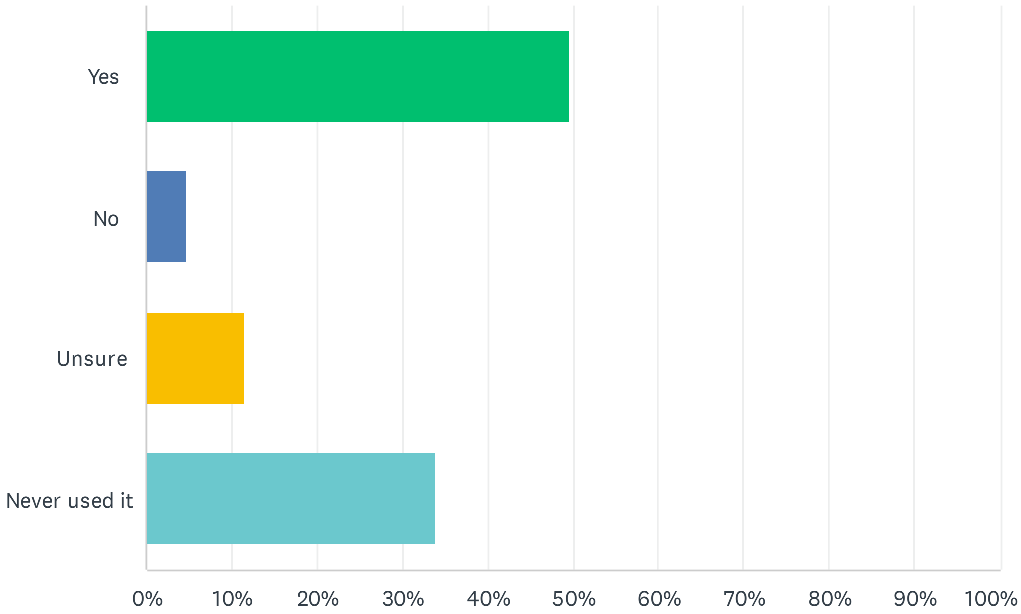

ANSWER CHOICES	RESPONSES	
Yes	49.75%	198
No	4.77%	19
Unsure	11.56%	46
Never used it	33.92%	135
TOTAL		398

Q25 If ChatGPT was no longer free, would you pay to use it?

Answered: 398 Skipped: 17

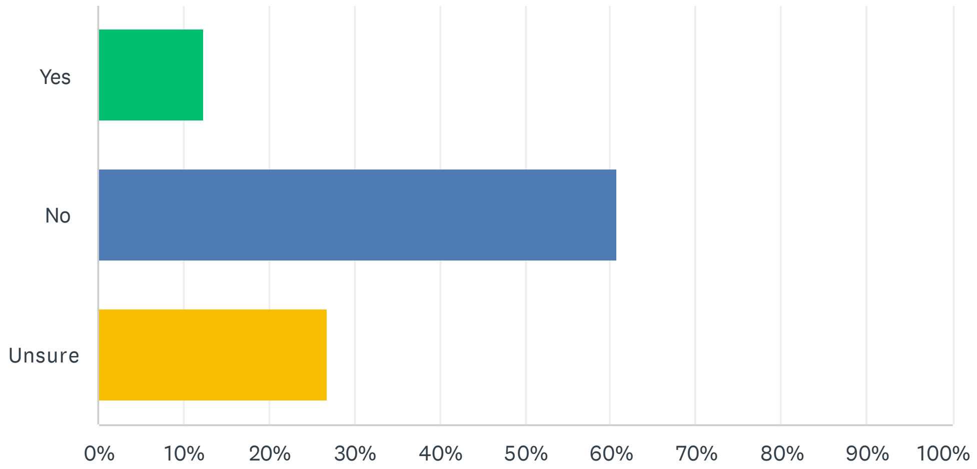

ANSWER CHOICES	RESPONSES	
Yes	12.31%	49
No	60.80%	242
Unsure	26.88%	107
TOTAL		398

Q26 Have you used ChatGPT for studying in medical school?

Answered: 398 Skipped: 17

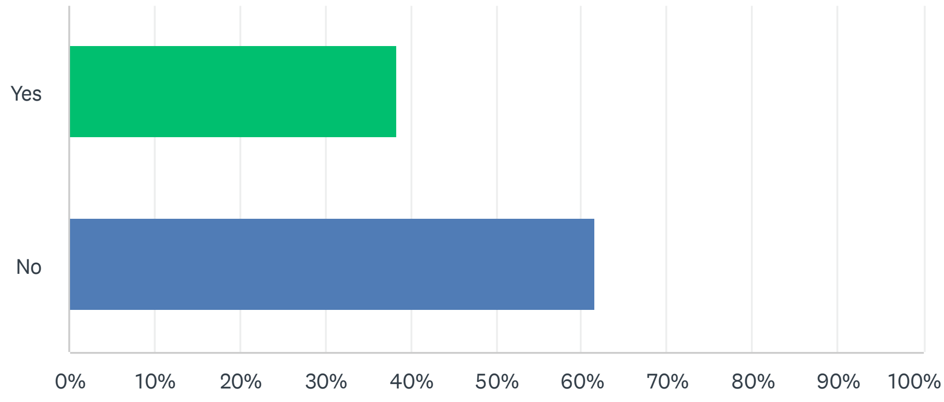

ANSWER CHOICES	RESPONSES	
Yes	38.44%	153
No	61.56%	245
TOTAL		398

Q27 How have you used ChatGPT for studying in medical school?

Answered: 398 Skipped: 17

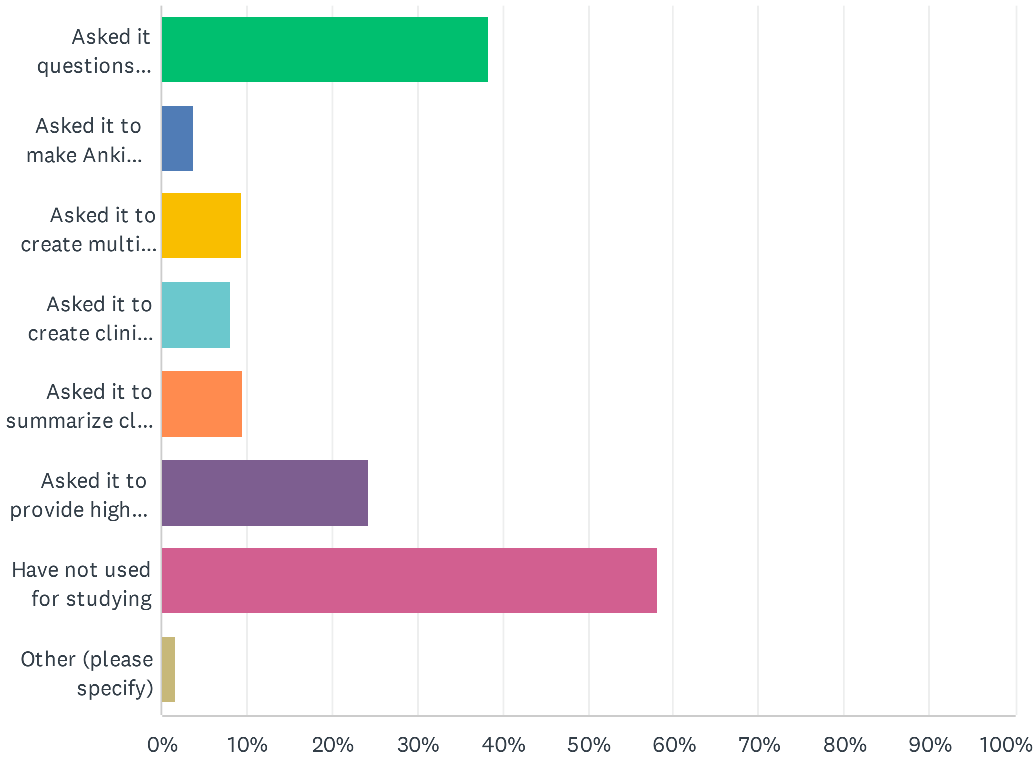

ANSWER CHOICES	RESPONSES	
Asked it questions directly (chatbot function)	38.44%	153
Asked it to make Anki flashcards	3.77%	15
Asked it to create multiple choice questions	9.30%	37
Asked it to create clinical scenarios	8.04%	32
Asked it to summarize class outlines/PowerPoints	9.55%	38
Asked it to provide high yield information on a topic	24.37%	97
Have not used for studying	58.29%	232
Other (please specify)	1.76%	7
Total Respondents: 398		

Q28 How often do you use ChatGPT for studying?

Answered: 398 Skipped: 17

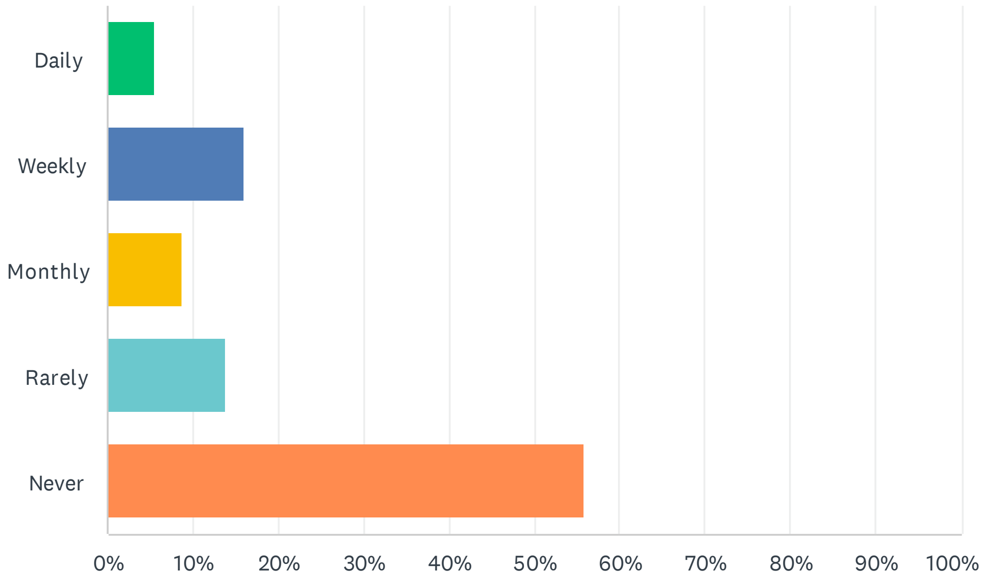

ANSWER CHOICES	RESPONSES	
Daily	5.53%	22
Weekly	16.08%	64
Monthly	8.79%	35
Rarely	13.82%	55
Never	55.78%	222
TOTAL		398

Q29 What is your opinion on the usefulness of ChatGPT for studying?

Answered: 398 Skipped: 17

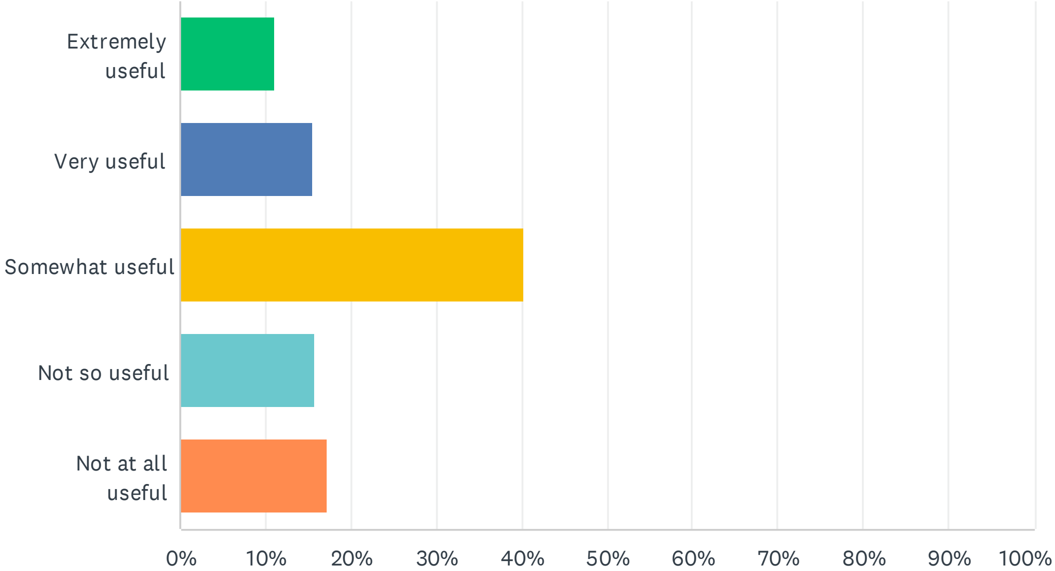

ANSWER CHOICES	RESPONSES	
Extremely useful	11.06%	44
Very useful	15.58%	62
Somewhat useful	40.20%	160
Not so useful	15.83%	63
Not at all useful	17.34%	69
TOTAL		398

Q30 Have you experienced any issues or limitations with using ChatGPT for studying?

Answered: 398 Skipped: 17

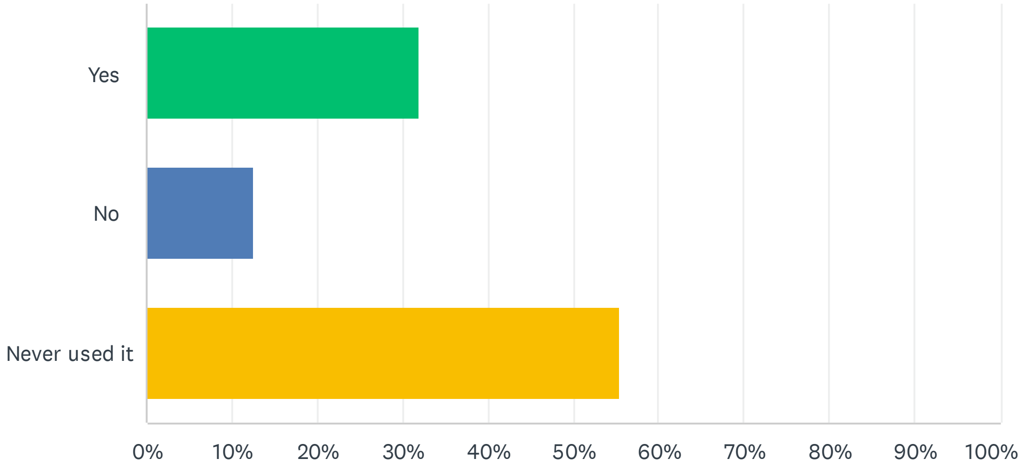

ANSWER CHOICES	RESPONSES	
Yes	31.91%	127
No	12.56%	50
Never used it	55.53%	221
TOTAL		398

Q31 If yes, what were the issues or limitations you experienced? (Select all that apply)

Answered: 398 Skipped: 17

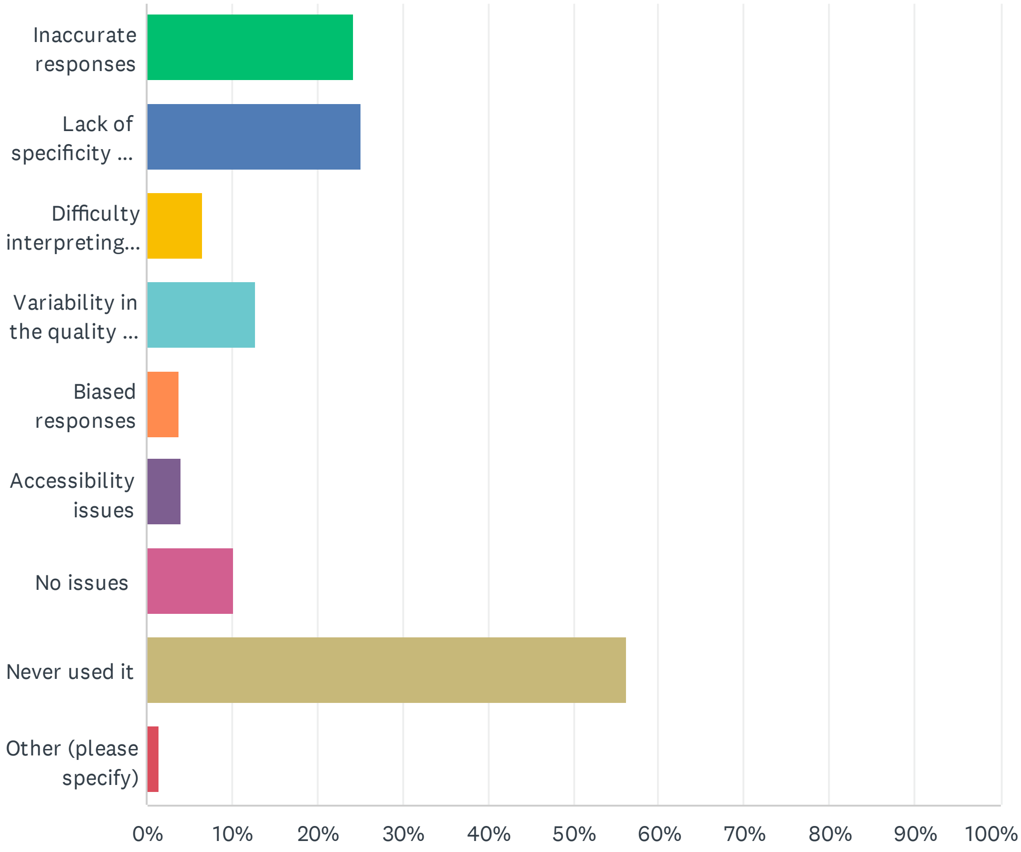

ANSWER CHOICES	RESPONSES	
Inaccurate responses	24.37%	97
Lack of specificity in responses	25.13%	100
Difficulty interpreting responses	6.53%	26
Variability in the quality of responses	12.81%	51
Biased responses	3.77%	15
Accessibility issues	4.02%	16
No issues	10.30%	41
Never used it	56.28%	224
Other (please specify)	1.51%	6
Total Respondents: 398		

Q32 Do you use Anki to study in medical school?

Answered: 398 Skipped: 17

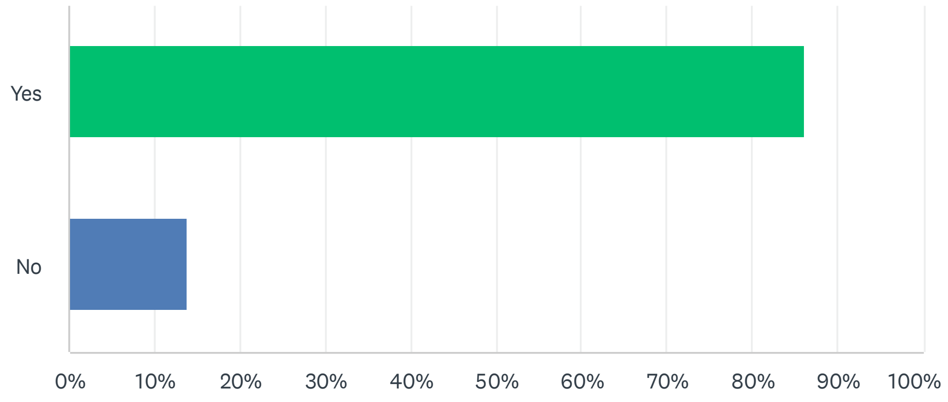

ANSWER CHOICES	RESPONSES	
Yes	86.18%	343
No	13.82%	55
TOTAL		398

Q33 How frequently do you use Anki

Answered: 343 Skipped: 72

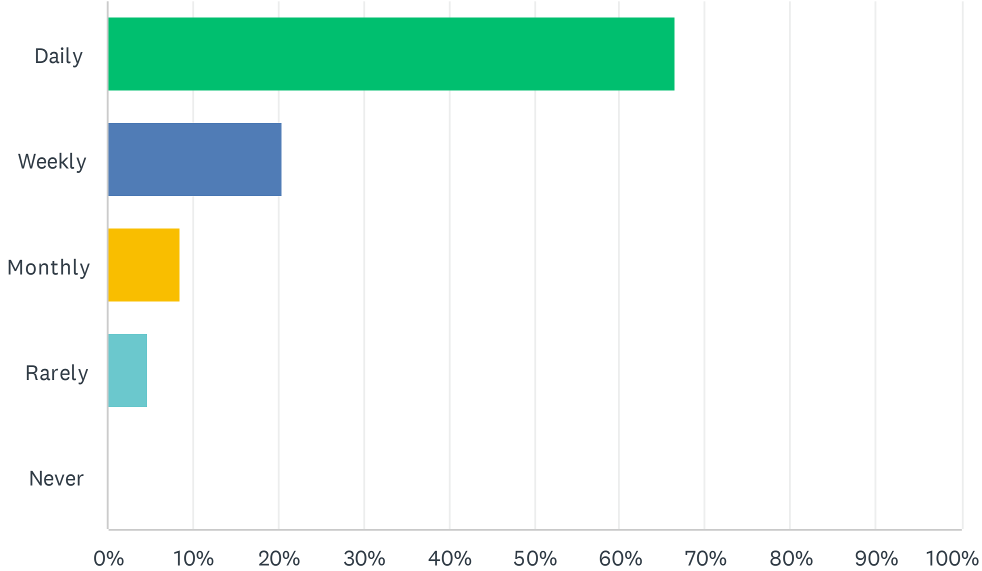

ANSWER CHOICES	RESPONSES	
Daily	66.47%	228
Weekly	20.41%	70
Monthly	8.45%	29
Rarely	4.66%	16
Never	0.00%	0
TOTAL		343

Q34 Have you used ChatGPT to make Anki flashcards?

Answered: 343 Skipped: 72

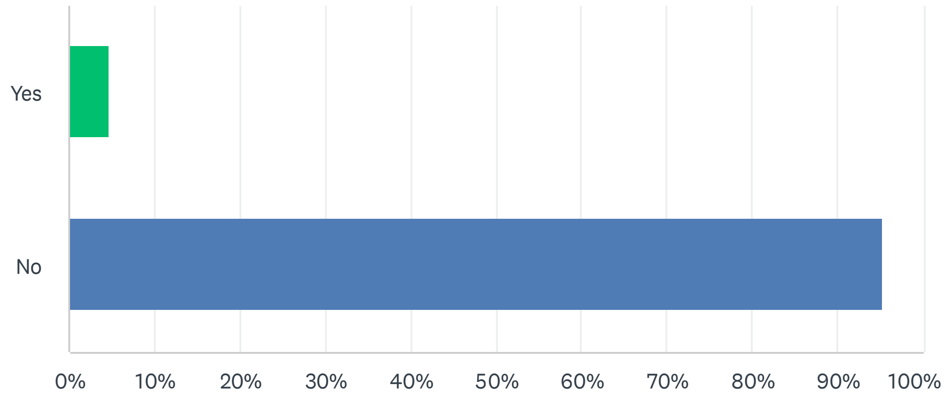

ANSWER CHOICES	RESPONSES	
Yes	4.66%	16
No	95.34%	327
TOTAL		343

Q35 Have you used ChatGPT while using Anki to ask follow-up questions or clarify information on Anki cards?

Answered: 343 Skipped: 72

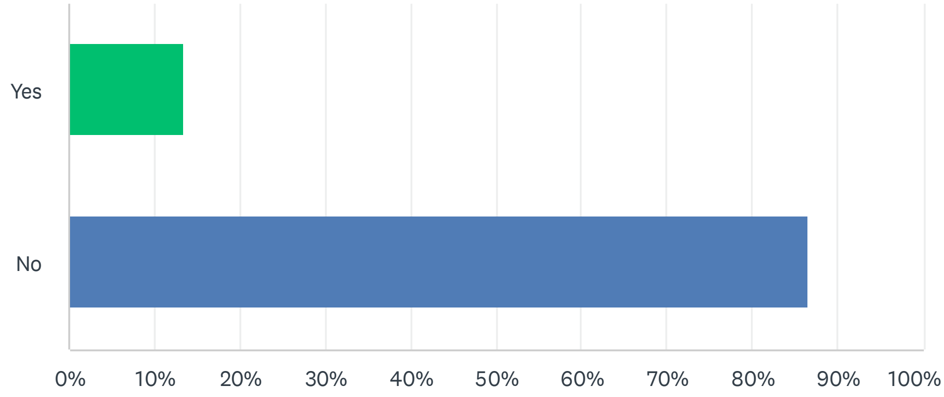

ANSWER CHOICES	RESPONSES	
Yes	13.41%	46
No	86.59%	297
TOTAL		343

Q36 If there were resources available demonstrating how to use ChatGPT to make Anki cards, would you use it?

Answered: 398 Skipped: 17

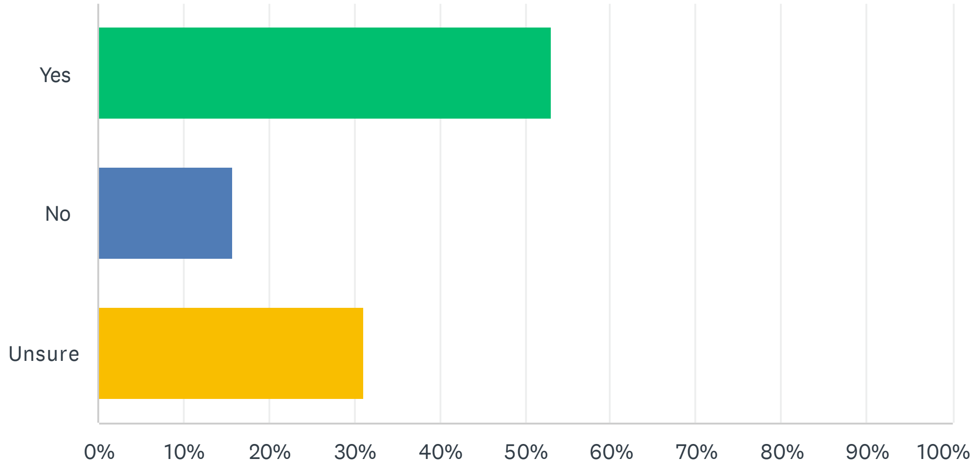

ANSWER CHOICES	RESPONSES	
Yes	53.02%	211
No	15.83%	63
Unsure	31.16%	124
TOTAL		398

Q37 Do you think ChatGPT could ever replace Anki for studying

Answered: 398 Skipped: 17

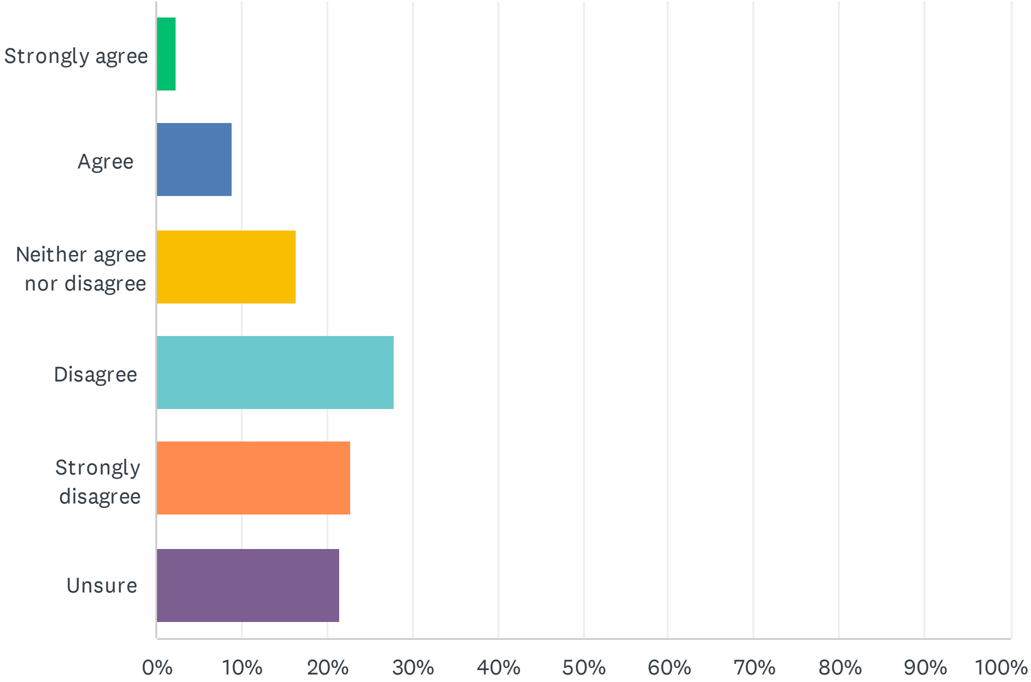

ANSWER CHOICES	RESPONSES	
Strongly agree	2.26%	9
Agree	9.05%	36
Neither agree nor disagree	16.33%	65
Disagree	27.89%	111
Strongly disagree	22.86%	91
Unsure	21.61%	86
TOTAL		398

Q38 Have you used ChatGPT to make practice multiple choice questions?

Answered: 398 Skipped: 17

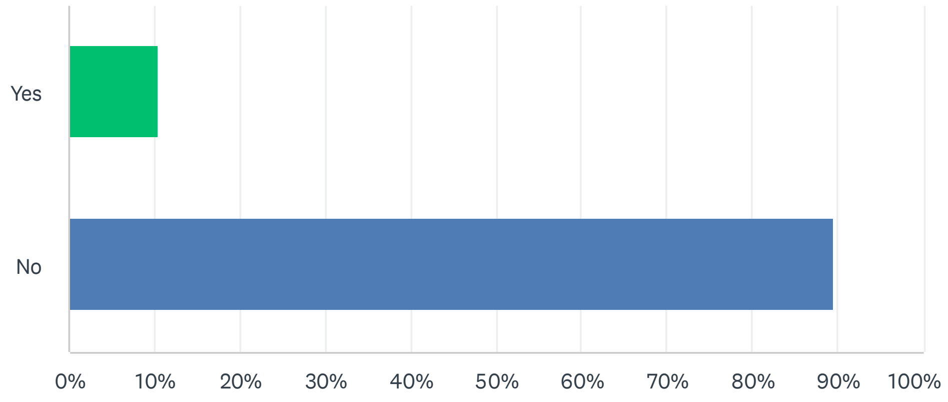

ANSWER CHOICES	RESPONSES	
Yes	10.55%	42
No	89.45%	356
TOTAL		398

Q39 If yes, how useful were these questions?

Answered: 42 Skipped: 373

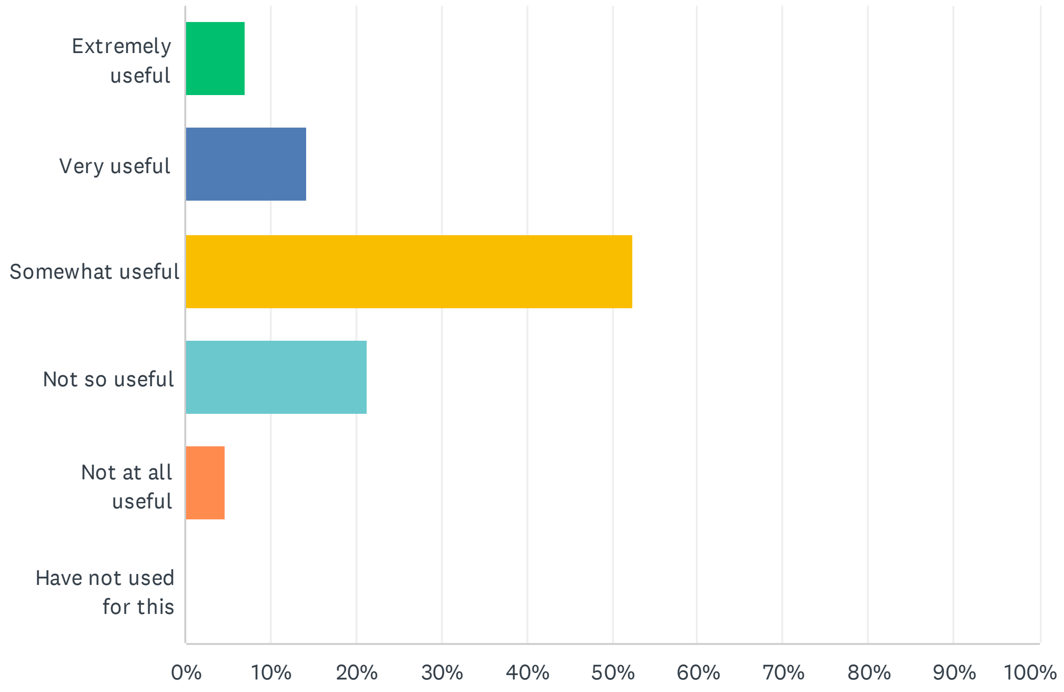

ANSWER CHOICES	RESPONSES	
Extremely useful	7.14%	3
Very useful	14.29%	6
Somewhat useful	52.38%	22
Not so useful	21.43%	9
Not at all useful	4.76%	2
Have not used for this	0.00%	0
TOTAL		42

Q40 If there were resources available demonstrating how to use ChatGPT to make multiple choice questions, would you use it?

Answered: 398 Skipped: 17

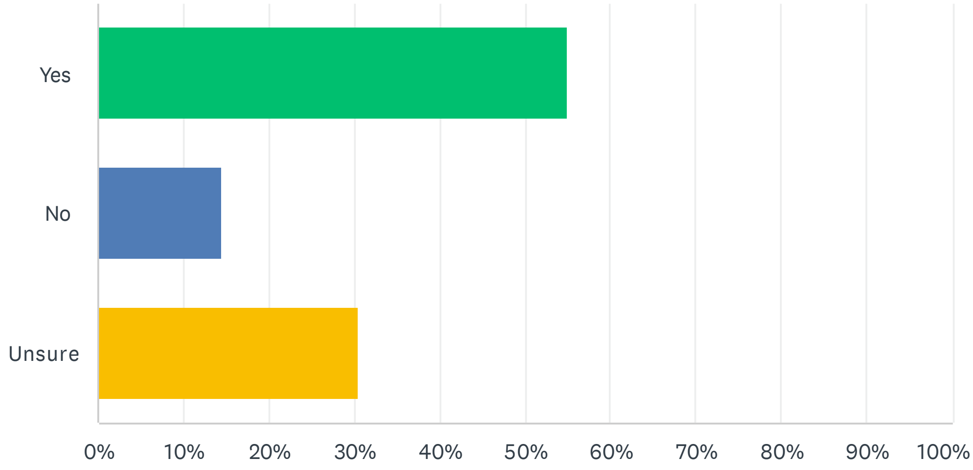

ANSWER CHOICES	RESPONSES	
Yes	55.03%	219
No	14.57%	58
Unsure	30.40%	121
TOTAL		398

Q41 Have you used ChatGPT to make summarize class outlines/PowerPoints?

Answered: 398 Skipped: 17

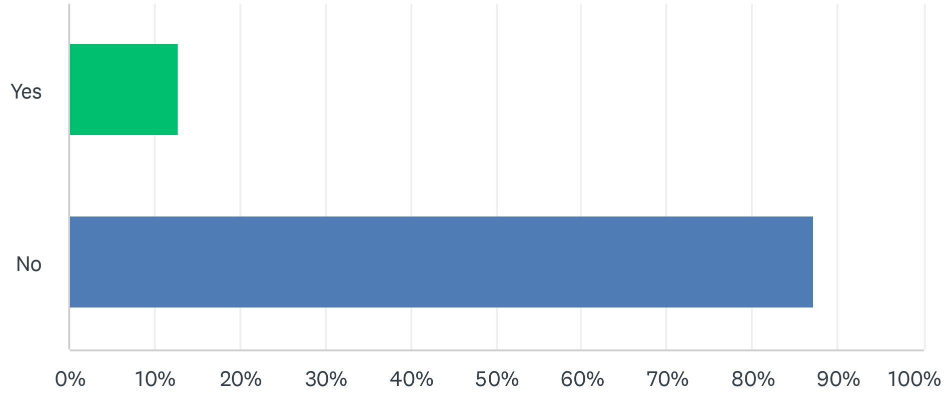

ANSWER CHOICES	RESPONSES	
Yes	12.81%	51
No	87.19%	347
TOTAL		398

Q42 If yes, how useful was ChatGPT in summarizing class outlines/PowerPoints?

Answered: 51 Skipped: 364

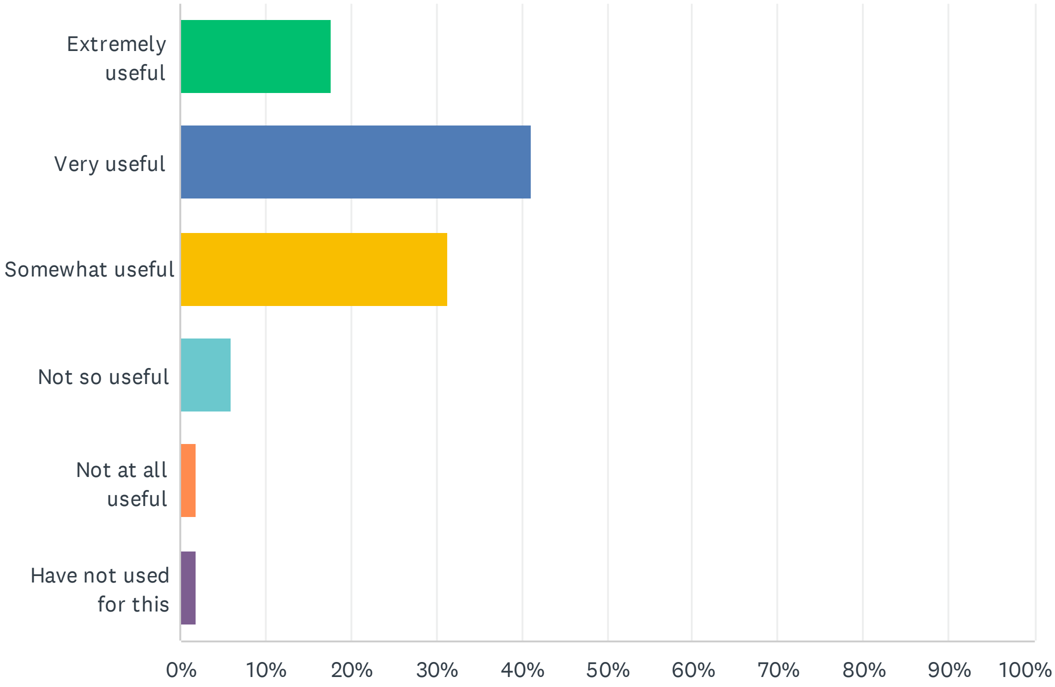

ANSWER CHOICES	RESPONSES	
Extremely useful	17.65%	9
Very useful	41.18%	21
Somewhat useful	31.37%	16
Not so useful	5.88%	3
Not at all useful	1.96%	1
Have not used for this	1.96%	1
TOTAL		51

Q43 Have you been in a lecture (in-person or virtual) at some-point over the prior 6 months?

Answered: 398 Skipped: 17

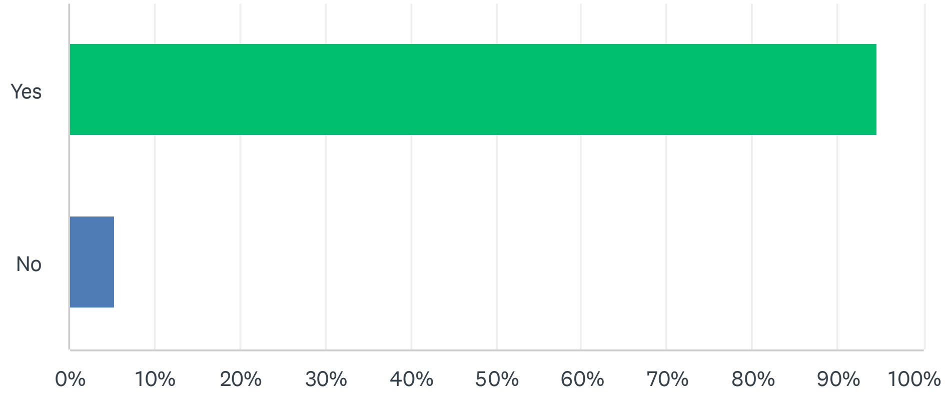

ANSWER CHOICES	RESPONSES	
Yes	94.72%	377
No	5.28%	21
TOTAL		398

Q44 Have you used ChatGPT to look up information during lecture?

Answered: 377 Skipped: 38

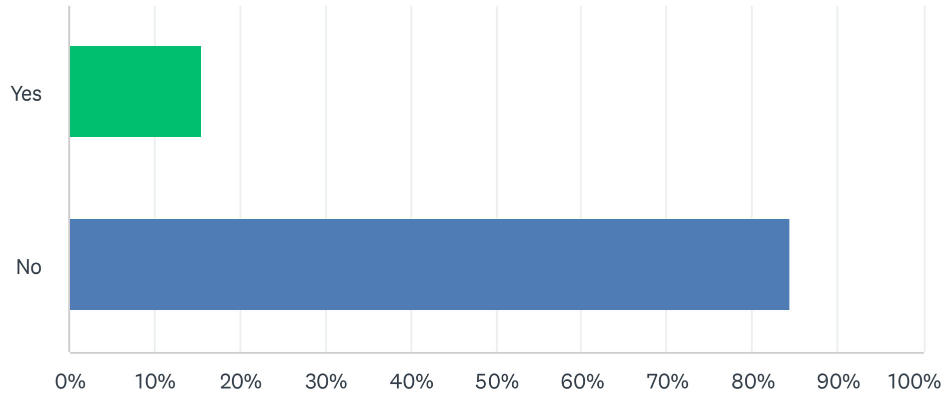

ANSWER CHOICES	RESPONSES	
Yes	15.65%	59
No	84.35%	318
TOTAL		377

Q45 To your knowledge, has any professor/instructor used ChatGPT to support their teaching?

Answered: 377 Skipped: 38

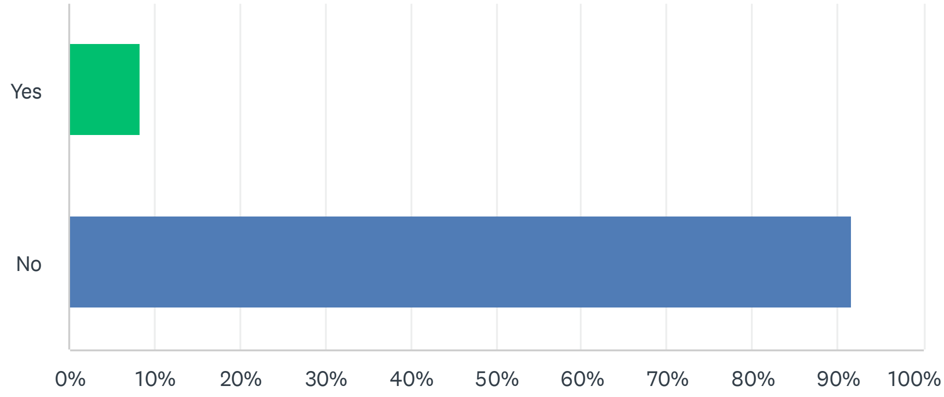

ANSWER CHOICES	RESPONSES	
Yes	8.22%	31
No	91.78%	346
TOTAL		377

Q46 If yes, how has a professor/instructor used ChatGPT to support teaching?

Answered: 32 Skipped: 383

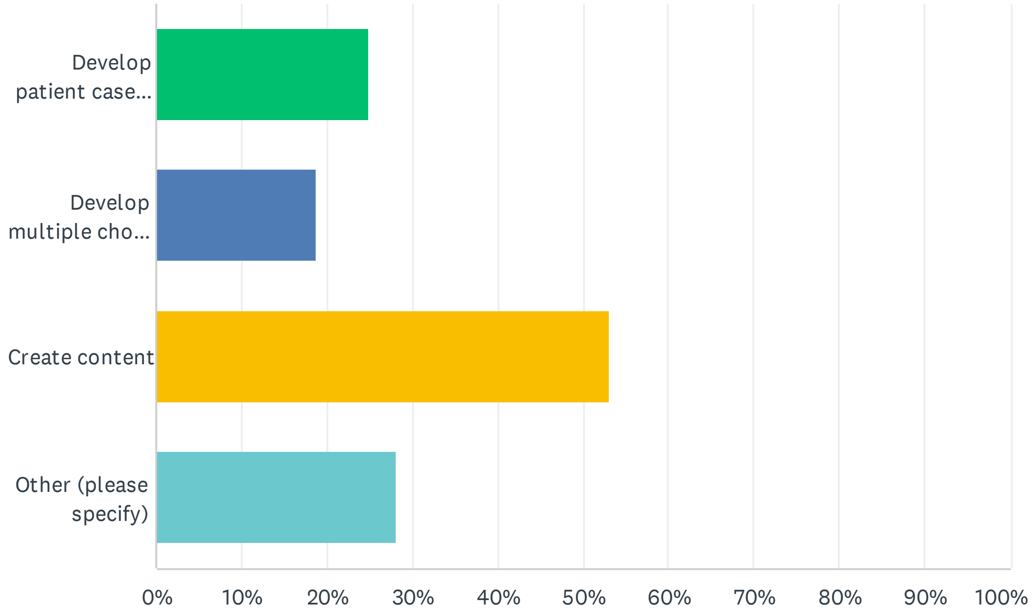

ANSWER CHOICES	RESPONSES	
Develop patient case scenarios	25.00%	8
Develop multiple choice questions	18.75%	6
Create content	53.13%	17
Other (please specify)	28.13%	9
Total Respondents: 32		

Q47 Have you been on clinical rotations at some point in the last 6 months (core clerkships, sub-i, away rotations, electives, ect.)

Answered: 398 Skipped: 17

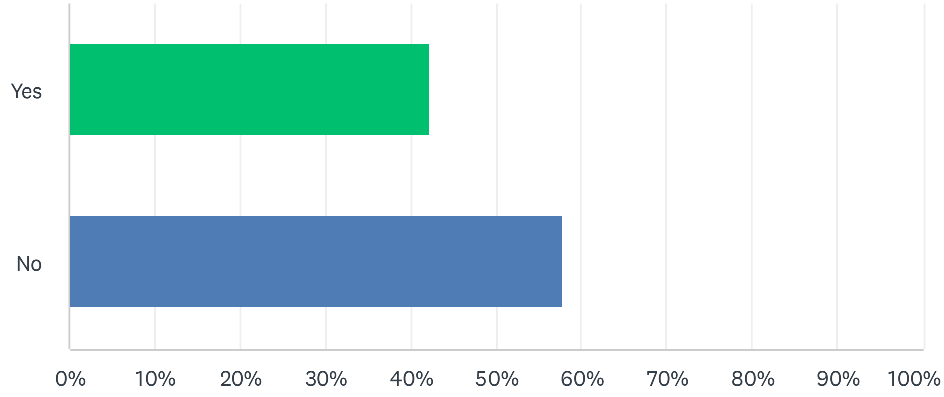

ANSWER CHOICES	RESPONSES	
Yes	42.21%	168
No	57.79%	230
TOTAL		398

Q48 Have you used ChatGPT to look up information while on a clinical rotation?

Answered: 169 Skipped: 246

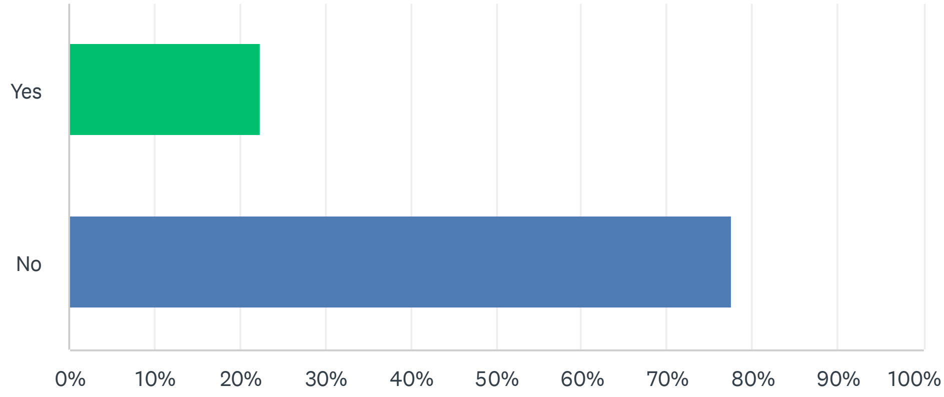

ANSWER CHOICES	RESPONSES	
Yes	22.49%	38
No	77.51%	131
TOTAL		169

Q49 For clinical rotations: what do you use ChatGPT to help look up information for? (Select all that apply)

Answered: 38 Skipped: 377

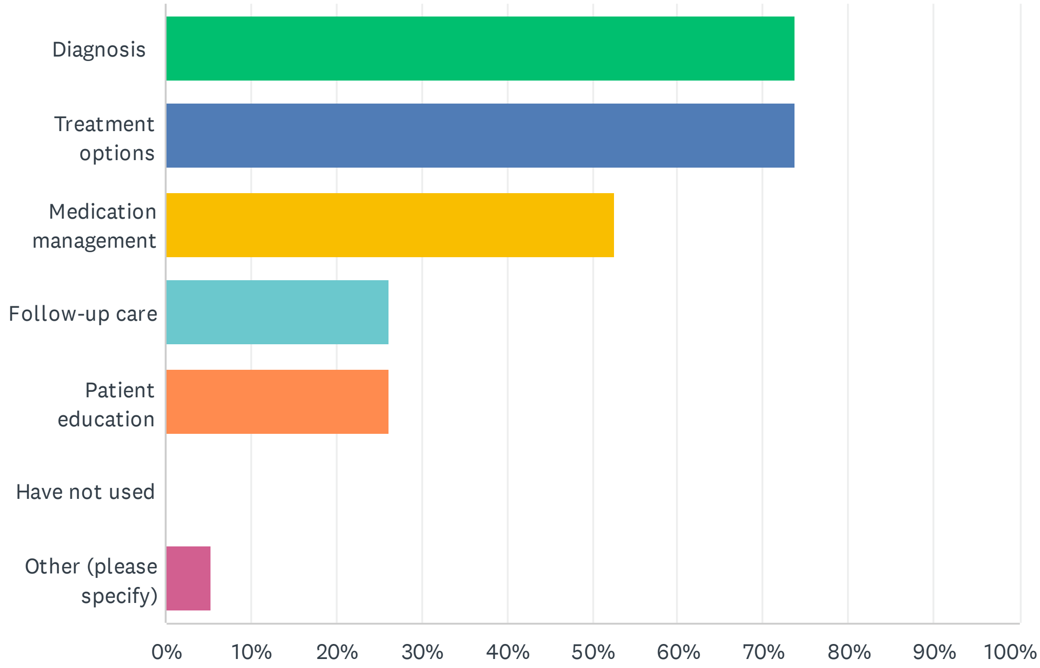

ANSWER CHOICES	RESPONSES	
Diagnosis	73.68%	28
Treatment options	73.68%	28
Medication management	52.63%	20
Follow-up care	26.32%	10
Patient education	26.32%	10
Have not used	0.00%	0
Other (please specify)	5.26%	2
Total Respondents: 38		

Q50 How often do you use ChatGPT in your clinical rotations?

Answered: 168 Skipped: 247

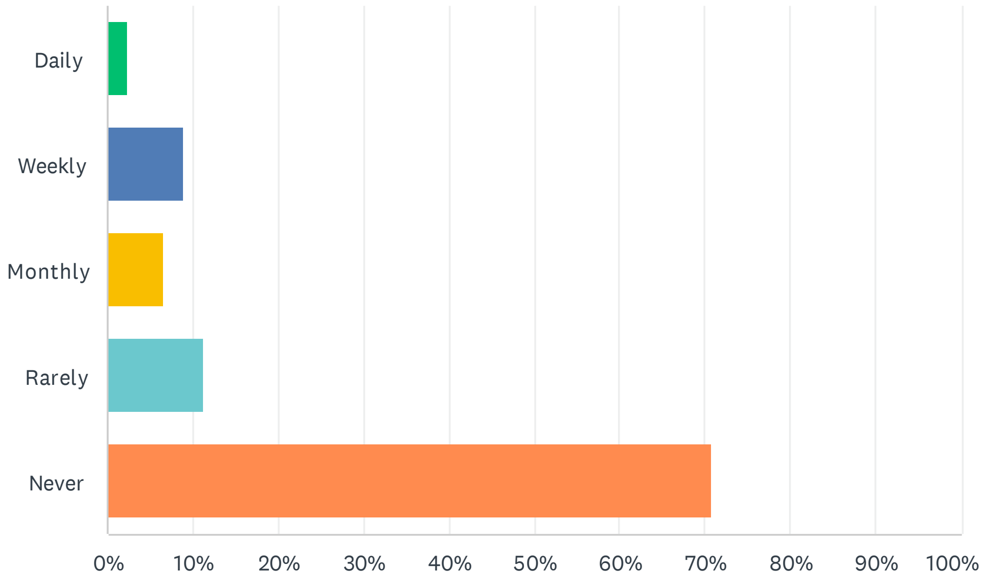

ANSWER CHOICES	RESPONSES	
Daily	2.38%	4
Weekly	8.93%	15
Monthly	6.55%	11
Rarely	11.31%	19
Never	70.83%	119
TOTAL		168

Q51 What is your opinion on the usefulness of ChatGPT in your clinical rotations?

Answered: 168 Skipped: 247

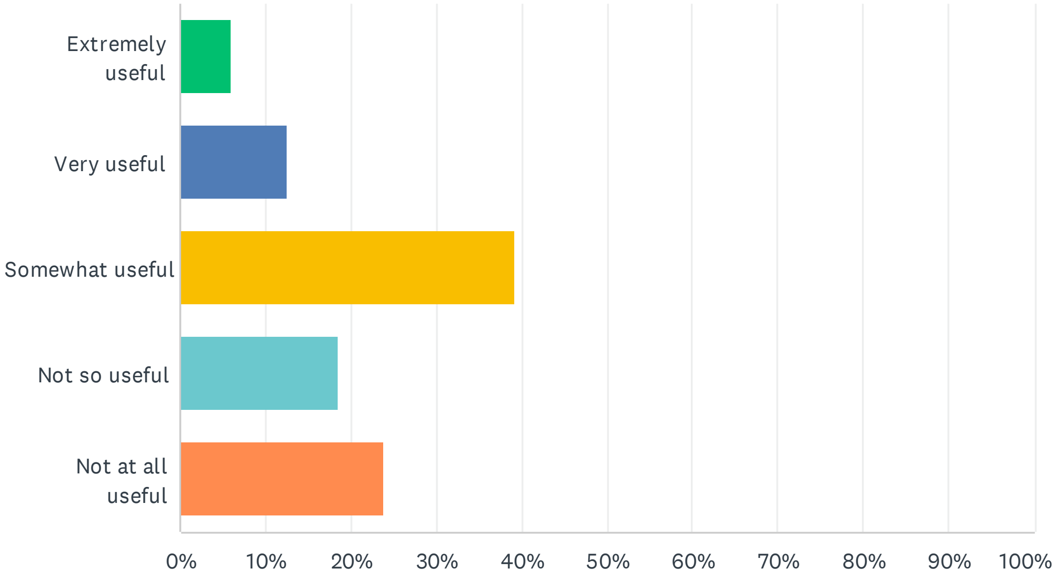

ANSWER CHOICES	RESPONSES	
Extremely useful	5.95%	10
Very useful	12.50%	21
Somewhat useful	39.29%	66
Not so useful	18.45%	31
Not at all useful	23.81%	40
TOTAL		168

Q52 Have you experienced any issues or limitations with using ChatGPT in your clinical rotations?

Answered: 168 Skipped: 247

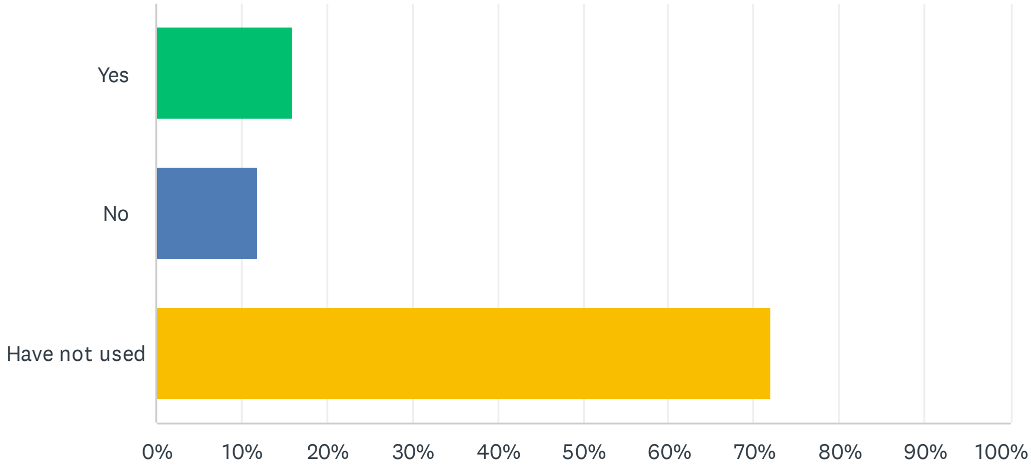

ANSWER CHOICES	RESPONSES	
Yes	16.07%	27
No	11.90%	20
Have not used	72.02%	121
TOTAL		168

Q53 If yes, what were the issues or limitations you experienced? (Select all that apply)

Answered: 168 Skipped: 247

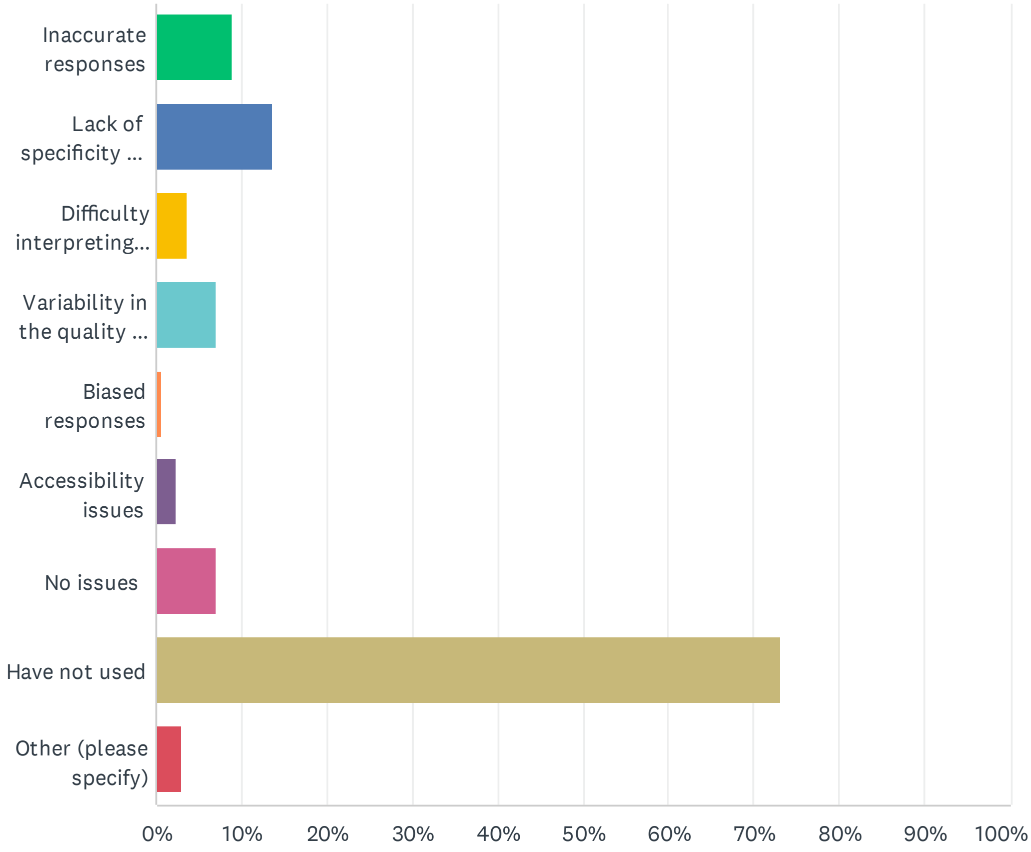

ANSWER CHOICES	RESPONSES	
Inaccurate responses	8.93%	15
Lack of specificity in responses	13.69%	23
Difficulty interpreting responses	3.57%	6
Variability in the quality of responses	7.14%	12
Biased responses	0.60%	1
Accessibility issues	2.38%	4
No issues	7.14%	12
Have not used	73.21%	123
Other (please specify)	2.98%	5
Total Respondents: 168		

Q54 What aspects of clinical practice do you think could benefit from the use of ChatGPT?

Answered: 168 Skipped: 247

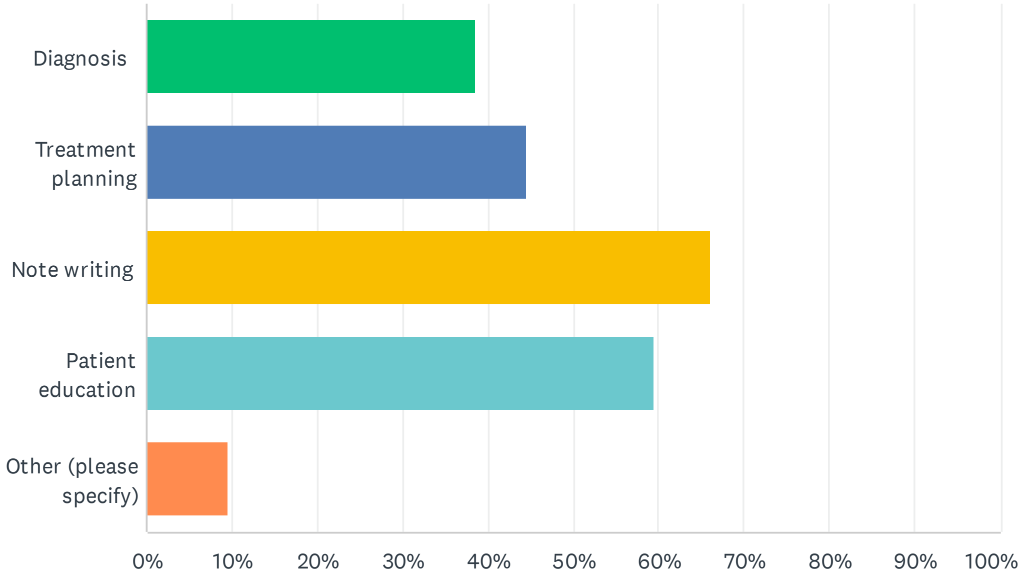

ANSWER CHOICES	RESPONSES	
Diagnosis	38.69%	65
Treatment planning	44.64%	75
Note writing	66.07%	111
Patient education	59.52%	100
Other (please specify)	9.52%	16
Total Respondents: 168		

Q55 Do you pay to use resources to rapidly look up clinical information (ie. AMBOSS, ect.)?

Answered: 168 Skipped: 247

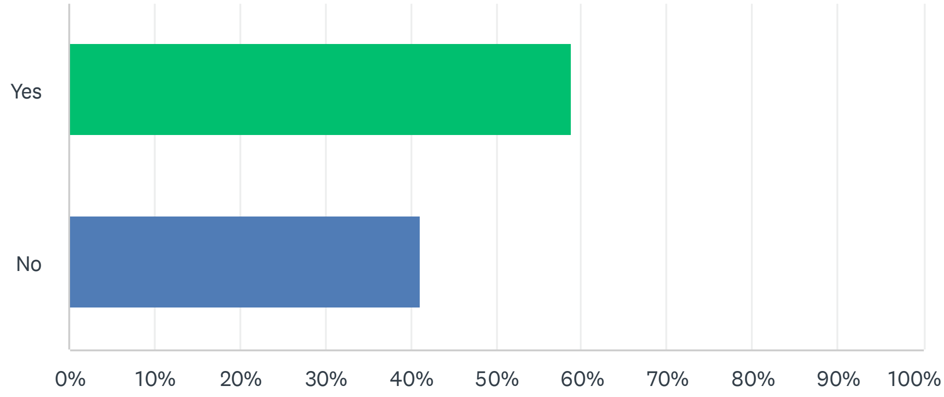

ANSWER CHOICES	RESPONSES	
Yes	58.93%	99
No	41.07%	69
TOTAL		168

Q56 What resources do you use to quickly look up clinical information while on clinical rotations? (select all that apply)

Answered: 168 Skipped: 247

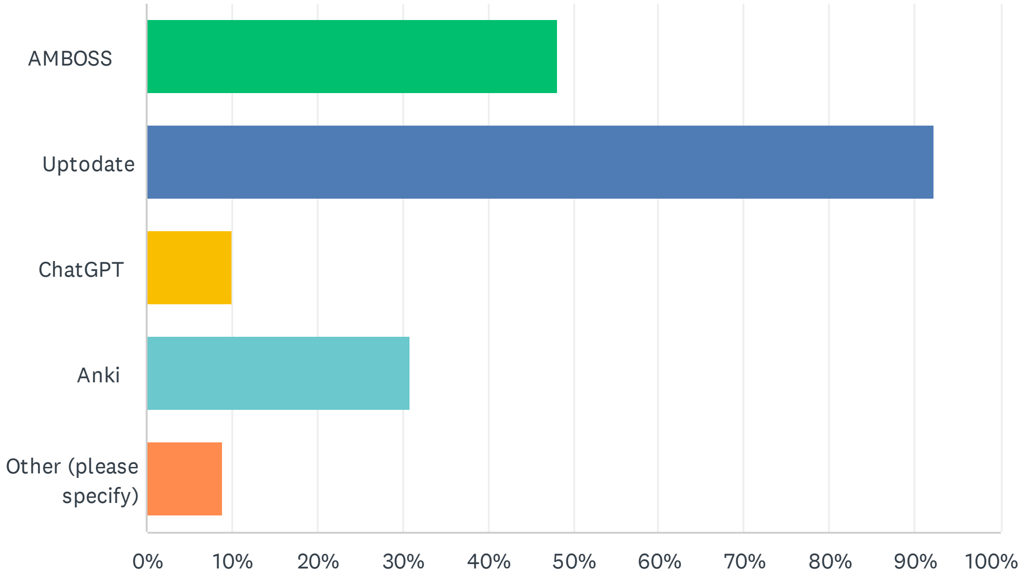

ANSWER CHOICES	RESPONSES	
AMBOSS	48.21%	81
Uptodate	92.26%	155
ChatGPT	10.12%	17
Anki	30.95%	52
Other (please specify)	8.93%	15
Total Respondents: 168		

Q57 If ChatGPT was shown to be reliable and accurate, would you stop paying for paid resources like AMBOSS to have access to clinical information?

Answered: 168 Skipped: 247

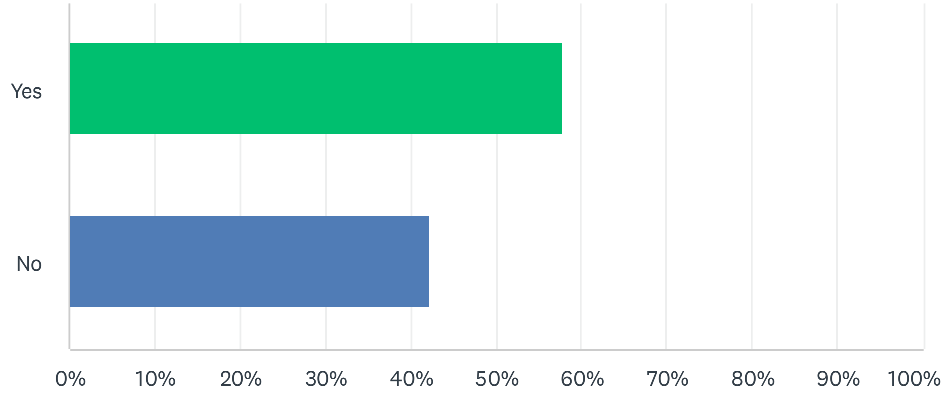

ANSWER CHOICES	RESPONSES	
Yes	57.74%	97
No	42.26%	71
TOTAL		168

Q58 Have you used ChatGPT to help write or edit patient clinical notes?

Answered: 168 Skipped: 247

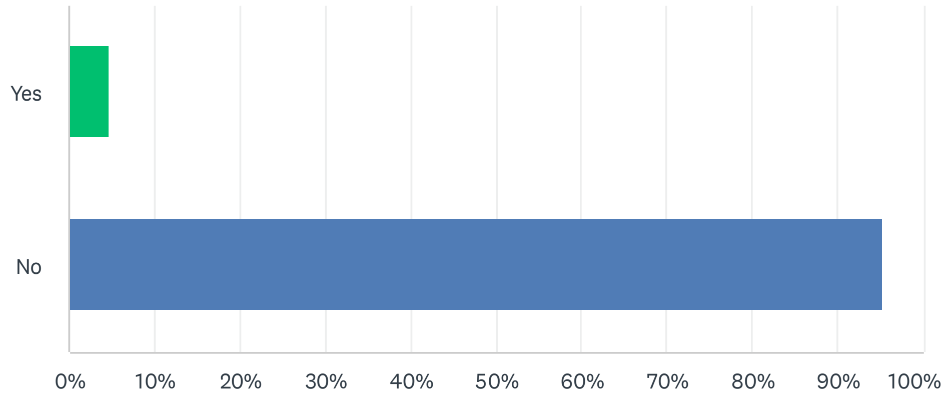

ANSWER CHOICES	RESPONSES	
Yes	4.76%	8
No	95.24%	160
TOTAL		168

Q59 How have you used ChatGPT to help write or edit patient clinical notes?

Answered: 8 Skipped: 407

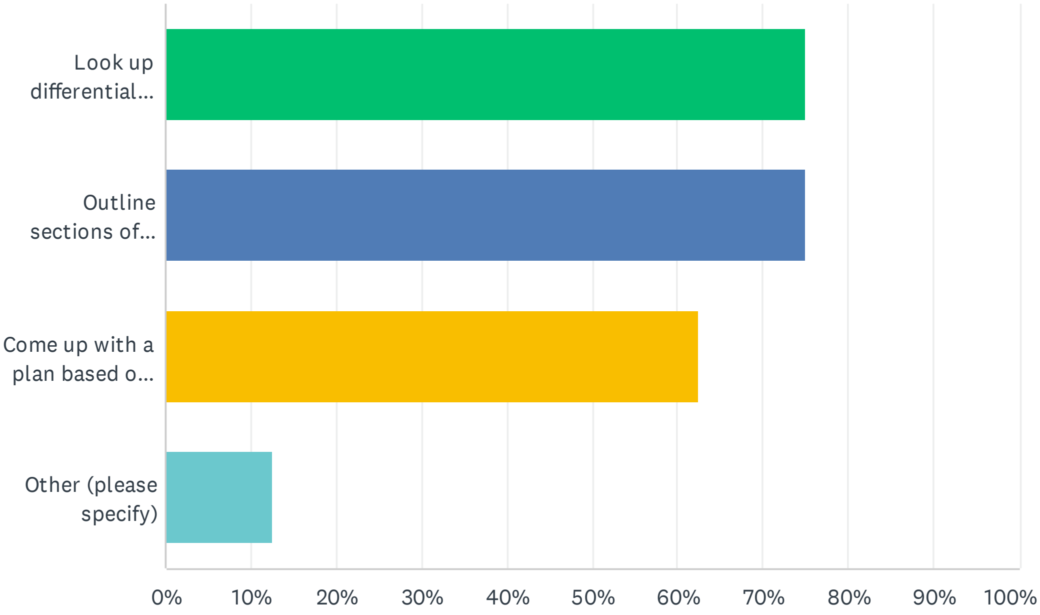

ANSWER CHOICES	RESPONSES	
Look up differential diagnosis	75.00%	6
Outline sections of note (ie. template for physical exam)	75.00%	6
Come up with a plan based on a patient signs/symptoms	62.50%	5
Other (please specify)	12.50%	1
Total Respondents: 8		

Q60 What is your view on patient privacy/security related to ChatGPT?

Answered: 398 Skipped: 17

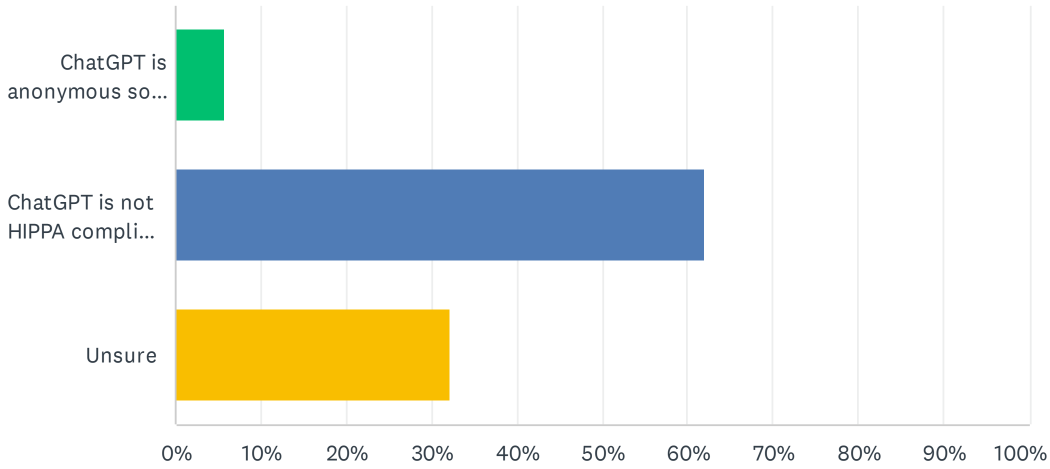

ANSWER CHOICES	RESPONSES	
ChatGPT is anonymous so I can input patient information without violating HIPPA	5.78%	23
ChatGPT is not HIPPA compliant so any use of patient information is a HIPPA violation	62.06%	247
Unsure	32.16%	128
TOTAL		398

Q61 Are you involved in research (i.e., academic publishing, wet/dry lab, clinical trial involvement)?

Answered: 398 Skipped: 17

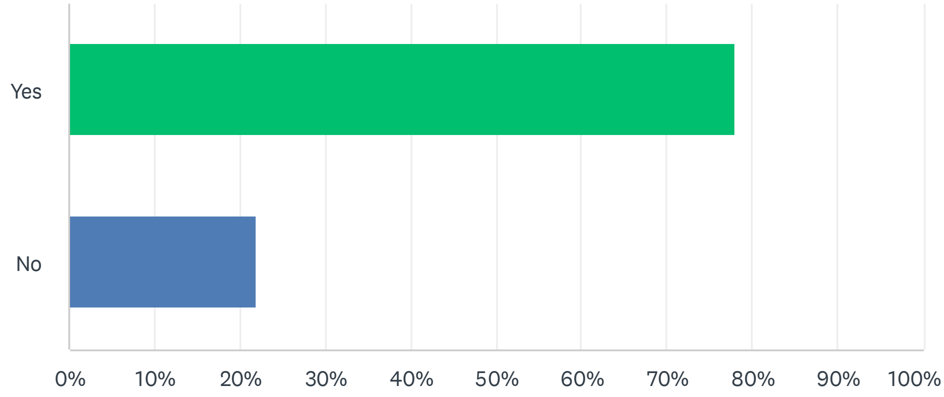

ANSWER CHOICES	RESPONSES	
Yes	78.14%	311
No	21.86%	87
TOTAL		398

Q62 Have you used ChatGPT in your academic/research work?

Answered: 398 Skipped: 17

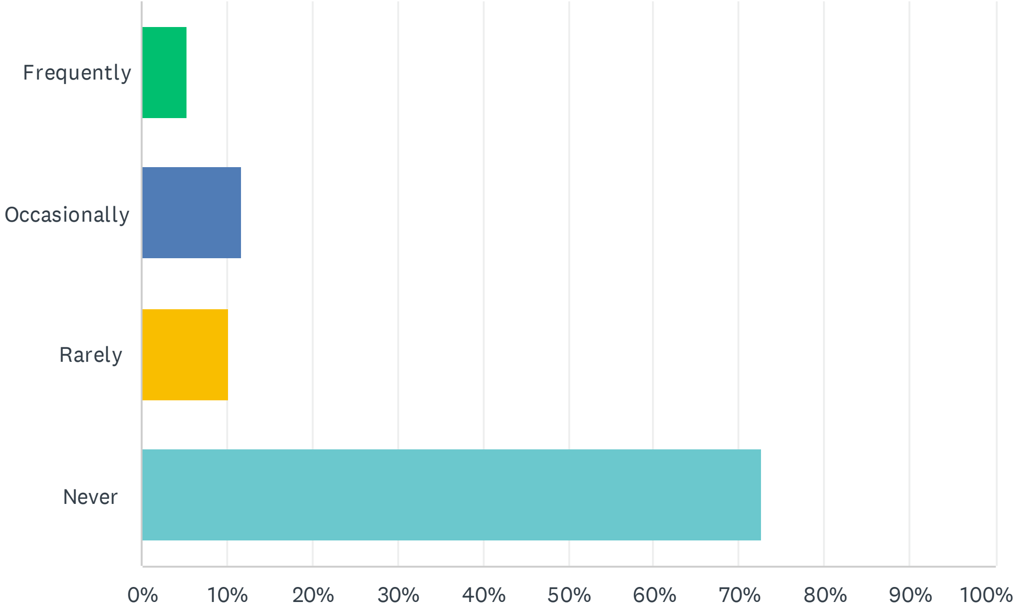

ANSWER CHOICES	RESPONSES	
Frequently	5.28%	21
Occasionally	11.81%	47
Rarely	10.30%	41
Never	72.61%	289
TOTAL		398

Q63 In what ways have you used ChatGPT for research purposes? (Select all that apply)?

Answered: 109 Skipped: 306

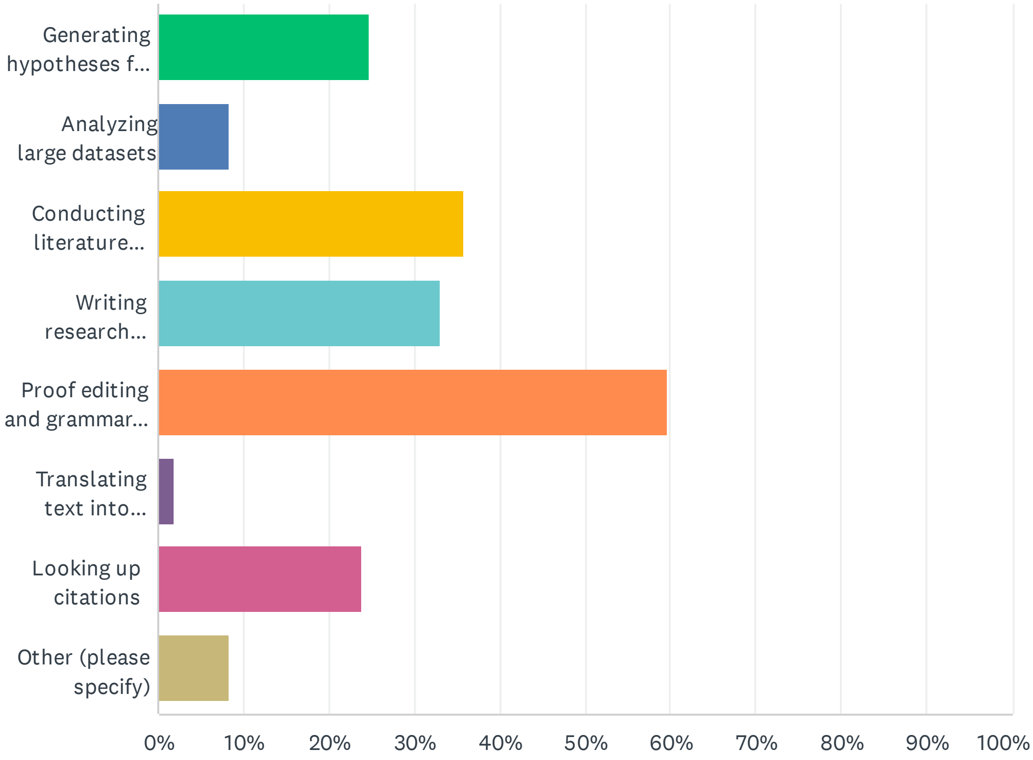

ANSWER CHOICES	RESPONSES	
Generating hypotheses for research studies	24.77%	27
Analyzing large datasets	8.26%	9
Conducting literature reviews	35.78%	39
Writing research articles or grant proposals	33.03%	36
Proof editing and grammar correction	59.63%	65
Translating text into English/another language	1.83%	2
Looking up citations	23.85%	26
Other (please specify)	8.26%	9
Total Respondents: 109		

Q64 Has ChatGPT improved your research productivity?

Answered: 109 Skipped: 306

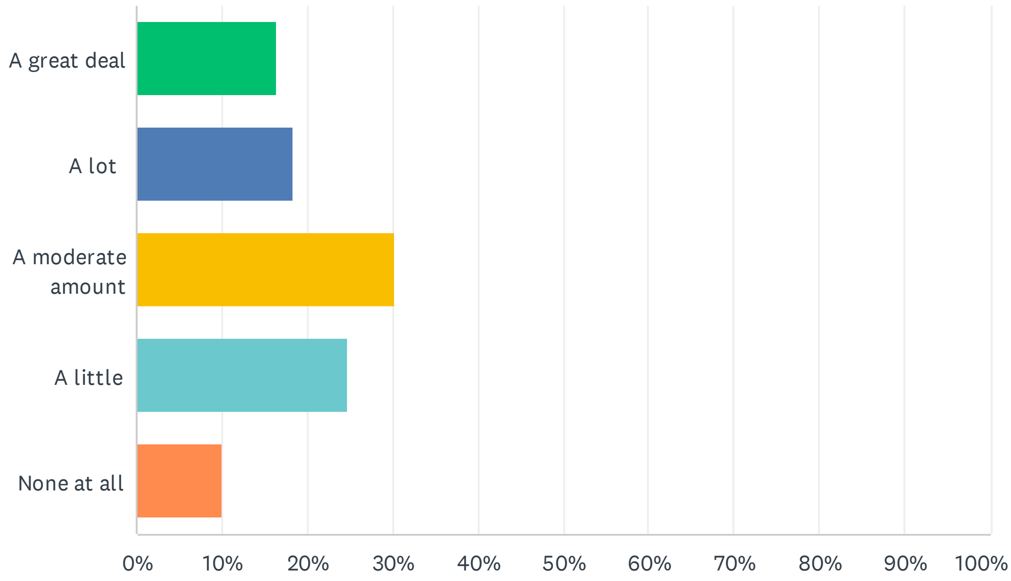

ANSWER CHOICES	RESPONSES	
A great deal	16.51%	18
A lot	18.35%	20
A moderate amount	30.28%	33
A little	24.77%	27
None at all	10.09%	11
TOTAL		109

Q65 In what capacity have you used ChatGPT for scientific or academic writing? (Select all that apply)

Answered: 109 Skipped: 306

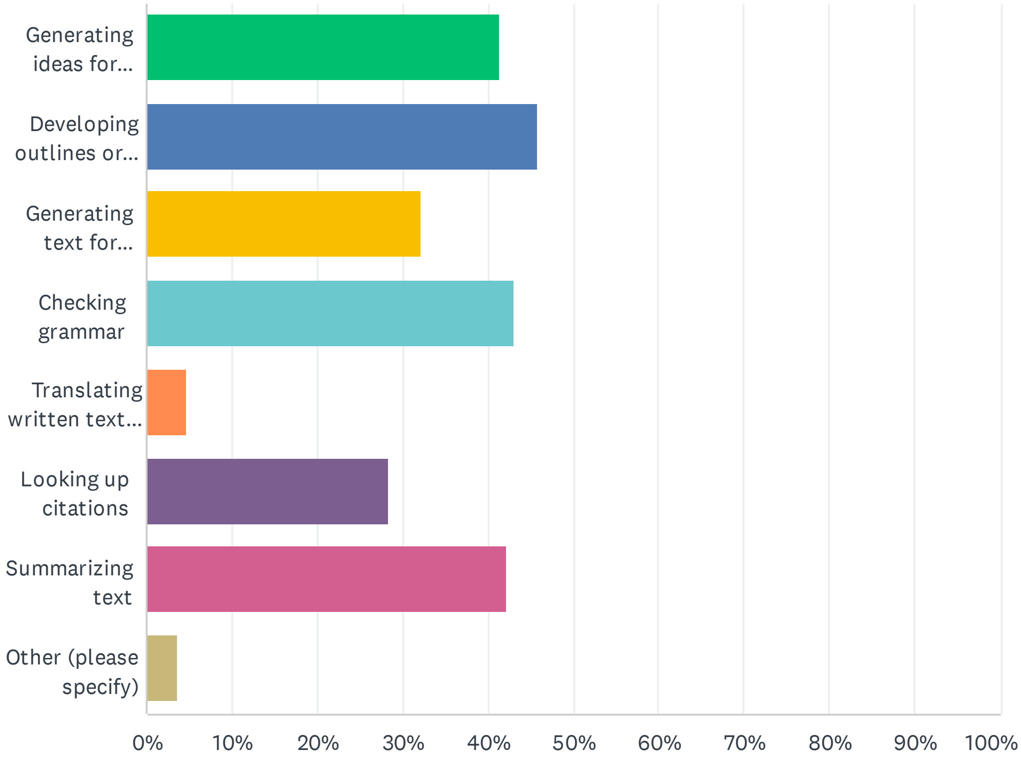

ANSWER CHOICES	RESPONSES	
Generating ideas for research or writing	41.28%	45
Developing outlines or structure for papers	45.87%	50
Generating text for manuscripts or reports	32.11%	35
Checking grammar	43.12%	47
Translating written text into English/another language	4.59%	5
Looking up citations	28.44%	31
Summarizing text	42.20%	46
Other (please specify)	3.67%	4
Total Respondents: 109		

Q66 How effective do you find ChatGPT for assisting with scientific or academic writing?

Answered: 109 Skipped: 306

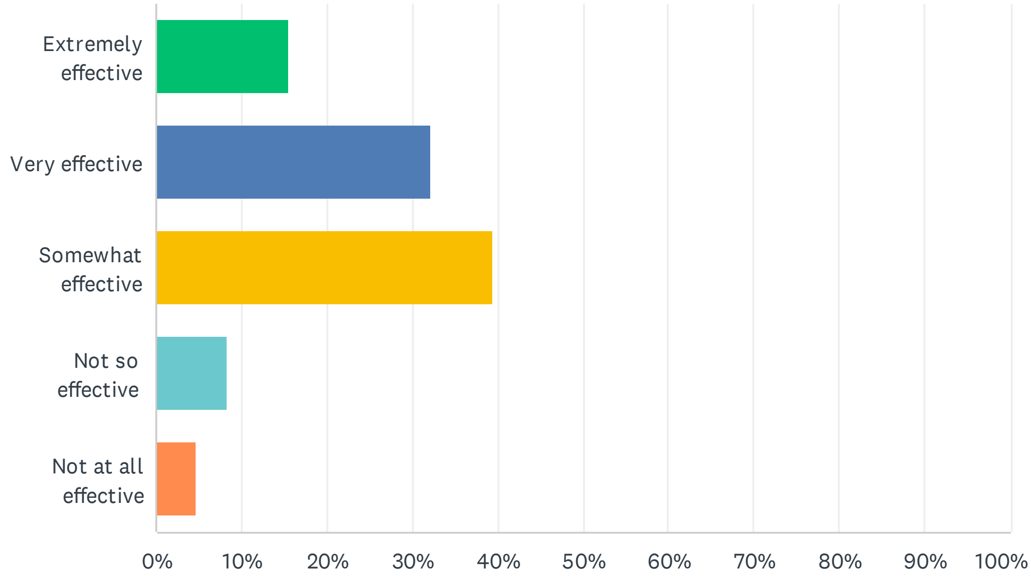

ANSWER CHOICES	RESPONSES	
Extremely effective	15.60%	17
Very effective	32.11%	35
Somewhat effective	39.45%	43
Not so effective	8.26%	9
Not at all effective	4.59%	5
TOTAL		109

Q67 ChatGPT will revolutionize the way scientific or academic writing is done?

Answered: 398 Skipped: 17

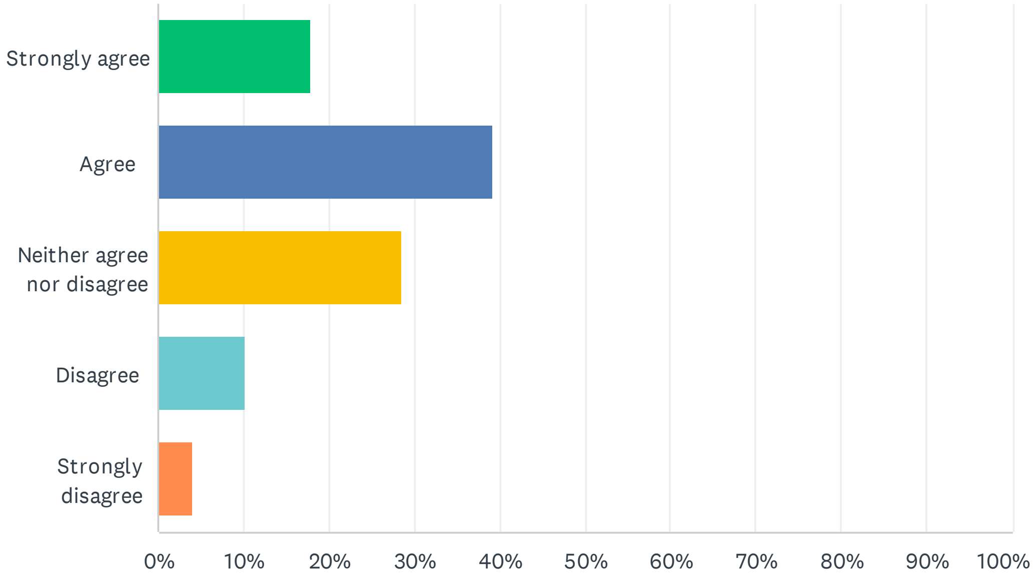

ANSWER CHOICES	RESPONSES	
Strongly agree	17.84%	71
Agree	39.20%	156
Neither agree nor disagree	28.64%	114
Disagree	10.30%	41
Strongly disagree	4.02%	16
TOTAL		398

Q68 What are some potential ethical concerns associated with the use of ChatGPT (or any other LLMs) in patient care and research? (Select all that apply)

Answered: 398 Skipped: 17

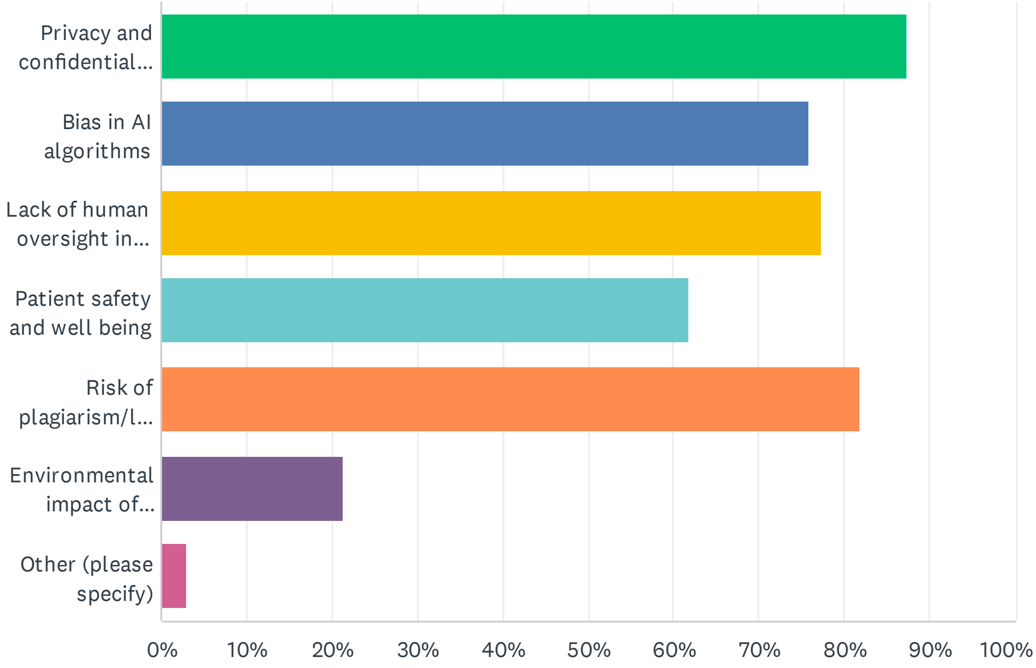

ANSWER CHOICES	RESPONSES	
Privacy and confidentiality of patients' data	87.44%	348
Bias in AI algorithms	75.88%	302
Lack of human oversight in decision making	77.39%	308
Patient safety and well being	61.81%	246
Risk of plagiarism/loss of academic integrity	81.91%	326
Environmental impact of ChatGPT (or any other LLMs)	21.36%	85
Other (please specify)	3.02%	12
Total Respondents: 398		

Q69 What are some measures that should be put in place to regulate the use of ChatGPT (or any other LLMs) in healthcare? (Select all that apply)

Answered: 398 Skipped: 17

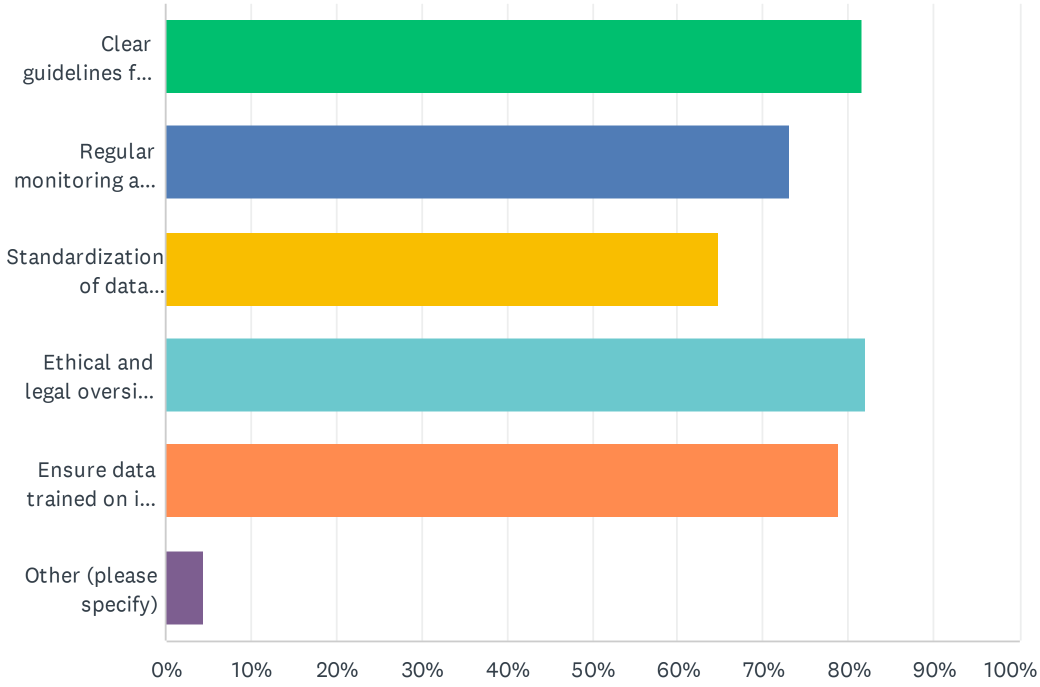

ANSWER CHOICES	RESPONSES	
Clear guidelines for use and oversight	81.66%	325
Regular monitoring and auditing of AI systems	73.12%	291
Standardization of data collection and analysis	64.82%	258
Ethical and legal oversight	82.16%	327
Ensure data trained on is up to date on clinical guidelines	78.89%	314
Other (please specify)	4.52%	18
Total Respondents: 398		

Q70 ChatGPT (or any other LLMs) outputs have not been validated, thus should not be used in clinical care

Answered: 398 Skipped: 17

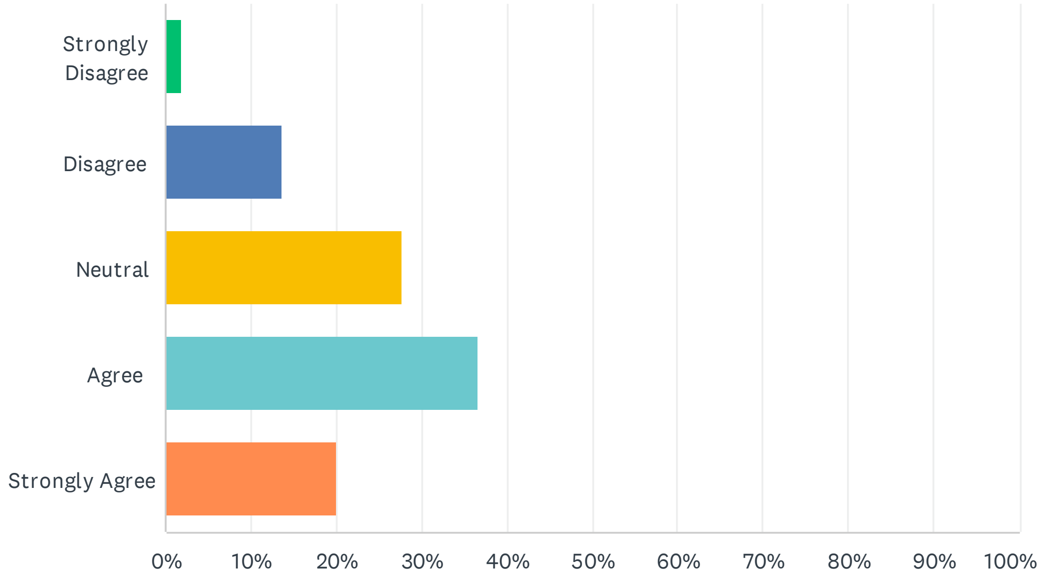

ANSWER CHOICES	RESPONSES	
Strongly Disagree	2.01%	8
Disagree	13.57%	54
Neutral	27.64%	110
Agree	36.68%	146
Strongly Agree	20.10%	80
TOTAL		398

Q71 What is your level of trust in ChatGPT's ability to provide information?

Answered: 398 Skipped: 17

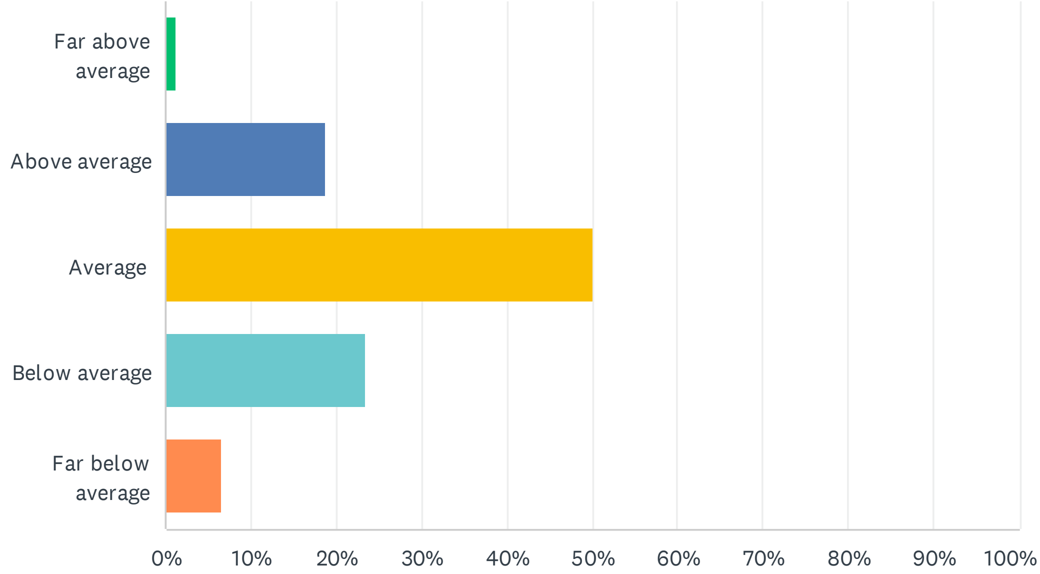

ANSWER CHOICES	RESPONSES	
Far above average	1.26%	5
Above average	18.84%	75
Average	50.00%	199
Below average	23.37%	93
Far below average	6.53%	26
TOTAL		398

Q72 Do you think that stakeholders (university policies, ect.) should make regulations and rules for the proper use and disclosure of ChatGPT?

Answered: 398 Skipped: 17

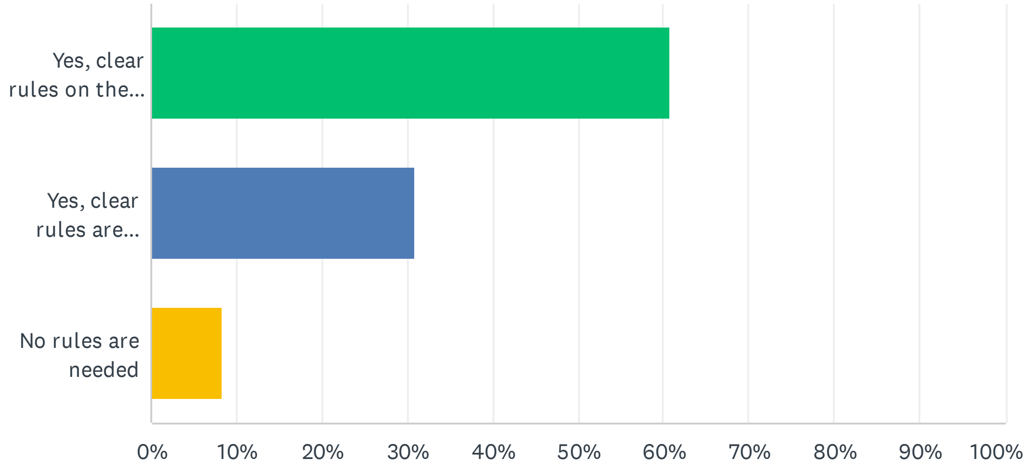

ANSWER CHOICES	RESPONSES	
Yes, clear rules on the use of ChatGPT are mandatory and the failure to comply should have consequences.	60.80%	242
Yes, clear rules are needed but the authors are free to follow or not	30.90%	123
No rules are needed	8.29%	33
TOTAL		398

Q73 Contact Information

Answered: 169 Skipped: 246

ANSWER CHOICES	RESPONSES	
Name (First and Last)	98.82%	167
Institution	96.45%	163
Twitter Handle	21.30%	36
Address 2	0.00%	0
City/Town	0.00%	0
State/Province	0.00%	0
ZIP/Postal Code	0.00%	0
Country	0.00%	0
Email Address	92.90%	157
Phone Number	0.00%	0
